# A multimodal adaptive optical microscope for in vivo imaging from molecules to organisms

**DOI:** 10.1038/s41592-026-03066-1

**Published:** 2026-05-22

**Authors:** Tian-Ming Fu, Gaoxiang Liu, Daniel E. Milkie, Xiongtao Ruan, Frederik Görlitz, Yu Shi, Valentina Ferro, Nikita S. Divekar, Wei Wang, Harrison M. York, Velat Kilic1, Matthew Mueller, Yajie Liang, Timothy A. Daugird, Maria Jose Gacha-Garay, Kathryn A. Larkin, Rebecca C. Adikes, Nathanael Harrison, Cyna Shirazinejad, Samara Williams, Jamison L. Nourse, Shu-Hsien Sheu, Liang Gao, Tongchao Li, Chandrani Mondal, Kemal Achour, Wilmene Hercule, Daniel R. Stabley, Kevin Emmerich, Peng Dong, David G. Drubin, Zhe J. Liu, Jeff S. Mumm, Minoru Koyama, Alison N. Killilea, Jose Javier Bravo-Cordero, C. Dirk Keene, Liqun Luo, Tomas Kirchhausen, Medha M. Pathak, Senthil Arumugam, James K. Nuñez, Ruixuan Gao, David Q. Matus, Benjamin L. Martin, Ian A. Swinburne, Eric Betzig, Wesley R. Legant, Srigokul Upadhyayula

**Affiliations:** 1 Janelia Research Campus, Howard Hughes Medical Institute, Ashburn, VA, USA.; 2 Department of Molecular and Cell Biology, University of California, Berkeley, Berkeley, CA, USA.; 3 Lampe Joint Department of Biomedical Engineering, University of North Carolina at Chapel Hill, Chapel Hill, NC, USA.; 4 Department of Chemistry, University of Illinois Chicago, Chicago, IL, USA.; 5 Monash Biomedicine Discovery Institute, Faculty of Medicine, Nursing and Health Sciences, Monash University, Clayton/Melbourne, Victoria, Australia.; 6 Department of Diagnostic Radiology and Nuclear Medicine, University of Maryland School of Medicine, Baltimore, MD, USA.; 7 Department of Pharmacology, University of North Carolina at Chapel Hill, Chapel Hill, NC, USA.; 8 Department of Biochemistry and Cell Biology, Stony Brook University, Stony Brook, NY, USA.; 9 Biophysics Graduate Group, University of California, Berkeley, Berkeley, CA, USA.; 10 Department of Physiology and Biophysics, Sue and Bill Gross Stem Cell Research Center, University of California, Irvine, Irvine, CA, USA.; 11 Department of Biology, Howard Hughes Medical Institute, Stanford University, Stanford, CA, USA.; 12 Department of Medicine, Division of Hematology and Oncology, The Tisch Cancer Institute, Icahn School of Medicine at Mount Sinai, New York, NY, USA.; 13 Neuroimaging Laboratory, Department of Developmental Neurobiology, St. Jude Children’s Research Hospital, Memphis, TN, USA.; 14 Wilmer Eye Institute and the Department of Ophthalmology, Johns Hopkins University School of Medicine, Baltimore, MD, USA.; 15 McKusick-Nathans Institute and the Department of Genetic Medicine, Johns Hopkins University School of Medicine, Baltimore, MD, USA.; 16 Department of Cell and Systems Biology, University of Toronto, Scarborough, Ontario, Canada.; 17 University of Washington BioRepository and Integrated Neuropathology (BRaIN) laboratory, Harborview Medical Center, Seattle, WA, USA.; 18 Program in Cellular and Molecular Medicine, Boston Children’s Hospital, Boston, MA, USA.; 19 Department of Pediatrics, Harvard Medical School, Boston, MA, USA.; 20 Department of Cell Biology, Harvard Medical School, Boston, MA, USA.; 21 Department of Biomedical Engineering, and Center for Complex Systems Biology, University of California, Irvine, Irvine, CA, USA.; 22 European Molecular Biology Laboratory Australia, Monash University, Clayton/Melbourne, Victoria, Australia.; 23 Department of Biological Sciences, University of Illinois Chicago, Chicago, IL, USA.; 24 Department of Physics, Howard Hughes Medical Institute, Helen Wills Neuroscience Institute, University of California, Berkeley, Berkeley, CA, USA.; 25 Molecular Biophysics and Integrated Bioimaging Division, Lawrence Berkeley National Laboratory, Berkeley, CA, USA.; 26 Chan Zuckerberg Biohub, San Francisco, CA, USA.; 27 Present address: Department of Electrical and Computer Engineering, and Omenn Darling Bioengineering Institute, Princeton University, Princeton, NJ, USA.; 28 Present address: Department of Physics and Astronomy, Western University, London, Ontario, Canada.; 29 Present address: Department of Genetics, University of North Carolina at Chapel Hill, Chapel Hill, NC, USA.; 30 Present address: Department of Biology, Siena College, Loudonville, NY, USA.; 31 Present address: Chan Zuckerberg Imaging Institute, Redwood City, CA, USA.; 32 Present address: Liangzhu Laboratory, MOE Frontier Science Center for Brain Science and Brain-machine Integration, State Key Laboratory of Brain-machine Intelligence, Zhejiang University, Hangzhou, China.; 33 Present address: Institute of Biomedical and Health Engineering, Shenzhen Institutes of Advanced Technology, Chinese Academy of Sciences, Shenzhen, China.; 34 Present address: Department of Molecular and Cell Biology, University of California, Berkeley, Berkeley, CA, USA.; 35 These authors contributed equally: Tian-Ming Fu, Gaoxiang Liu, Daniel E. Milkie, Xiongtao Ruan.

## Abstract

Understanding biological systems requires observing features and processes across vast spatial and temporal scales, spanning nanometers to centimeters and milliseconds to days, often using multiple imaging modalities within complex native microenvironments. Yet, achieving this comprehensive view is challenging because microscopes optimized for specific tasks typically lack versatility due to inherent optical and sample handling tradeoffs, and frequently suffer performance degradation from sample-induced optical aberrations in multicellular contexts. Here, we present Multimodal Optical Scope with Adaptive Imaging Correction (MOSAIC), a reconfigurable microscope that integrates multiple advanced imaging techniques including light-sheet, label-free, super-resolution and multiphoton, all equipped with adaptive optics. MOSAIC enables noninvasive imaging of subcellular dynamics in both cultured cells and live multicellular organisms, nanoscale mapping of molecular architectures across millimeter-scale expanded tissues and structural/functional neural imaging within live mice. MOSAIC facilitates correlative studies across biological scales within the same specimen, providing an integrated platform for broad biological investigation.

Light microscopy has shaped our view of how life works since scientists first began observing cells through a lens, revealing processes from single-molecule kinetics to in toto organismal development in model systems ranging from cultured cell lines to organoids, *Caenorhabditis elegans*, *Drosophila*, zebrafish and mice. Historically, this diversity drove the development of distinct microscope modalities, including widefield, confocal, light-sheet and two-photon microscopy, each optimized to meet specific needs^1,2^. Super-resolution (SR) variants^3,4^ of these have further expanded the range and power of optical microscopy.

However, optimization for one modality or one class of samples invariably comes with constraints that compromise the study of other systems. These constraints encompass not only optical characteristics such as field of view (FOV) and resolution, but also practical considerations such as sample mounting and environmental control. For example, high numerical aperture (NA) objectives optimize spatial resolution, but sacrifice FOV and working distance. Light-sheet microscopes enable low-phototoxicity volumetric imaging, but can require nontraditional mounting arrangements or complex relay optics in inverted systems^5^. Two-photon point scanning increases imaging depth, but often sacrifices speed and broad multicolor capability^6,7^. As such, a modern imaging core or well-equipped microscopy laboratory often supports several different instruments tailored to different tasks, multiplying cost and operational complexity. Furthermore, nearly all commercial microscopes that are the backbone of imaging cores ignore the ‘elephant in the room’: sample-induced optical aberrations that quickly compromise performance in multicellular applications.

To address these issues, we developed the Multimodal Optical Scope with Adaptive Imaging Correction (MOSAIC), a single microscope that reconfigures on demand to different imaging modalities, each optimized for a different class of specimens. We demonstrate MOSAIC across a broad range of biological systems and spatiotemporal scales, including single-molecule tracking in live adherent cells, organelle remodeling in both isolated cells and developing organisms, quantifying neural activity at single-spine resolution in the cortex of live mice and mapping neuronal ultrastructure across millimeters of cleared tissue. Collectively, these applications highlight MOSAIC’s ability to switch seamlessly between oblique transmitted light illumination for label-free contrast, widefield epifluorescence imaging, three-dimensional (3D) structured illumination microscopy (SIM), lattice light-sheet microscopy (LLSM), lattice light-sheet (LLS)-SIM, image scanning microscopy (ISM) and two-photon point-scanning microscopy for in vivo imaging. Notably, all modes can apply adaptive optical (AO) direct wavefront sensing^8^ correction to counter sample-induced aberrations and ensure optimal performance at depth. By reusing the same hardware and software across modes, MOSAIC reduces the overall cost and minimizes the spatial footprint relative to acquiring multiple separate instruments. It also enables quantitative comparisons of different imaging methods on the same biological sample and opens doors for adaptive imaging protocols that demand dynamically switching between modalities.

## Results

### Microscope design and characterization

MOSAIC was conceptualized as an evolution of the LLSM design incorporating two-channel AO correction (AO-LLSM)^9^. The original instrument used a 0.65-NA excitation lens and a 1.1-NA detection lens along with a 0.8-NA epifluorescence objective to view the sample from below^10^. While this arrangement optimized 3D spatial resolution, it also placed severe constraints on working distance and specimen geometries, typically limiting samples to a 5-mm diameter coverslip positioned between the objectives. For MOSAIC, we opted for a 0.6-NA excitation, a 1.0-NA detection and a coverslip-corrected 1.0-NA inverted epifluorescence objective (Supplementary Fig. 1a). While entailing a modest reduction in resolution^11^, these choices increase the effective working distance between the light-sheet objectives and the coverslip from near zero to 330 μm (Supplementary Fig. 1a), thereby accommodating 25-mm diameter coverslips for specimen mounting with an unobstructed scan range across its entire area. The two 1.0-NA detection objectives enable high-resolution, millimeter-scale FOV investigations across a variety of imaging modes (Supplementary Figs. 1b-e and 2-4). All three objectives and the specimen are housed within an environmental chamber with full control over temperature, perfusion and CO_2_. A fourth objective, also of 1.0 NA, resides in an upright station within MOSAIC for two-photon imaging of larger specimens such as live mice (Supplementary Fig. 1f).

To maximize the excitation power needed for large FOV and fast light-sheet imaging, MOSAIC uses a Powell/cylindrical lens combination (Supplementary Fig. 2a), concentrating laser power onto the long, narrow region of the spatial light modulator (SLM) to which the LLS pattern is written. Any combination of seven visible lasers can be used simultaneously, as we first split their post-Powell lens overlapping light sheets with a stack of parallel dichroic mirrors into seven independent parallel stripes on the SLM (Supplementary Fig. 5a). This facilitates independent phase pattern control of light-sheet tip, tilt and AO correction for each laser. After reflection off the SLM, the seven colors return through the same dichroic stack, recombining into an overlapped, collinear multicolor beam (Supplementary Fig. 5a,b), which reflects off a sample-conjugate resonant galvo (to reduce sample-induced shadowing artifacts^9^) and two pupil-conjugate galvos (for LLS axial alignment and lateral dither) before entering the excitation objective (Supplementary Fig. 2a). The resulting volumetric FOV (without tiling) for LLSM increases from 100 × ~30 × 100 μm^3^ in the previous implementation to 200 × ~30 × 20,000 μm^3^ in MOSAIC, and the imaging speed is more than doubled by the focused excitation and the use of two cameras (Supplementary Figs. 2b and 6b) for simultaneous two-color imaging.

AO-LLSM requires a substantial investment in hardware (for example, objectives, lasers, galvos, SLM, deformable mirror (DM), control electronics, sample stages and cameras). MOSAIC extracts maximal value from this investment by repurposing these components as needed for multiple imaging modes, all with AO correction: LLSM and LLS-SIM (Supplementary Figs. 2a,b and 6b), oblique transmitted light illumination (Supplementary Figs. 2c and 6c), widefield and 3D-SIM (Supplementary Figs. 2d and 6d), ISM (Supplementary Figs. 3a and 6e), two-photon Bessel beam light-sheet (Supplementary Figs. 3c and 7a,b), two-photon point-scanning microscopy (Supplementary Figs. 4a and 7d, in both inverted and upright configurations) and point-scanning photostimulation (Supplementary Figs. 4b and 7f). Optical toggles (Supplementary Fig. 5c) reconfigure the light path between modes in 2–5 s or reconfigure the imaging cameras as wavefront sensors during wavefront measurement. The entire instrument, including lasers, fits within a 1 × 1 × 1 m^3^ volume, promoting alignment stability (Supplementary Video 1). Pre-centered mirrors and lenses with no adjustment (Supplementary Fig. 5d) simplify assembly and alignment of MOSAIC by eliminating unnecessary degrees of freedom, further contributing to stability. Characterizations of the optical path as well as the experimentally measured point-spread function (PSF) and optical transfer function (OTF) for each mode are provided in Supplementary Figs. 2-4 and 8.

### Long-term multimodal imaging of cultured cell growth over vast fields of view

**Fig. 1 ∣:**
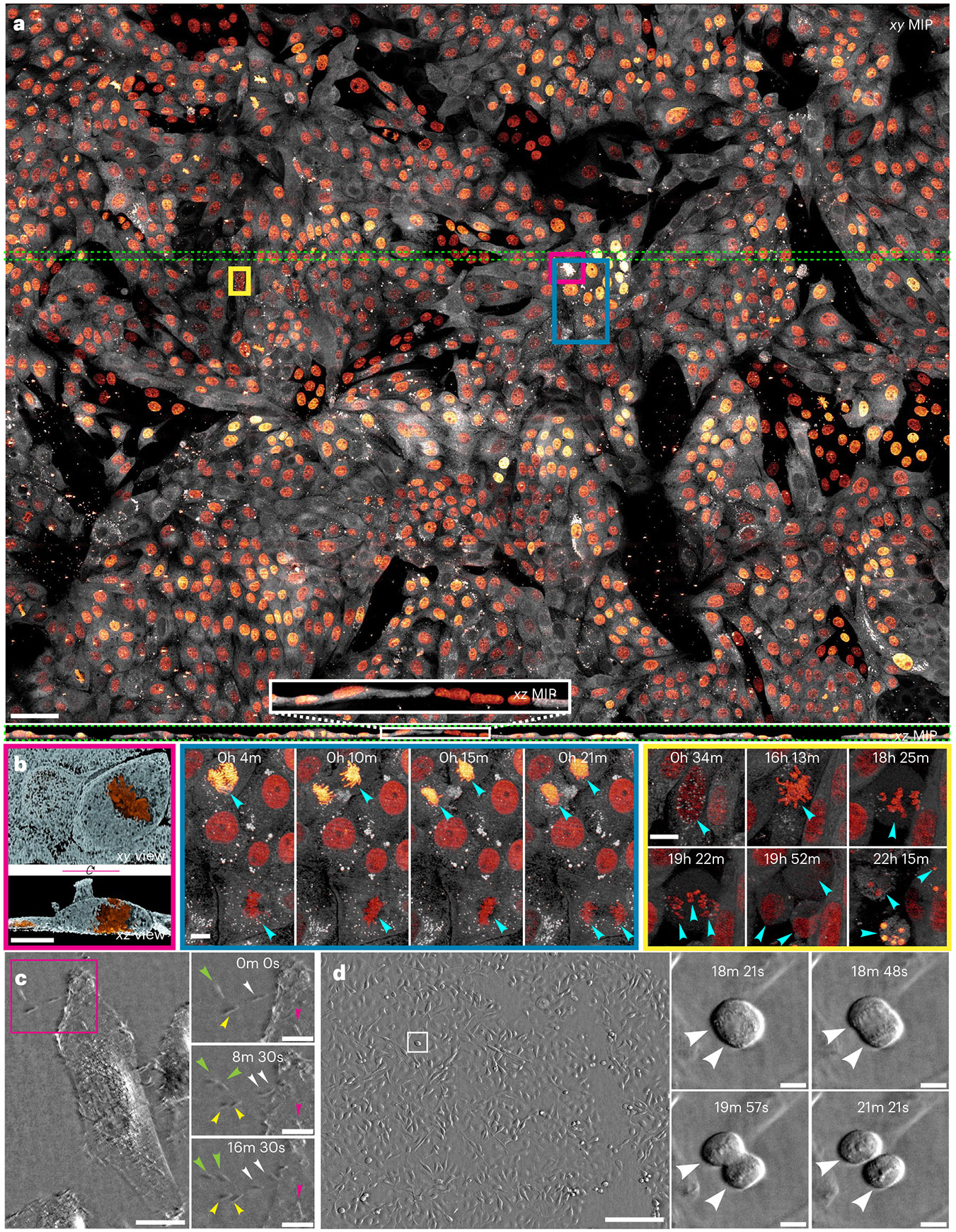
Large field-of-view dynamic imaging with 3D lattice light-sheet or 2D label-free oblique illumination microscopy. **a**, LLSM image from Supplementary Video 2 of LLC-PK1 cells expressing Calnexin–mEmerald (ER) and H2B–mCherry (nuclei). Maximum-intensity-projections (MIPs) show *xy* (top) and *xz* (bottom) views of the 1,000 × 750 × 10 μm^3^ volume. Scale bar, 50 μm. **b**, Zoomed-in views of cell division events from **a**. Magenta panel (left), volume rendering of a metaphase cell having a large ER protrusion; blue panel (middle), four points in nominal cell division from metaphase to telophase; yellow panel (right), six points during a rare tripolar mitotic event. Scale bar, 10 μm. **c**, Label-free OI imaging at 1 Hz (Supplementary Video 3) captures HeLa cell lamellipodial ruffling and replication of contaminating bacteria. Scale bar, 10 μm. Right, Magnified view (magenta box) of three bacterial division events (arrows). Scale bar, 2 μm. **d**, Tiled label-free imaging over a 1,218 × 975 μm^2^ field of live U2OS cells at 1 Hz. Scale bar, 200 μm. Right, zoomed-in view (white box) of a single dividing U2OS cell at four points, showing condensed chromosomes (arrows). Scale bar, 10 μm (right).

Even clonal populations of cultured cells exhibit considerable structural and functional heterogeneity^12^. A more comprehensive understanding of such cells requires mapping their phenotypic diversity by imaging many cells over multiple rounds of division within a single experiment. Using MOSAIC and the noninvasiveness of LLSM, we volumetrically imaged a ~1 × 0.75 mm^2^ field of LLC-PK1 cells stably expressing markers for the endoplasmic reticulum (ER) and nucleus at ~260 × 260 × 431 nm^3^ nominal resolution every 90 s over ~24 h (984 time points; Fig. 1a,b and Supplementary Video 2). The entire 49-TB dataset, which contains ~1.5 million nuclear image volumes (Supplementary Fig. 9a), was acquired at a peak rate of 4 TB h^−1^ and processed by PetaKit5D^13^.

The cell population plateaued upon reaching confluency ~12 h from initial imaging (Supplementary Fig. 9b and Supplementary Video 2), after which patches of cells exhibited occasional tissue-like behaviors such as epithelial detachment (Fig. 1a) and reattachment. While the great majority of mitotic events followed the archetypal pattern (Fig. 1b), we also identified atypical events such as a dividing cell with a large apical ER extrusion (Fig. 1b), and an example of tripolar mitosis. Such divisions have been observed previously^13,14^, but MOSAIC offers a more detailed four-dimensional (4D) view of this process, and its noninvasiveness enabled us to study the daughter cells through to their ultimate apoptosis (Fig. 1b). As seen in this example, the vast spatiotemporal range covered by MOSAIC (<10 ms to 24 h and 260 nm to 1 mm, or 10^7^ in time and 10^8^ in volume) enables quantitative measurement of normal phenotypic distributions of structure and dynamics across large cellular populations as well as identification and detailed investigation of potentially important outliers from such distributions.

To provide global cellular context complementary to the protein specificity of fluorescence, MOSAIC incorporates label-free oblique illumination (OI). Uniform laser illumination of the sample-conjugate SLM creates point excitation at the rear pupil of the excitation objective. Steering this point successively inward from the pupil edge creates widefield illumination intersecting the specimen at an angle relative to the collection cone of the inverted objective (Supplementary Figs. 1c and 10a,b) that initially enters, then is tangent to, and finally misses the cone (conditions that produce bright-field, DIC-mimicking oblique or dark-field images, respectively; Supplementary Fig. 10c). Imaging speed and FOV are only camera-limited, producing two-dimensional (2D) videos over a ~250 ×250 μm^2^ FOV at up to 100 Hz, ideal for monitoring highly dynamic events like membrane ruffling and host-pathogen interactions (Fig. 1c and Supplementary Video 3). Tiled acquisition enables studies over even larger FOVs, such as the migration and mitosis of cultured U2OS cells over a 1.218 × 0.975 mm^2^ FOV at 1 Hz for ~17 min (stitched dimensions, 1,000 time points; Fig. 1d and Supplementary Video 3). Global contrast over such broad areas provides simple assessment of overall sample health and identification of specific events for further investigation by other imaging modalities.

### Multimodal observation and optogenetic perturbation

MOSAIC permits interleaved LLSM and OI imaging in the same acquisition sequence, providing interleaved 3D fluorescence and 2D label-free data from the same specimen (Supplementary Figs. 11 and 12 and Supplementary Video 4). At the single cell level, we used this correlative approach to reveal 3D mitochondrial remodeling via LLSM at 56-s intervals over 2 h in the context of more rapid changes in the plasma membrane and shape of an hTERT-RPE1 cell (Supplementary Fig. 11 and Supplementary Video 4). Over a much larger FOV, we captured unlabeled intracellular events together with the 4D dynamics of the ER and Golgi apparatus for 2,000 time points at 16.3-s intervals (9 h total) through two-color LLSM (Supplementary Fig. 12 and Supplementary Video 4).

MOSAIC also incorporates point-scanned excitation through any of its three objectives for targeted optogenetic stimulation of the specimen (Supplementary Fig. 4b). To demonstrate, we induced localized actin polymerization, membrane ruffling and macropinosome engulfment^15-18^ in hTERT-RPE1 cells transiently expressing photoactivatable Rac1 (PA-Rac1–mCherry)^19^ by raster-scanning 445 nm light through the inverted objective over a region of interest for 30 s per frame (Supplementary Fig. 13a,b and Supplementary Video 5). Interleaving photoactivation with OI imaging allowed us to observe the induced subcellular dynamics within each activated region (Supplementary Fig. 13c).

### Multimodal live cell super-resolution microscopy

**Fig. 2 ∣:**
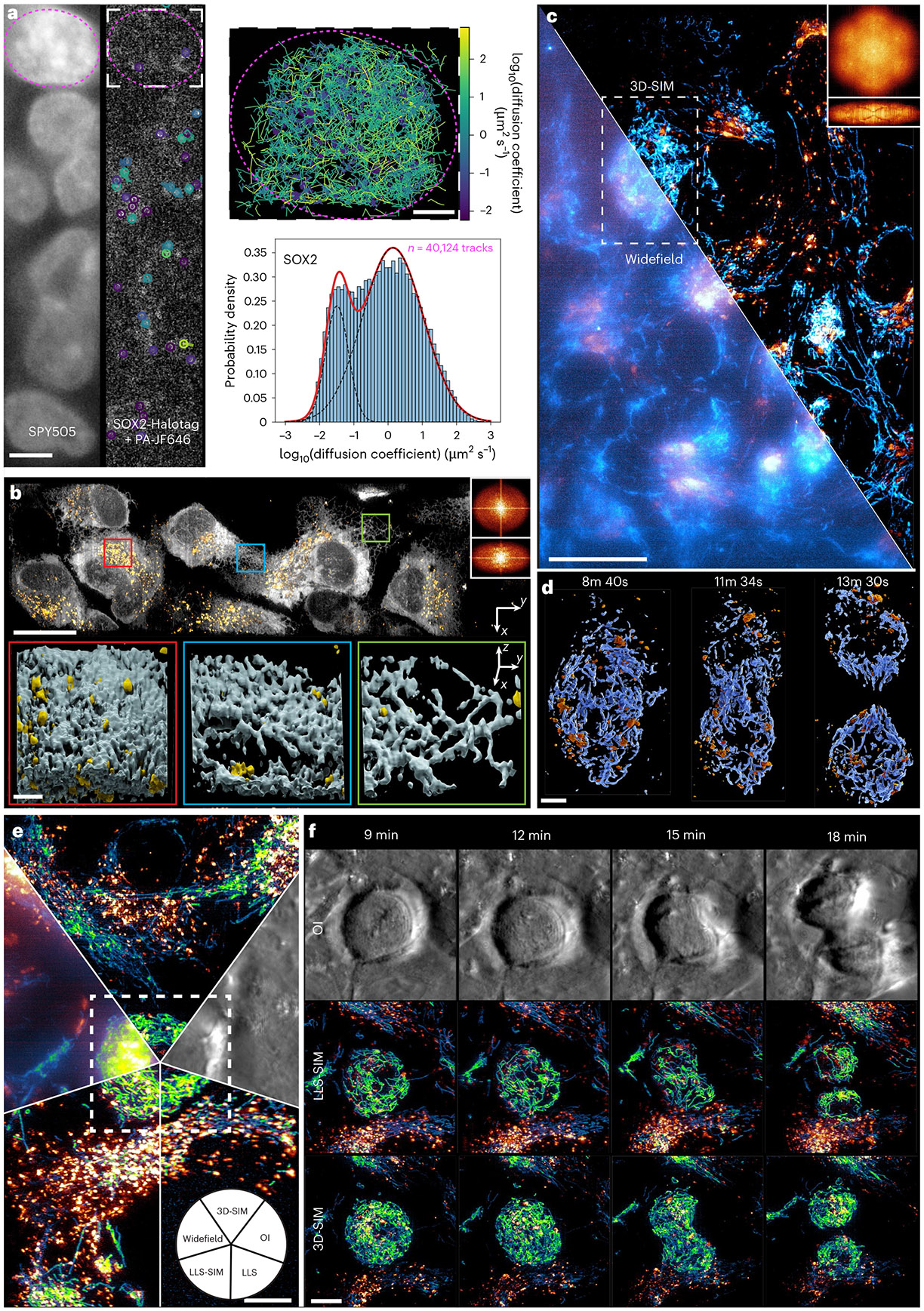
Multimodal super-resolution imaging of subcellular dynamics. **a**, Nuclear imaging (SPY 505) and single-particle tracking with LLSM, visualizing molecular trajectories and diffusion dynamics of SOX2 (HaloTag-PA-JF646) in mouse embryonic stem (mES) cells at 50 Hz. Scale bar (left), 5 μm. Scale bar (top right), 2 μm. The probability density histogram of log_10_ diffusion coefficients is binned from −3 to +3 in 0.1-unit increments. **b**, Top: LLS-SIM *xy* MIP of hTERT-RPE1 cells expressing ER (StayGold-ER, gray) and Golgi (β4Gal-HaloTag9 labeled with JFX549, orange) markers, from Supplementary Video 6. Insets, corresponding *xy* and *yz* OTFs. Scale bar, 25 μm. Bottom, volume renderings from boxed regions above showing ER and Golgi organization. Scale bar, 2 μm. **c**, Comparison of widefield and 3D-SIM imaging in hTERT-RPE1 cells with mitochondria (COX8a-StayGold, cyan) and Golgi marker (orange). Scale bar, 25 μm. Insets, 3D-SIM OTFs. **d**, Timelapse 3D-SIM (Supplementary Video 7) captures mitochondrial and Golgi dynamics during cell division (white box from **c**). Scale bar, 5 μm. **e**, Correlative optical microscopy applying multiple imaging modalities (widefield, 3D-SIM, OI, LLS and LLS-SIM) to a dividing hTERT-RPE1 cell with labeled mitochondria (blue–green) and Golgi (orange), from Supplementary Video 8. Scale bar, 10 μm. **f**, Timelapse sequence of the correlative imaging shown in **e**. Scale bar, 10 μm.

MOSAIC incorporates several forms of SR microscopy to cover the tradeoffs between resolution, speed and phototoxicity: single-molecule tracking (SPT) and localization microscopy (SMLM); ISM; and SIM. To demonstrate, we first combined LLSM with SPT and measured the kinetics of SOX2 transcription factor molecules labeled with PA-JF646 across a field of mouse embryonic stem cell nuclei at a median precision ± s.d. of 25 ± 4 nm and speed of 50 Hz (Fig. 2a). Trajectory analysis and fitting to an anomalous diffusion model yielded a distribution well described by a two-component Gaussian mixture model with diffusion coefficients and relative populations between the ‘DNA bound’ and ‘freely diffusing’ states consistent with previous findings^20^.

Trading resolution for speed and noninvasiveness, MOSAIC can combine LLSM and SIM, extending resolution in the laterally structured direction of the light sheet from 260 nm to ~180 nm (Supplementary Fig. 8)^10^. With this modality we imaged the 4D dynamics of the ER and Golgi in cultured hTERT-RPE1 cells over a 50 × 188 × 10 μm^3^ FOV every 10 s or 11 × 188 × 10 μm^3^ FOV every 2 s for 1,500 time points with minimal photobleaching (where the mean integrated FOV intensity remained within ±s.d. of the baseline throughout). The speed, resolution and duration of this imaging allowed us to observe the continuous extension and retraction of individual ER tubules, an ER tunnel passing through the cell nucleus, and the intercalation and migration of fragmented Golgi compartments within the 3D ER network (Fig. 2b, Supplementary Fig. 14 and Supplementary Video 6).

For live imaging with 2× resolution extension in all three dimensions, MOSAIC offers widefield 3D-SIM through the inverted objective. We first demonstrated this mode by imaging mitochondrial and Golgi 4D dynamics in hTERT-RPE1 cells across a 160 × 100 × 10 μm^3^ FOV at 57-s intervals for 70 time points (Fig. 2c,d, Supplementary Video 7 and Supplementary Fig. 15). To achieve both faster and longer imaging, we then reduced the number of raw images in 3D-SIM reconstruction from 15 in three directions to 10 in two directions and used computational denoising^21^ (Supplementary Fig. 16) to maintain accurate reconstructions at a lower signal-to-noise ratio (SNR). This enabled prolonged (10.7 h) observation of mitochondrial events such as fission, fusion, active transport and clustering, for 1,200 time points at 32-s intervals (Supplementary Fig. 17a and Supplementary Video 7) across a mixed field of quiescent, mitotic (Supplementary Fig. 17b) and migrating (Supplementary Fig. 17c) cells.

While the inclusion of SPT, LLS-SIM and widefield 3D-SIM gives the user flexibility to choose among the tradeoffs in live SR imaging (for example, Supplementary Video 8), MOSAIC also allows them to be used in concert or with other modes in interleaved form. To demonstrate, we imaged a dividing hTERT-RPE1 cell labeled for mitochondria and Golgi. Every 3 min, we acquired 1,000 OI images, 20 LLSM volumes and 1 volume each of LLS-SIM and 3D-SIM (Fig. 2e,f and Supplementary Video 8). This correlative protocol captures different perspectives of the same event across multiple spatial and temporal scales.

### Structural 3D SR imaging over large fields of view

**Fig. 3 ∣:**
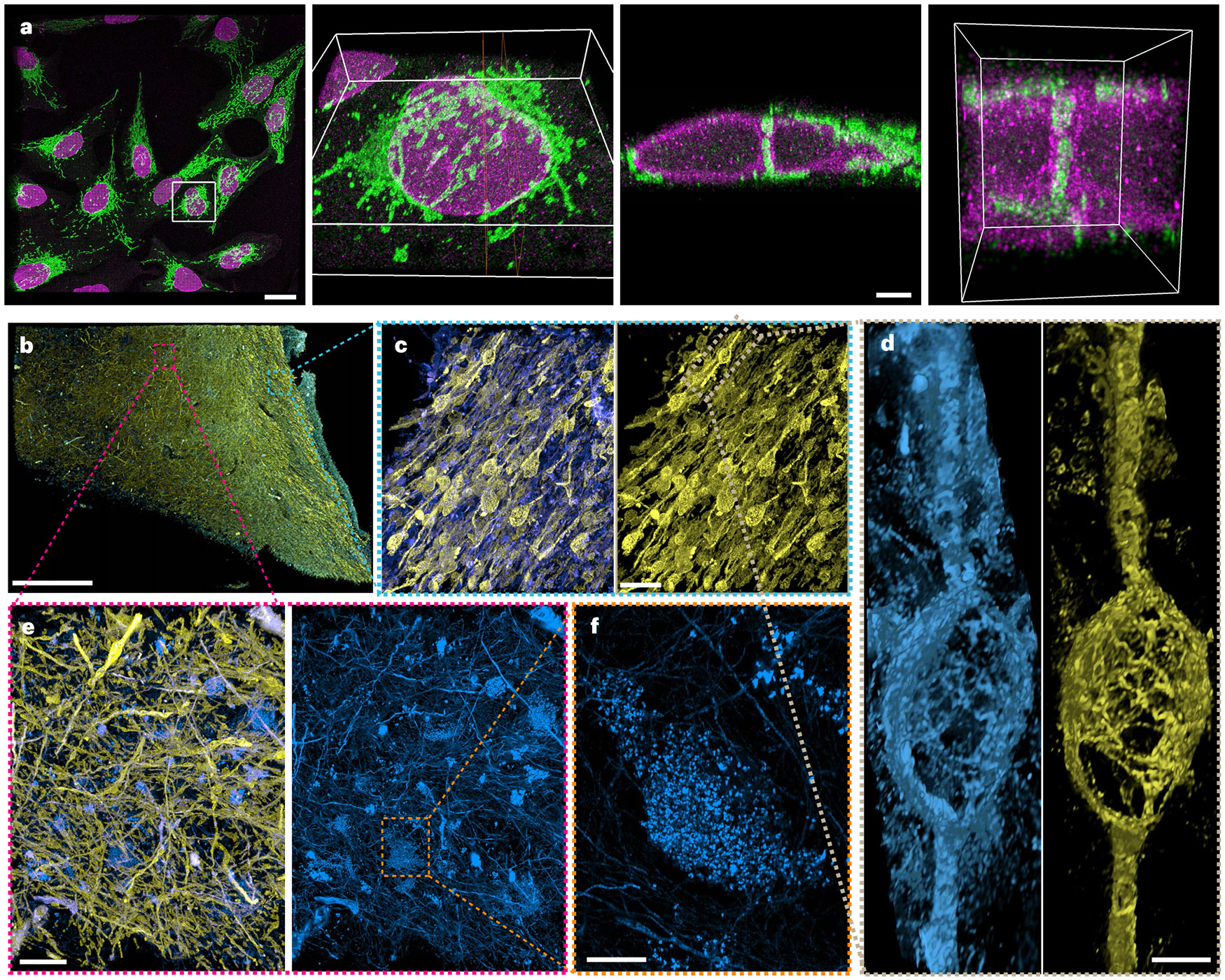
Volumetric imaging with nanoscale resolution. **a**, Two-color 3D DNA-PAINT of the mitochondrial marker TOMM20 and the nuclear envelope protein marker Lamin A/C in U2OS cells. Left to right, overview MIP of a 180 × 200 × 17 μm^3^ FOV (scale bar, 20 μm), zoomed-in 3D rendering of a single cell (boxed region, 28 × 23 × 5.4 μm^3^), its *xz* orthoslice (scale bar, 2 μm) and the close-up of nuclear invaginations (boxed region, 4.7 × 4.5 × 5.4 μm^3^). See also Supplementary Video 9. **b**, ExLLSM MIP overview of a 2,000 × 2,375 × 98 μm^3^ human hippocampal tissue section from a patient with AD after 4× expansion. NF-200 (blue) and MBP (yellow) label neurofilaments and myelin sheaths, respectively. Scale bar, 500 μm. **c**, Zoomed view from **b** showing neurofilament and myelin sheath ballooning. Scale bar, 20 μm. **d**, Nanoscale structure of axon and myelin sheath blebs. Scale bar, 2 μm. **e**, Zoomed-in region from **b** highlighting clustering of NF-200 protein. Scale bar, 20 μm. **f**, Nanoscale structure of an individual NF-200 cluster. Scale bar, 2 μm. Scale bars throughout represent pre-expansion dimensions.

The stability, large FOV and 25-mm *xy* scan range of MOSAIC, coupled to GPU-accelerated analysis pipelines such as PetaKit5D, facilitate 3D rapid SR structural imaging over large volumes. Applied to two-color 3D LLSM-PAINT^22^, we simultaneously imaged the nuclear envelope (specifically, Lamin A/C) and the mitochondrial outer membrane (TOMM20) with DNA probes^23^ conjugated to Cy3B and ATTO655, respectively, at median localization precisions ± s.d. of 16 ± 9 and 23 ± 1 nm laterally and 136 ± 81 and 178 ± 49 nm axially, over a 180 × 200 × 17 μm^3^ volume (Fig. 3a and Supplementary Video 9). The volume was sufficiently large to catch the unusual morphology of a mitochondrion extending through a tubular invagination of the nuclear envelope (Fig. 3a).

Much larger samples, including tissues, can be imaged in 3D at lower but still nanoscale resolution with expansion microscopy (ExM)^24^. Using LLSM^25^, we imaged immunostained myelin sheaths (myelin basic protein (MBP)) and axons (neurofilament-200 (NF-200)) at 4× expansion across a 2,000 × 2,375 × 98 μm^3^ section (pre-expansion) of human hippocampal brain tissue excised from a 90-year-old patient with Alzheimer’s disease (AD) (Fig. 3b-f and Supplementary Video 10). The MOSAIC platform allowed us to capture this 37-TB dataset in 8.3 h (peaking at ~335 million voxels per second per camera), approaching the peak acquisition speed of modern sCMOS cameras (~473 million voxels per second on a Hamamatsu ORCA-Fusion). The data revealed numerous examples of aging pathologies (Fig. 3c-f) such as clusters of dystrophic neurites, characterized by localized ‘ballooning’ of the myelin sheath^26^, resembling previous observations in aged macaques^27^. These varicosities exhibited distended neurofilaments, potentially representing axons delaminated from the surrounding myelin, which in turn appeared torn or shredded (Fig. 3d). In another region, we observed large punctate clusters of NF-200 consistent with Wallerian-like axonal degeneration (Fig. 3e)^28^. These may represent residual debris after immune-provoked phagocytosis of axons by microglia or oligodendrocyte precursor cells (Fig. 3f)^29-31^. Notably, the large physical separation between these two pathologies emphasizes the value of rapidly imaging mm-scale swathes of tissue to comprehensively explore the diverse morphologies hidden within them.

### Adaptive optical LLSM in living organisms

The cellular phenotypes we observe are the result of gene expression, which is regulated in part by external environmental cues. Therefore, we can only fully trust the physiological relevance of our observations when we image cells natively embedded within the organism in which they evolved. The challenge then is that sample-induced optical aberrations varying in space and time across the organism degrade image resolution and signal. In MOSAIC, we correct these with direct wavefront sensing AO^8^.

**Fig. 4 ∣:**
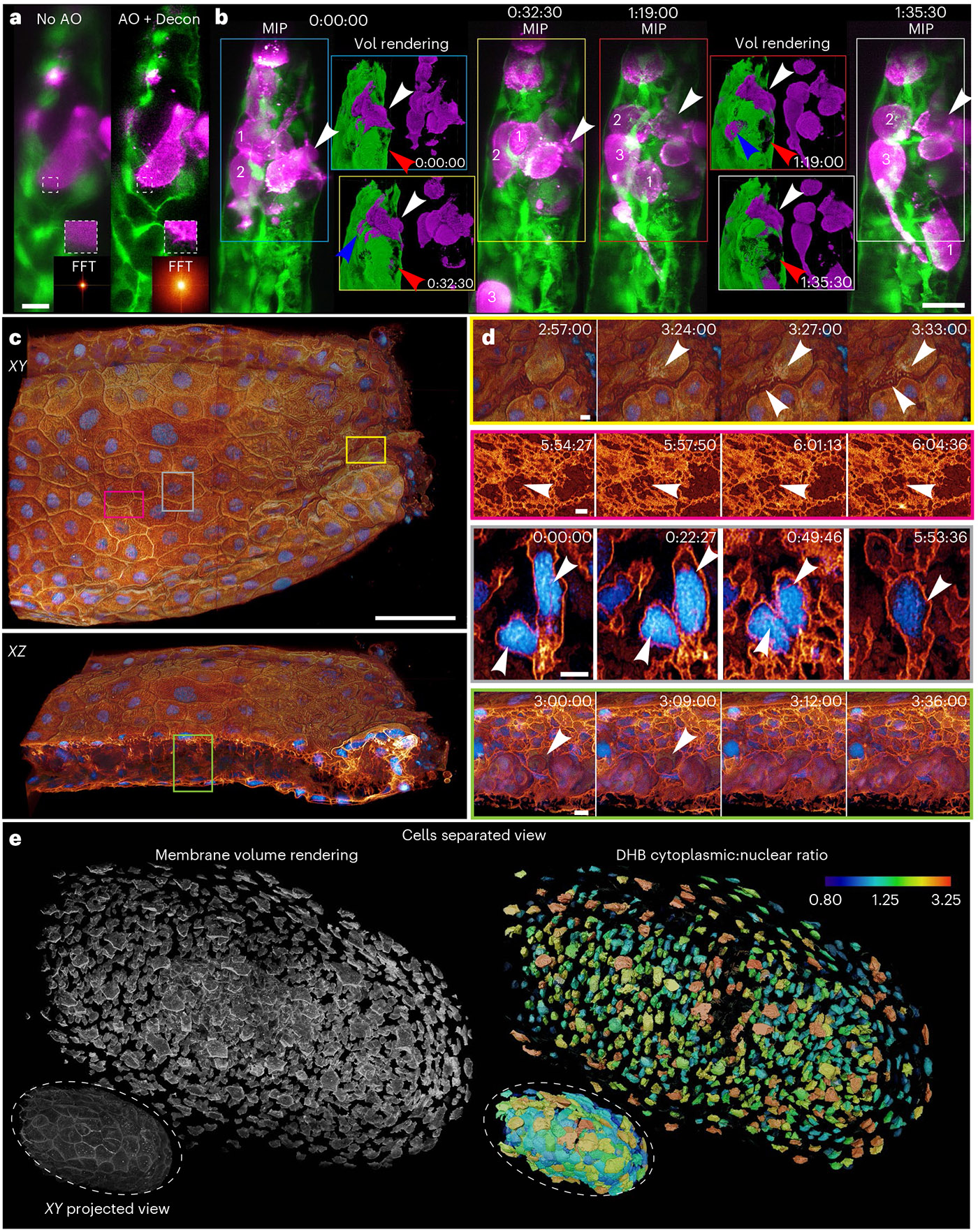
Observing cellular and subcellular dynamics within zebrafish embryos. **a**, Comparison of LLSM imaging in a zebrafish xenograft system (55 × 183 × 50 μm^3^) showing actin-labeled MDA-MB-231 human breast cancer cells (magenta) within the zebrafish vasculature (green), both without AO (left) and with AO correction plus deconvolution (right). Insets, corresponding Fourier spectra (at gamma = 0.3) of the magenta channel. Scale bar, 10 μm. **b**, Timelapse imaging of **a** capturing cancer cell dynamics and vascular damage during extravasation. Scale bar, 20 μm. **c**, Zebrafish tail fin volume (216 × 272 × 37 μm^3^) at 66 h post-amputation showing plasma membranes and nuclear histones. Scale bar, 50 μm. **d**, Cellular and subcellular events during the initial stages of regeneration after amputation (Supplementary Video 11), showing: extracellular vesicle release from a cell adjacent to the cut site (yellow box); anchoring fibril dynamics in the epidermal basement membrane (red box); a mesenchymal cell fusion event (gray box); and a transiently trapped red blood cell during remodeling of the caudal vascular plexus (green box). Scale bar, 5 μm. **e**, Visualization of cell cycle state across the fin, based on the cytoplasmic-to-nuclear fluorescence ratio of CDK biosensor DNA Helicase B (DHB), in segmented and computationally separated cells (216 × 173 × 37 μm^3^ pre-separation, inset). Peripheral cells have the highest fraction in G2 (Supplementary Video 12).

AO as applied to LLSM^9^ requires independent correction for the orthogonal excitation and detection pathways. However, once implemented, it delivers high-resolution 4D videos of dynamic subcellular processes in transparent organisms such as zebrafish embryos (Fig. 4). For example, over a FOV sufficiently small that a single pair of excitation and detection corrections suffices (a single isoplanatic patch), MOSAIC AO-LLSM provided detailed views of human breast cancer cells (MDA-MB-231) interacting with the zebrafish vasculature with full recovery of all spatial frequencies within the diffraction limit (Fig. 4a). Imaging over 90 min, we observed the free flow or slow migration of multiple cancer cells along the vasculature as well as an extravasation event, where an MDA-MB-231 cell begins to breach the vessel wall, leading to a tear in the vasculature itself (Fig. 4b).

To cover FOVs larger than a single isoplanatic patch, tiling is required, with independent AO corrections applied to each tile. To demonstrate, we studied post-amputation tail fin regeneration in a zebrafish larva at 48 h post-fertilization (hpf) over a 216 × 272 × 37 μm^3^ volume extending back from the wound (comprising four tiles) at 3-min intervals for 12.5 h (250 time points; Fig. 4c and Supplementary Video 11). This revealed a wide array of subcellular events, including secretion of microvesicles by cells proximal to the wound, dynamic remodeling of anchoring fibrils connecting the epidermal basement membrane to the dermal layers, mesenchymal cell fusion (plasma membrane followed by nuclear fusion) and transient erythrocyte trapping within the remodeling vasculature (Fig. 4d).

AO-LLSM also enables quantitative ratiometric biosensor imaging in vivo. We performed three-color imaging in the tailbud of a zebrafish embryo at 18 hpf expressing markers for membrane, histones and a cyclin-dependent kinase (CDK) activity sensor^32^ (comprising four tiles; Fig. 4e and Supplementary Video 12). The cytoplasmic-to-nuclear ratio of the sensor quantitatively reports cell cycle state, increasing as cells progress through interphase from G1/S into G2. Our analysis revealed spatial variations in cell cycle state across the tailbud, consistent with previous studies using either fluorescent ubiquitination-based cell cycle indicator (FUCCI)^33^ or a CDK activity sensor imaged by spinning disk confocal microscopy^32^. Cells near the periphery exhibited higher ratios indicative of a higher fraction in G2 (Fig. 4e and Supplementary Video 12), relative to those in the interior.

Notably, AO-LLSM can also be applied to other living multicellular systems, as we demonstrate for *Drosophila* (Supplementary Fig. 18a), *C. elegans* (Supplementary Fig. 18b) and human brain organoids (Supplementary Fig. 18c and Supplementary Video 13).

### Live adaptive optical SR imaging in zebrafish

SR reconstruction algorithms often assume a diffraction-limited, spatially invariant PSF^34,35^, which is distorted by tissue-induced aberrations. These aberrations also quickly attenuate higher spatial frequencies in the OTF to below the noise floor, rendering them useless for resolution extension and further contributing to reconstruction artifacts. AO in such cases is essential.

**Fig. 5 ∣:**
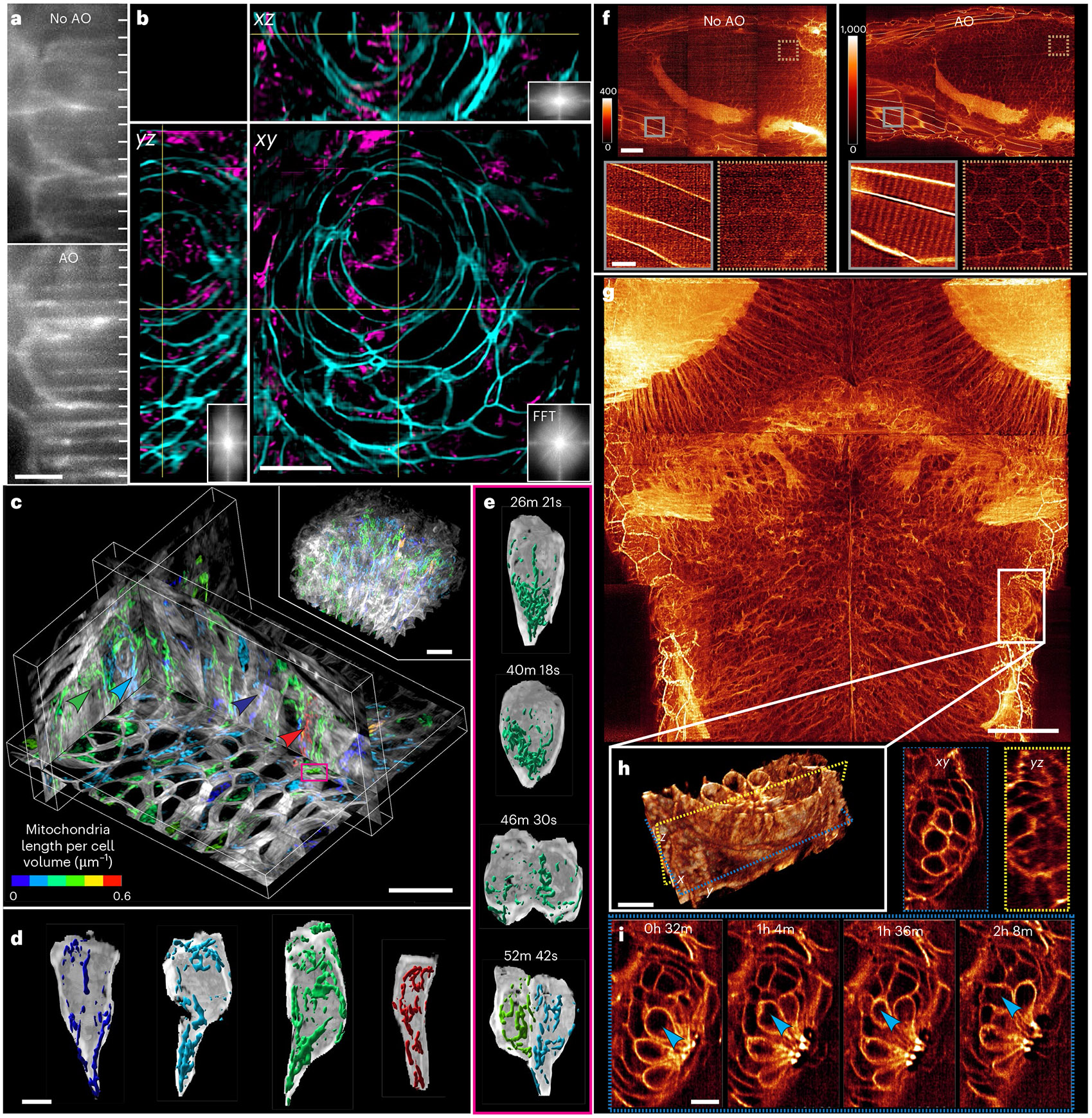
Super-resolution imaging in vivo. **a**, MIPs of raw zebrafish membrane images with LLS-SIM illumination without (top) and with AO (bottom). Ticks show the expected positions of the LLS pattern excitation maxima. Scale bar, 5 μm. **b**, Orthoslices (180-nm thick) in *xy*, *xz* and *yz* from an AO-LLS-SIM reconstruction (61 × 57 × 40 μm^3^) in the eye of a 14 hpf zebrafish embryo expressing mitochondrial (magenta) and plasma membrane (cyan) markers. Scale bar, 10 μm. Top left inset, Fourier spectrum of reconstructed mitochondria (Supplementary Video 14). **c**, AO-LLS-SIM MIPs of orthogonal slabs (3-μm thick) in the hindbrain from a 56 × 56 × 40 μm^3^ volume (inset) in a 14 hpf zebrafish with mitochondria in RGB colors and membrane in gray. Mitochondria in each cell are color-coded by the ratio of total mitochondrial length to cell volume. Scale bar, 10 μm. **d**, Cutaway view of segmented mitochondria in four different segmented cells. Scale bar, 4 μm. **e**, Cutaway view of one cell from the volume, showing mitochondrial rearrangements to the daughter cells during division. Scale bar, 4 μm. **f**, Top, ISM MIP views before and after AO correction of a 0.324 μm *xy* orthogonal slab within a larger 336 × 319 × 84 μm^3^ image volume spanning brain, muscle and notochord in a membrane-labeled 7 dpf zebrafish. Scale bar, 50 μm. Bottom, zoomed-in views comparing muscle and neural progenitor cells imaged by ISM with and without AO (Supplementary Video 16). **g**, AO-ISM MIP (354 × 332 × 16.3 μm^3^) of a dorsal-mounted, membrane-labeled, 7 dpf zebrafish. Scale bar, 50 μm. **h**, Volume rendering with *xy* and *yz* orthoslices through a neuromast. Scale bar, 10 μm. **i**, Timelapse AO-ISM showing *xy* orthoslices of a migrating cell in the neuromast. Scale bar, 10 μm.

To demonstrate, we applied AO to LLS-SIM and imaged both cell membranes and mitochondria in the eye of a zebrafish embryo at 14 hpf. Applying a 3 × 1 × 2 pattern of tiles to account for variations in aberration across the eye, we recovered the high modulation contrast from the LLS pattern (Fig. 5a) needed for accurate SIM reconstruction of membrane contours and densely packed mitochondria (Fig. 5b, Supplementary Fig. 19 and Supplementary Video 14) with resolution extension in the patterned direction (Fig. 5b). Next, we used AO-LLS-SIM with frame-by-frame denoising to compensate for photobleaching (Supplementary Fig. 20), and visualized subcellular mitochondrial dynamics in the zebrafish brain at 14 hpf for 60 time points at 93-s intervals (Supplementary Video 14). The high resolution of AO-LLS-SIM allowed us to accurately segment all mitochondria throughout the image volume (Supplementary Fig. 21) and study relationships between their morphologies and the volumes of the cells in which they reside (Fig. 5c,d), including how they partition between daughter cells during division (Fig. 5e and Supplementary Video 14).

For sufficiently large or oddly shaped specimens, or those having localized regions of large scattering or absorption, it can be difficult to find an orientation and position where the dual objectives of LLSM can both have optical access to the region of interest (Supplementary Fig. 1b). In such cases, single-objective epi-illumination can sometimes provide a workable alternative (Supplementary Fig. 1d). In particular, ISM with line illumination combines the benefits of rapid volumetric imaging with resolution superior to confocal microscopy in the scan direction (Supplementary Fig. 22 and Supplementary Video 15), albeit with far faster photobleaching and poorer axial resolution than LLSM. For imaging in multicellular organisms such as zebrafish, AO is needed to recover the high resolution offered by ISM (Fig. 5f and Supplementary Video 16). AO-ISM imaging from the dorsal side of a zebrafish at 7 days post-fertilization (dpf) over 354 × 332 × 16.3 μm^3^ with 4 × 3 × 1 AO corrective tiles (Fig. 5g) allowed us to capture developmental changes such as cell migration within a pair of laterally symmetric neuromast organs (Fig. 5h,i) for 12 time points over 6 h (Supplementary Video 16) across a broad region encompassing the hindbrain, eyes and spine.

### Adaptive optical two-photon imaging in living tissue

**Fig. 6 ∣:**
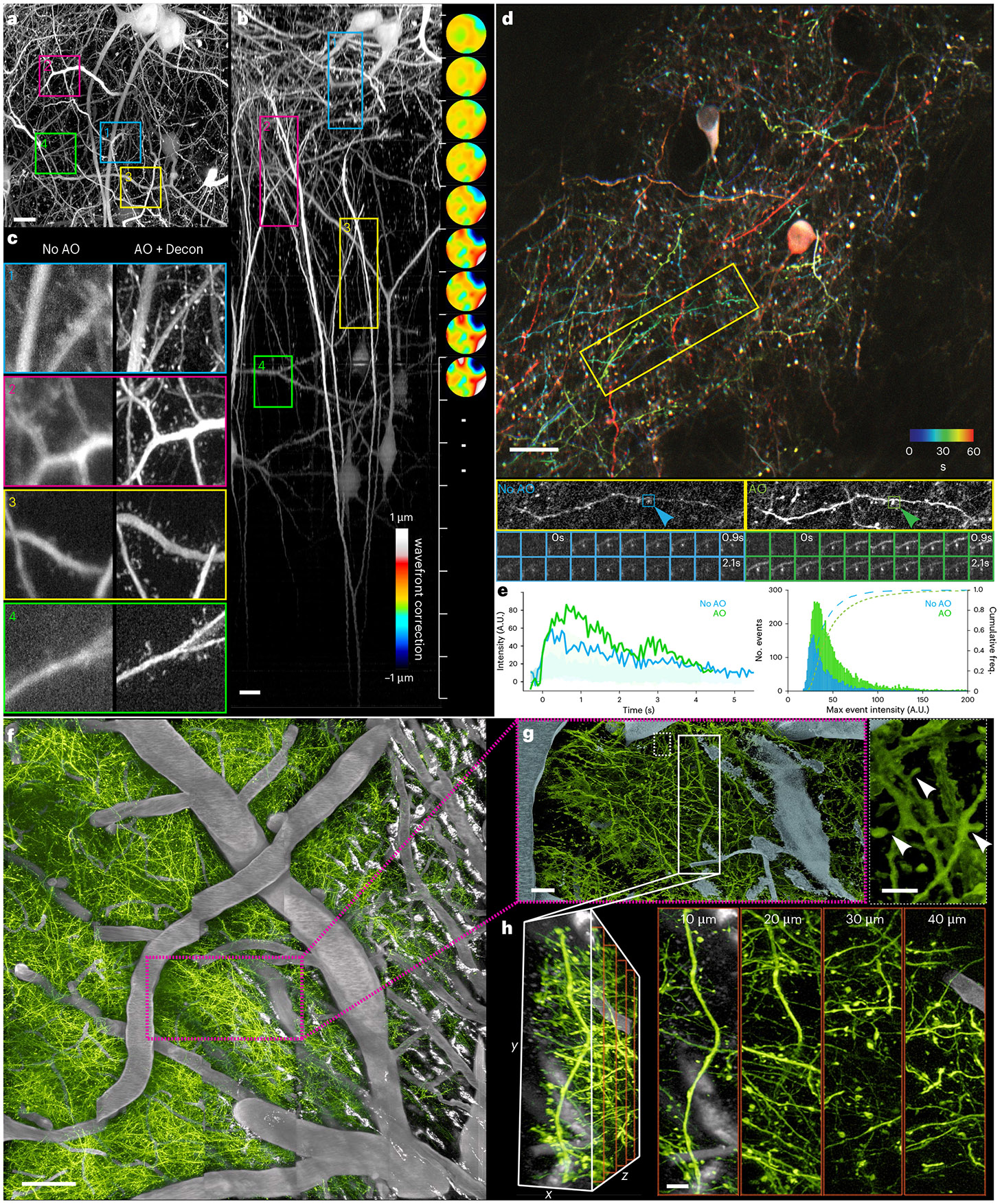
In vivo AO two-photon structural and functional imaging in the mouse cortex. **a**,**b**, *xy* (**a**) and *xz* (**b**) MIPs of a 100 × 100 × 400 μm^3^ volume starting at a depth of 60 μm in a Thy1-YFP-H mouse after AO correction and deconvolution (Supplementary Video 17). Wavefronts at right in **b** show the measured aberration at 25-μm intervals from 60 to 260 μm depth. Scale bar, 10 μm. **c**, Zoomed-in *xy* MIPs from four color-coded subvolumes as shown in **a** and **b**, before AO and after AO plus deconvolution. **d**, Functional imaging of neural activity 40 μm deep in the cortex of a GCaMP7s mouse (Supplementary Video 18). Top, color-coded time projection. Scale bar, 20 μm. Middle, single activated dendrite from the boxed region at top, before and after AO. Bottom, comparison of raw spikes from the indicated dendritic spine acquired sequentially without and with AO. **e**, Left, traces comparing measured calcium transients from the spines indicated above background in **d**. Background uncertainties are shown as 95% confidence intervals. Right, distribution showing that AO correction yielded ~2.5× more detectable dendritic spine calcium spikes than without AO over the same duration. **f**, Two-color in vivo AO-TPM of dendrites and vasculature, the latter labeled by injection of Texas Red, over a 500 × 500 × 100 μm^3^ FOV (Supplementary Video 19) with 5 × 5 × 1 AO corrective tiles. Scale bar, 50 μm. **g**, Left, zoomed-in view of the boxed region in **f**. Scale bar, 10 μm. Right, individual dendritic spines. Scale bar, 2 μm. **h**, Additional spines in a further magnified view of the boxed region in **g**. Scale bar, 5 μm.

The long excitation wavelength of two-photon microscopy (TPM) enables high-resolution fluorescence microscopy at depths in scattering medium inaccessible to the linear excitation methods presented above; however, as the same relationship of increasing sample-induced aberrations at greater depth still applies (for example, Fig. 6a,b), AO can be even more important for TPM.

MOSAIC offers two modes of AO-TPM. For acute brain slices, two-photon scanned Bessel beam light-sheet microscopy offers high speed and high axial resolution^36^; however, because both the excitation and detection axes are tilted with respect to the surface of the slice (Supplementary Fig. 1b), both wavefronts are increasingly sheared with increasing depth within the slice (Supplementary Fig. 23). AO is therefore needed to recover diffraction-limited performance (Supplementary Fig. 23).

For cortical imaging in live mice, MOSAIC includes an upright station for point-scanning TPM (Supplementary Figs. 1f and 24a,b). Here, AO is needed to compensate for aberrations introduced by the cortical window as well as the brain itself. Applying independent AO corrections every 25 μm to a depth of 400 μm (16 total corrections, Fig. 6b) over a 100 × 100 μm^2^ FOV (Fig. 6a), we recovered single-spine resolution throughout the volume (Fig. 6c and Supplementary Video 17) in a mouse expressing cytosolic Thy1-YFP-H in a sparse subset of neurons. In another mouse sparsely expressing GCaMP7s, we imaged neural activity 40 μm deep in dendrites at single-spine resolution at 16 frames per second for 125 s over a 200 × 200 μm^2^ FOV (Fig. 6d). AO correction improved the SNR of calcium transients (Fig. 6e and Supplementary Video 18), leading to the detection of approximately 2.5 times more calcium events (8,289 with AO versus 3,361 without) lasting 2 s or longer in dendritic spines during the same imaging duration.

The MOSAIC upright AO-TPM subsystem includes a 16-kHz resonant galvo and twin multi-pixel photon counting detectors for two-color imaging of fast events. For example, injecting Texas Red dye into the vasculature of a live Thy1-YFP-H expressing mouse allowed us to visualize the vascular network over a 500 × 500 × 100 μm^3^ volume in the context of the surrounding dendritic branches (Fig. 6f-h and Supplementary Video 19), and to follow the flow of individual erythrocytes in capillaries at 250 Hz over an 83 × 12.8 μm^2^ FOV (Supplementary Fig. 24d and Supplementary Video 20).

## Discussion

MOSAIC was conceived as an integrated platform to combine in a single instrument many of the most popular forms of optical microscopy developed during its 21st-century renaissance that reuses optical and electronic components across modalities, thereby (1) greatly decreasing capital and operating costs while increasing utilization rates compared to a collection of similarly performing single-purpose microscopes; (2) enabling correlative imaging of dynamic living systems thanks to fast in situ mode switching; and (3) extending the application scope of these methods to living multicellular systems, thanks to the incorporation of adaptive optics. As demonstrated by the results shown here, by these metrics it has succeeded.

That being said, MOSAIC is a complex instrument. Documentation alone comprises nearly 1,000 pages of slides and notes. Despite the shared components and prealigned optics, the instrument remains optomechanically complex, and simultaneous alignment of all modes (essential to rapidly and easily switch between them) requires considerable skill and patience. Even learning to operate an assembled MOSAIC across all modes requires a substantial investment of time and training. Fortunately, although an imaging core may require the full gamut of functionality to service diverse research projects, the modes are not interdependent. Thus, individual research groups can commission only a subset of them that are tailored to their specific applications.

To date, more than a dozen groups are using MOSAIC, which has already contributed to a number of technological studies^11,13,37-40^ and biological investigations^41-48^; however, these underscore yet another challenge: the high resolution and high imaging speed of which MOSAIC is capable, applied for long durations across the large specimens which MOSAIC is also equipped to handle, means data, and lots of it: up to 4 TB per hour and 30–100 TB per dataset. With PetaKit5D, the data acquired at this rate can be processed in real time on a high-performance distributed computing platform capable of ~15 trillion double-precision floating-point operations per second; however, the resulting post-processed 3D and 4D data at this scale are often too vast, too complex and too diverse for the human mind to comprehend. A multimodal machine-learning 4D foundation model offers a possible step forward to clearing this latest hurdle, and its development lies at the core of our current research^49^. MOSAIC plays a key role in generating the vast training data we need for this purpose. To address MOSAIC’s operational and data challenges at scale, we envision centralized facilities, modeled after astronomical observatories, which provide biologists with access to advanced microscopes, high-performance computing and expert staff. These cell observatories would host curated datasets and pretrained models and return both analysis-ready outputs and the raw data. Ultimately, these centralized cell observatories would serve as primary hubs for data generation and AI inference for deciphering the core principles of cell biology.

## Methods

### Microscope hardware

A complete MOSAIC CAD model, bill of materials, and nearly 1,000 pages of installation, maintenance and operation instructions are available through a no-cost research license agreement with Howard Hughes Medical Institute (HHMI).

#### Excitation sources.

Visible lasers 405 nm, 100 mW (TOPTICA, IBEAM-SMART-405-S-BZ), 445 nm, 100 mW (IBEAM-SMART-445-S-BZ), 488 nm, 500 mW (MPB, 2RU-VFL-P-500-488-B1R), 514 nm, 1,000 mW (MPB, 2RU-VFL-P-1000-514-B1R), 560 nm, 1,000 mW (MPB, 2RU-VFL-P-1000-560-B1R), 607 nm, 1,000 mW (MPB, 2RU-VFL-P-1000-607-B1R) and 642 nm, 2,000 mW (MPB, 2RU-VFL-P-2000-6224-B1R) were coaligned in a custom laser combiner and passed through an Acousto-Optic Tunable Filter (AOTF; AA Optoelectronic, AOTFnC-400.650-CPCh-TN) driven via a four-channel Multi-Purpose Digital Synthesizer (AA, MPDS4C-B66-22-52.111) which allowed rapid, independent control of their laser amplitudes. The output beam from the AOTF was then sent free-space to the downstream optics.

The femtosecond laser used for TPM (Coherent Chameleon LS II, tunable from 680 nm to 1,080 nm, peak power >3.5 W) was first corrected for group velocity dispersion (GVD) via a prism compressor (Thorlabs) before passing through an infrared Acousto-Optic Modulator System (AOM; AA, MT110-B50A1.5-IR-Hk driven via MPDS1C-B6-34-85.135) to control the output power delivered to the downstream optics.

#### Spatial light modulator.

A sample-conjugate SLM (Meadowlark P1920-0635-HDMI, 1,920 × 1,152 pixels) served multiple roles in MOSAIC: generation of LLS and 3D-SIM excitation patterns, five-axis alignment of the LLS to the detection focal plane, laser blanking, excitation AO correction, 3D phase modulation, light-sheet collimation and chromatic correction, and patterned photoactivation.

#### Mask.

A motorized custom glass wheel with a circumferential series of different photolithographically produced patterned metallic masks (Thorlabs) specific to different light sheets or SIM patterns was used to pass only the desired first-order diffracted light while blocking the unwanted zeroth and higher orders.

#### Dichroic stack.

To create a multicolor LLS, the collinear Gaussian beam from the laser combiner was transformed by a Powell lens/cylindrical lens combination (Supplementary Fig. 2a) into a collinear multicolor Gaussian light sheet, which was then spatially separated by a ‘dichroic stack’ (Supplementary Fig. 5a) into seven parallel light sheets that each impinge at separate locations on the SLM. The phase-only SLM imparted individually addressed phase delay patterns for each color and the resulting patterned LLSs reflected from the SLM back to the dichroic stack where the spatial offset was reversed, recombining into a collinear, multicolor set of up to seven overlapping LLSs that were ultimately relayed to the sample. The stack was a custom product (AVR Optics) constructed by sandwiching an ordered parallel set of laser dichroic filters (405, 442, 488, 532, 561 and 635 nm) and a mirror (to reflect any unfiltered light) using fused silica spacers (250 ± 50 μm) between layers.

#### Beam scanning.

A pupil-conjugate X-scan galvo (Cambridge Technology, 6SD11587) with a 60° optical range had two duties: it both selected the beam path (and therefore the objective) to illuminate, and translated the excitation (LLS, 3D phase, SIM, ISM or TPM) at the sample plane of the chosen objective. A pupil-conjugate *z*-scan galvo (Cambridge Technology, 6215H series, 6SD11226) moved the excitation orthogonal to *x*. A sample-conjugate 4-kHz resonant scanner (Cambridge Technology, CRS series, 6SC04KA040-01Y) wobbled the illumination (light sheet or 3D phase) to reduce shadowing artifacts from absorption or scattering within the specimen upstream of the viewed area, and a pupil-conjugate 4-kHz resonant scanner (Cambridge Technology, CRS series, 6SC08KA040-02Y) rapidly scanned a TPM-generated guide star (GS) in *x* across a desired FOV for measurement of sample-induced aberrations, or during TPM point scanning and TPM Bessel beam imaging.

#### Detectors.

For all imaging modes except TPM, two-color channels could be simultaneously recorded with a pair of sCMOS cameras (a pair of Hamamatsu ORCA-Fusion via CoaXPress or a pair of ORCA-Flash4.0 V3 via CameraLink). One camera also served as a Shack–Hartmann (SH) wavefront sensor for any of the four objectives when the path to this camera was appropriately switched to include a pupil-plane-conjugate microlens array (Edmund Optics, no. 64-479, focal length (FL) = 13.8 mm, pitch = 0.5 mm).

For TPM, fluorescence in two-color channels was measured simultaneously with two Multi-Pixel Photon Counters (MPPCs; Hamamatsu C13366-3050GA and C14455-3050GA). USB inspection cameras (Basler, puA2500-14u) were used during microscope alignment to monitor the excitation at pupil- and sample-conjugate planes before the excitation objective, while a triggerable USB camera (Basler, daA2500-14μm-CS) was used during autofocus to image the two-photon GS through the excitation objective when the GS was generated through the detection objective. Finally, a waterproof USB endoscope (Depstech USB-C & Micro USB Endoscope) was used to broadly image the inside of the sample chamber to assist with positioning the sample between the objectives.

#### Specimen positioning and scanning.

At the primary (three-objective) imaging station, the sample chamber assembly (including the bath, sample stages and the sample chamber) was raised or lowered using a 1-inch motorized stage (Thorlabs, ZFM2030) and then manually slid on rails out and away from the objectives toward the operator for safe and easy sample exchange. The sample was translated to the desired FOV by three orthogonal piezo stick-slip stages (SmarAct, SLC-2445-S, CLS-5252-S and CLS-5252-S), which formed the *xyz* stage assembly. For experiments requiring long-range, long-duration, continuous (as opposed to step-and-settle) scanning on the centimeter scale (for example, ExLLSM), an electromagnetic direct-drive stage (SmarAct, MLS-3252-S) could replace the *x* stick-slip stage. The lack of friction in the direct drive allowed highly consistent sweep performance, which was needed for experiments that would benefit from thousands of identically repeated sweeps. At the upright station, the objective was positioned in *z* with a brushless linear servo objective scanner (Dover, DOF-5). The specimen was mounted on a manual *z* stage (Thorlabs, VAP4) and a motorized *xy* stage (Thorlabs, PLS-XY), with customization available as needed.

#### Sample chamber.

The sample chamber for the primary imaging station was environmentally sealed to prevent evaporation and allowed for closed-loop (TE Technology, TC-720) temperature control from ambient to 37 °C via silicone heaters (Tempco and Birk Manufacturing) and featured both peristaltic perfusion (Warner Instruments, 890688) and control of CO_2_ concentration.

#### Frame.

The mainframes of the microscope were machined from 1-inch aluminum (ALCOA MIC-6 tooling plate). Placement of holes for screws, dowel pins and locator pins was with ±0.0005-inch tolerances.

### Optical paths and shared components

The exact optical paths for each mode are illustrated in Supplementary Figs. 2-7 and Supplementary Video 1, and described as follows:

#### Lattice light-sheet microscopy path.

Coaligned Gaussian beams emerged from the laser combiner/AOTF and passed through a Powell lens (Supplementary Fig. 2a; 20° fan angle, Laserline Optics, LOCP-8.9R20-2.0) that expanded the beam in the *x* direction, followed by a conventional achromatic lens (FL = 50 mm) that collimated the beam in *x* and focuses it in *z*, creating a narrow line of illumination. A pair of cylindrical lenses (FL = 50 mm and FL = 300 mm) then relayed and demagnified this line of illumination, creating (through the dichroic stack) seven parallel lines of different wavelengths at the SLM. The patterned light sheets reflected from the sample-conjugate SLM were first recombined by the dichroic stack and then transformed by a lens (FL = 400 mm) en route to the pupil-conjugate mask wheel. Another transform lens (FL = 150 mm) relayed the transmitted light to the sample-conjugate resonant galvo used to wobble the light sheet angle within the specimen. Another transform lens (FL = 80 mm) relayed the light to the sample-conjugate *z* galvo before a 4f pair of lenses (both FL = 150 mm) relayed further to the *x* galvo. A final transform lens (FL = 400 mm) relayed the light to the pupil of the excitation objective (Thorlabs, ×20, 0.6 NA), which then formed the LLS within the specimen.

Fluorescence generated by the LLS within the specimen was captured by the detection objective (Supplementary Fig. 2b; Zeiss 421452-9800-000, ×20, 1.0 NA) and then relayed by a tube lens (Zeiss 423731-8246-000, 165-mm FL) and a 50-mm effective focal length (EFL) lens doublet to a pupil-conjugate DM (ALPAO DM-69). A dichroic mirror (Semrock, DI03-R561-T3-32 × 40) split the fluorescence by color, and a 300-mm FL lens in each path created an image on an sCMOS camera for simultaneous two-color imaging.

#### Line-scan image scanning microscopy path.

In our implementation of line-scan ISM, the multicolor Gaussian beam from the laser combiner was converted by a Powell lens (Supplementary Fig. 3a; Laserline Optics LOCP-8.9R20-2.0) and a conventional achromatic lens (75-mm FL) into a focused line of illumination at a sample-conjugate slit mask (Thorlabs, MPH-16). A 100-mm FL lens transformed the excitation to a pupil-conjugate plane, which was then relayed to the pupil-conjugate *z* galvo by a 25-mm EFL lens doublet/40-mm EFL lens doublet pair. Two 75-mm EFL lens doublets then formed a 4f system to relay to the *x* galvo. A 75-mm EFL doublet pair and a 350-mm FL lens relayed from the *x* galvo to a DM for AO correction (ALPAO, DM-69). The excitation was then relayed to the pupil of any of the detection, upright (Zeiss 421452-9880-000) or inverted (Zeiss) objectives (depending on the nature of the specimen), in the first case with a 50-mm EFL doublet/165-mm FL tube lens (Zeiss 423731-8246-000) relay pair, and in the other two cases with an additional pair of 62.5-mm EFL lens doublets inserted in between the 50-mm EFL and 200-mm FL lenses. For all three objectives, the resulting line illumination was ~1 Airy unit (AU) wide at the specimen.

The fluorescence generated by the line illumination retraced the optical path back through the microscope, until passing through a multiband dichroic mirror (Semrock, Di01-R405/488/561/635/800-1050-t3-25 × 36) just after the 40-mm EFL lens doublet. In sequence, 100-mm, 90-mm and 50-mm FL lenses then transferred the fluorescence to the dichroic that split and directed it to the two cameras. Because it was retraced across the *x* and *z* galvos, the ISM line fluorescence was descanned and hence stationary with respect to the imaging cameras, where it was aligned with the fast-readout direction of each camera to maximize readout speed.

To generate an ISM image, the line excitation was scanned in *x* across the desired FOV, and the fluorescence in the rectangular region defined by the FOV in *y* and the 1-AU-wide stripe in *x* was recorded as an image. This image was then reconstructed as a single-pixel column of extended resolution in *x* by the ISM algorithm^50^.

#### Widefield structured illumination microscopy path.

The multicolor Gaussian beam from the laser combiner was magnified by a 25-mm FL/300-mm FL lens pair to fully illuminate the entire SLM (Supplementary Fig. 2d). A grating pattern in one of three different orientations applied to the SLM created a diffraction pattern consisting of three coplanar beams. After a 400-mm FL transform lens, these three beams became collinear and passed through the pupil-conjugate mask, while other diffraction orders were blocked. Two Zaber rotators (RSB060AD-E01T3-MC03) enforced linear polarization of the beams orthogonal to their common plane, as previously described^51^. A 150-mm FL lens then focused the beams to create an image of the desired SIM grating pattern at the sample-conjugate 4-kHz resonant wobble galvo, which rapidly modulated the angle at which the beams converged within the specimen to reduce striping artifacts from absorption or scattering. An 80-mm EFL lens doublet transformed this pattern to the pupil-conjugate *z* galvo, and a 4f lens pair consisting of two 150-mm EFL doublets relayed this to the *x* galvo. Together these galvos translated the grating pattern within the specimen and provided the phase stepping necessary for 3D-SIM. A 300/350-mm FL lens pair then relayed the excitation to the pupil-conjugate DM for AO correction. From there, it was transmitted through a 100-mm FL lens doublet to the pupil plane of any one of the three objectives: to the detection objective via the 165-mm FL Zeiss tube lens, and to either the inverted or upright objective via a 125-mm FL/125-mm FL relay pair plus the tube lens.

Fluorescence generated in the specimen retraced the excitation path until passing the multiband dichroic mirror, after which it followed the widefield detection path (Supplementary Fig. 2b) where it was split into two-color channels and transformed by parallel 300-mm FL lenses for simultaneous two-color detection on the cameras.

#### Oblique illumination path.

Illumination at the SLM was either full-chip (same optics as widefield SIM; Supplementary Fig. 2d) or line-like (same optics as LLSM; Supplementary Fig. 2a). Thereafter, the oblique excitation followed the same path (Supplementary Fig. 2c) to the excitation objective as in LLSM. Full chip yielded point illumination at the excitation pupil, while line-like yielded line illumination at the pupil along the *z* axis perpendicular to the plane of the light sheet it produces at the sample. In either case, applying a phase ramp to the SLM changed the position in *z* of the illumination at the pupil plane, and thus the angle of the illumination at the sample. In this manner, the OI could be varied anywhere from dark field to differential interference contrast-like to bright field (C4 to C1; Supplementary Fig. 10) with respect to the inverted objective. Laser speckle due to the coherent illumination was reduced by rapidly dithering the illumination in *x* and *z* at the specimen with the galvos, while keeping the position of the illumination in the pupil (and thus its angle at the sample) fixed.

From the inverted objective, the collected light progressed through the 165-mm FL tube lens, a pair of 125-mm EFL doublets and a 100-mm EFL doublet to the DM, and a 300-mm FL transform lens from there to an sCMOS camera (Supplementary Fig. 2c).

#### Point-scanning two-photon microscopy path.

Femtosecond pulses from the laser/GVD compensator (Prism Compression kit, Thorlabs AO-LLS-002)/AOM subsystem (Supplementary Fig. 24e) were beam-expanded by two relay lens pairs (25-mm FL/40-mm FL and 25-mm FL/60-mm FL) to the pupil-conjugate 8-kHz resonant scanner for rapid point-scan imaging. From there, a 100-mm FL/160-mm FL lens pair relayed the excitation to the *z* galvo. The remainder of the excitation path to the focus of the upright objective was the same as for ISM (Supplementary Fig. 3a).

Fluorescence generated in the specimen was collected by the upright objective and separated from the excitation with a dichroic mirror (Supplementary Fig. 4a; Semrock, FF757-DI01-32 × 40). A 150-mm FL lens with its front focal plane at the rear pupil of the upright objective served as the first lens of a relay pair, followed by a dichroic mirror (Semrock, Di03-R561-t1-25 × 36) to split the emission into two-color channels. The 25-mm FL lenses completed the relay pair in each color path, resulting in the fluorescence at the rear pupil being imaged onto a pair of MPPC detectors for simultaneous two-color imaging.

A multi-function, high-speed digitizer (Thorlabs) measured the MPPC voltages and produced a 2D image that was returned to the acquisition software.

#### Upright two-photon Bessel beam microscopy path.

MOSAIC included the option of using a two-photon Bessel beam with extended depth of focus at the upright station, resulting in an image at each *z* scan plane which was effectively a MIP in *z* of the signal over the extended focal range^52^.

For this mode, after the femtosecond beam emerges from the AOM, it was expanded by a 25-mm FL/150-mm FL lens pair and delivered to the apex of an axicon (Thorlabs AX252-B, 2° cone angle; Supplementary Fig. 4c). The resulting diverging ring of illumination was converted to a collimated ring by one of three different lenses (125-mm, 175-mm or 250-mm FL), resulting in one of three different ring diameters, and thus ultimately one of three different combinations of 50-, 100- or 200-μm Bessel beam lengths. A pair of 100-mm FL lenses relayed the ring excitation to a pupil-conjugate annular mask, where each diameter had its own circular ring in the mask to reject zeroth-order (DC) and other extraneous light. A 150-mm FL/100-mm FL lens pair relayed the beam transmitted through the mask to the same pupil-conjugate 8-kHz resonant scanner used for point-scanning TPM. Thereafter, the rest of the excitation path, and all of the detection path, was identical to that for point-scanning TPM.

In practice, femtosecond power losses through MOSAIC and the loss of energy in the Bessel beam side lobes rendered this mode undesirable compared to the simpler point-scanning TPM.

#### Two-photon scanned Bessel beam light-sheet illumination path.

The optical path in this case (Supplementary Fig. 3c) was initially identical to that of the upright Bessel path up to the rotating mask, which again had three transmissive annuli for Bessel beams of different NAs and lengths, but of different diameters to accommodate the different pupil diameters of the light-sheet excitation objective compared with the upright one. Thereafter, the light path to the excitation objective was identical to that in the LLS mode (Supplementary Fig. 2a). Likewise, the detection path was identical to widefield detection (Supplementary Fig. 2b).

#### Adaptive optical two-photon guide star path.

Femtosecond pulses from the laser/GVD compensator/AOM subsystem were beam-expanded by two relay lens pairs (25-mm FL/40-mm FL and 25-mm FL/60 mm FL) to the pupil-conjugate 8-kHz resonant scanner (Supplementary Fig. 3b), which was left stationary during wavefront measurement. From there, a 25-mm EFL/40-mm EFL doublet lens pair relayed the excitation to the *z* galvo, and a 75-mm EFL/75-mm EFL doublet lens pair relayed it to the *x* galvo. This galvo then switched the remaining path depending on which objective the aberration was being measured for. A 75-mm EFL doublet/40-mm FL lens pair was used to relay from the *x* galvo to the pupil plane of the excitation objective. The path to the other three objectives started with a 75-mm EFL doublet/350-mm FL relay pair to the DM. From there, a 50-mm EFL doublet / 165-mm FL tube lens pair relayed the excitation to the pupil of the detection objective, whereas for the inverted or upright objective, a 62.5-mm EFL/62.5-mm EFL doublet pair relay was added between the 50-mm EFL/200-mm FL elements to extend the excitation to their rear pupils.

For all four objectives, the fluorescent GS generated in the specimen retraced that objective’s excitation path until it reaches the dichroic mirror (Semrock, DI01-R405_488_561_635_800-1050-T3_25 × 36) between the 25-mm EFL and 40-mm EFL doublets used to relay from the 8-kHz resonant scanner to the *z* galvo. This ensured that the fluorescence was stationary (‘descanned’) before it reached the pupil-conjugate microlens array (Edmund, 64-479) used for SH wavefront measurement. Finally, the array of foci was relayed to a camera (Hamamatsu ORCA Flash or Fusion), where deviations in their positions from their initial system-corrected locations encoded the specimen-induced aberration for the objective under investigation.

#### Patterned photostimulation path.

Patterned photostimulation could be achieved via the SIM excitation pathway by applying the desired stimulation pattern as a DC value on the SLM, with a high-frequency grating pattern elsewhere to deflect light outside the aperture of the mask or the objective rear pupil.

Point scanning of either the linear or TPM excitation was also an option for photostimulation when higher peak intensity was needed. In the TPM case, the excitation followed the TPM GS path with the 8-kHz resonant scanner disabled and held fixed. The *x* and *z* galvos were used to raster the spot over the desired photostimulation pattern. Alternatively, photostimulation excitation could originate from the laser combiner. The beam was first expanded by a lens pair (FL = 25 mm and FL = 60 mm) before the 8-kHz resonant scanner, after which it joined and followed the TPM path just described. Supplementary Fig. 26 provides a decision tree to guide MOSAIC modality selection.

### Adaptive optics

#### Correction of system aberrations in the detection path (phase retrieval).

Using widefield illumination, the 3D PSF of an isolated, subdiffractive fluorescent bead was recorded using the objective lens of interest. A phase-retrieval algorithm^53^ then calculated the wavefront shape. This was decomposed into Zernike polynomials, which were then applied to the pupil-conjugate DM using the vendor-supplied mode-by-mode calibration. The process was then repeated until the residual aberration was less than λ/4 peak-to-peak (essentially diffraction-limited). Recalibration was necessary whenever the detection path was realigned, optics were replaced, or a different imaging buffer was used.

#### Correction of system aberrations in the excitation path (pupil segmentation).

By definition, system aberrations in the excitation path from the sample-conjugate SLM to the sample-conjugate focal plane of the excitation objective are null when the wavevectors from all points in the objective rear pupil constructively interfere at the excitation focus. The pupil-conjugate wavefront correction needed to achieve this was determined by pupil segmentation^54^. In brief, the pupil wavefront was computationally represented as a large grid of small patches, each having its own independent yet constant phase. Treating one patch as a fixed reference, the appropriate corrective phase for each of the others was determined sequentially by calculating and applying to the SLM the grating pattern that would appeared if light from the two patches coherently interfered there. When the SLM was illuminated, this pattern created three beams at the excitation pupil – one each at the current and reference patches, and one between them. This beam was blocked by the rotating mask, so a standing wave of illumination was created at the excitation focal plane, where a fixed subdiffractive fluorescent bead has been placed. The phase of the current patch needed to maximize bead brightness determined its corrective phase relative to the reference patch. The process was repeated for all other patches, at which point the complete excitation-pupil phase required for system aberration correction was known. During imaging, this corrective phase was added to that required for sample-induced aberration to achieve complete excitation correction.

#### Correction of sample-induced aberrations in the detection path.

The procedure used here was as previously described^9^. Focused two-photon excitation created a fluorescent GS at a desired imaging plane within the specimen for one of the three 1.0-NA objectives (detection, upright or inverted). *x* and *z* galvos swept the GS across an assumed isoplanatic patch (region of near-constant aberration), and the collected descanned light was sent to the SH camera for wavefront measurement, discarding those spots that did not contain at least 1,000 photons or whose localization precision exceeded 0.5 pixels. The displacements of the spots from their locations after correction of system aberration (see above) encoded the gradient of the sample-induced aberration. Wavefront reconstruction^55^ then recovered the wavefront itself, which was then decomposed into its Zernike coefficients. The inverse of these was then applied to the DM to provide the necessary AO correction. As the two-photon focus was itself aberrated at the start of correction, limited SNR and imperfect calibration often led to only partial correction after one iteration. The process was thus iterated in a closed loop until diffraction-limited performance was achieved. Starting de novo in a new specimen, typically five iterations were needed; however, when moving from one isoplanatic patch to an adjacent one, or when updating an existing correction at a later time point, one to two iterations were usually sufficient.

More recently, rather than stepping from patch to patch to perform correction, the specimen was translated continuously while multiple SH measurements were performed at a desired density. After the AO corrections were calculated from these measurements, the sample was thereafter continuously translated during imaging with continuous corrective updates applied to the DM.

#### Correction of sample-induced aberrations in the excitation path.

Measurement of the sample-induced aberration in the excitation path proceeded with a two-photon-induced GS and SH sensor exactly as above; however, because the SLM that created and corrected the excitation pattern was sample-conjugate, AO correction was achieved by subtracting the system- and sample-induced aberrations *φ*_system_ and *φ*_sample_ from the complex phase of the Fourier transform of the ideal SLM pattern, transforming back to the sample plane, and applying the real part as the new corrected SLM pattern:

$${\text{SLM}}_{\text{corrected}}=\text{Re}\{{\text{FT}}^{-1}[\text{FT}\;(SLM_{\text{ideal}})\;\text{exp}(-i(\varphi_{\text{system}}+\varphi_{\text{sample}}))]\}$$

Because this correction did not affect subsequent GS measurements of the residual aberration, the process was open-loop and only one iteration could be performed.

#### Continuous autofocus via relative LLSM and guide star axial position measurement.

During LLSM imaging, sample-induced refraction of the light sheet and/or sample-induced defocus of the detection objective focal plane could occur, causing these two entities to lose their precise overlap necessary for high-resolution imaging. To compensate, the two-photon GS used for AO correction in the detection path could be simultaneously viewed through the excitation objective with a sample-conjugate camera, along with a side-on view of the light sheet (Supplementary Fig. 25). After correction for the static focal offset of the TPM excitation, the *z* galvo could be adjusted continuously during imaging to maintain the proper axial alignment of the light sheet and detection focal planes^9^.

### Microscope control software

Custom microscope control software was developed using National Instruments LabVIEW 2018 SP1-2022 (64 bit) to manage all MOSAIC operations. The software provided a graphical user interface that enabled users to switch among MOSAIC modalities, control the hardware, acquire data and calibrate the system (for example, autofocus and system aberration correction). The software supported multi-position acquisition and user scripting, allowing sequential multimodal imaging across multiple specimens in a single experiment. Switching between modalities was achieved by opening and closing the motorized flip mirror (Supplementary Fig. 5c) to direct light to different paths. Multiple specimens could be loaded together in the same experiment, provided that they could fit on one 25 × 25-mm coverslip and required the same imaging environment (such as medium, temperature and CO_2_).

Real-time data compression was offloaded to a PCIe accelerator (Intel QuickAssist Adapter 8970) when saving data files. Raw data could be saved either in Tiff or Zarr format. A GPU (NVIDIA, GTX Titan or RTX A6000) drove MOSAIC control workstation monitors and delivered patterns to the SLM. Digital and analog voltage control input and output signals were handled using an FPGA (NI, USB-7845R OEM).

### Sample preparation

#### Standard cell culture conditions.

Unless otherwise specified, mammalian cell lines were cultured at 37 °C in a humidified atmosphere containing 5% CO_2_. Cells were regularly tested for mycoplasma contamination. For live imaging, cells were typically plated on no. 1.5 thickness glass coverslips (Thorlabs, CG15XH; Bellco, 2291-74000; Warner Instruments, CS-25R15).

#### U2OS cells.

In Fig. 1d, for OI imaging, unlabeled U2OS cells were cultured in Dulbecco’s modified Eagle’s medium (DMEM) with GlutaMAX (Gibco, 10566016) supplemented with 10% fetal bovine serum (FBS; Seradigm) at 37 °C, 5% CO_2_ and 100% humidity. U2OS cells were grown on coverslips. When cultures reached 50–60% confluency, cells were transferred to an imaging chamber containing Leibovitz’s L-15 Medium without phenol red (Gibco, 21-083-027) supplemented with 5% FBS (ATCC SCRR-30-2020).

In Fig. 3a, for imaging mitochondria and nuclear lamins via DNA-PAINT, U2OS cells with an integrated TOMM20–HaloTag fusion protein were used^56^. U2OS cells were plated 1 day before imaging on no. 1.5 thickness, 15-mm-diameter coverslips containing nanodiamond fiducials as described earlier^57^ at a cell density of 1 × 10^5^ cells per ml. Cells were fixed 24 h after plating using 4% PFA in 1× phosphate-buffered saline (PBS) for 20 min. After fixation, cells were washed with 1× PBS three times and then incubated in fresh 1× PBS for 5 min. Cells were then permeabilized in 0.2% Triton X-100 in 1× PBS for 20 min. After permeabilization, cells were washed with 1× PBS and then blocked with 10% normal goat serum (Thermo) overnight at 4 °C. After blocking, cells were labeled with mouse primary antibody against Lamin A/C (Santa Cruz, sc-376248) at a concentration of 1:200 and a DNA-conjugated anti-GFP nanobody (Massive Photonics, MASSIVE-TAG-X2 anti-GFP) at a concentration of 1:200 to label the TOMM20–HaloTag fusion protein in 10% normal goat serum overnight. After primary labeling, the cells were washed with Massive Photonics washing buffer for 1 h and then labeled with DNA-conjugated secondary antibodies against the mouse anti-Lamin A/C primary antibody (MASSIVE-sdAB 1-PLEX) at a concentration of 1:200 for 1 h in washing buffer. After secondary labeling, cells were washed in 1× washing buffer three times for 5 min. For imaging, the complementary imaging strands were diluted in Massive Photonics imaging buffer at a concentration of 0.75 nM (imager 2, complementary to anti-GFP) and 0.2 nM (imager 1, complementary to anti-mouse secondary).

#### HeLa cells.

For Fig. 1c, wild-type HeLa cells were cultured in DMEM with GlutaMAX (Gibco, 10566016) supplemented with 10% FBS (Seradigm) at 37 °C, 5% CO_2_ and 100% humidity. When HeLa cells grown on coverslips (Thorlabs, CG15XH) reached 30–50% confluency, they were transferred to an imaging chamber containing Leibovitz’s L-15 Medium without phenol red (Gibco, 21-083-027) supplemented with 5% FBS (ATCC SCRR-30-2020).

#### LLC-PK1 cells.

For Fig. 1a,b, pig kidney epithelial cells (LLC-PK1, a gift from M. Davidson at Florida State University) were cultured in DMEM with GlutaMAX (Gibco, 10566016) supplemented with 10% FBS (Seradigm) at 37 °C, 5% CO_2_ and 100% humidity. LLC-PK1 cells stably expressing the ER marker mEmerald–Calnexin and the chromosome marker mCherry–H2B were grown on coverslips (Thorlabs, CG15XH) coated with 200-nm fluorescent beads (Invitrogen FluoSpheres Carboxylate-Modified Microspheres, 505/515 nm, F8811). When cultures reached 30–80% confluency, cells were transferred to an imaging chamber containing Leibovitz’s L-15 Medium without phenol red (Gibco, 21-083-027) supplemented with 5% FBS (ATCC SCRR-30-2020) and an antibiotic cocktail (0.1% ampicillin, 0.1% kanamycin and 0.1% penicillin–streptomycin; Thermo Fisher).

#### hTERT-RPE1 cells.

For Fig. 2b-f and Supplementary Figs. 11, 12 and 14-17, hTERT-RPE1 cell lines were generated as described previously^58^. In brief, to produce the ER-StayGold/Golgi-HaloTag-tagged and Mitochondria-StayGold/Golgi-HaloTag-tagged hTERT-RPE1 cell lines, we transduced the parental ER-StayGold and Mitochondria-StayGold cells with lentiviral particles encoding HaloTag-tagged β4Gal (a Golgi-resident enzyme), respectively. Approximately 300 μl of β4Gal-HaloTag9 lentivirus was added to cells seeded in six-well plates; 2 days post-infection, the top 5% of HaloTag fluorescence (detected in APC-A) was sorted using a BD FACSAria Fusion Sorter and BD FACSDiva Software. For sorting, cells were incubated at 37 °C for 15 min in DMEM/F12 (Thermo Fisher Scientific, 11320033) containing Janelia Fluor HaloTag Ligand 646 (diluted 1:20,000 from a 1 mM stock), washed three times with PBS, then trypsinized and expanded for imaging. Lentiviral vectors were constructed by amplifying the β4Gal-HaloTag9 sequence from pcDNA5/FRT/TO_b4g-HaloTag9 (Addgene #175546), each cloned into a lentiviral vector with an EF1α promoter (a derivative of Addgene #60955 with the sgRNA sequence removed). Lentiviral particles were produced by transfecting HEK293T cells (ATCC CRL-3216) with these plasmids and standard packaging vectors using TransIT-LT1 Transfection Reagent (Mirus, MIR2306); the medium was replaced 24 h post-transfection and the viral supernatant was collected approximately 50 h post-transfection and filtered through a 0.45-μm PVDF syringe filter. Cells were cultured in DMEM/F12 supplemented with 10% FBS (VWR Life Science 100% Mexico Origin 156B19), 10 μg ml^−1^ hygromycin (Invitrogen, 10687010), 2 mM L-glutamine, 100 U ml^−1^ penicillin and 100 μg ml^−1^ streptomycin (Fisher Scientific, 10378016), and passaged using 0.25% trypsin–EDTA with phenol red (Fisher Scientific, 25200114). For the Supplementary Fig. 11 experiment, cells were transfected approximately 24 h before imaging with a total of 1 μg DNA, consisting of 0.3 μg of pCSII-EF/mt-(n1)StayGold (Addgene plasmid #185823)^59^ plasmid and 0.7 μg of carrier DNA, using Lipofectamine 3000 (Thermo Fisher Scientific). Upon reaching 30–80% confluency, cells were incubated with the cell-permeable fluorescent ligand JFX549 to label the Golgi apparatus for 15 min, then washed with Leibovitz’s L-15 Medium without phenol red (Gibco, 21-083-027). The coverslips were subsequently transferred to L-15 Medium at 37 °C, supplemented with 5% FBS (ATCC SCRR-30-2020) and antimicrobial reagent (Primocin, 100 μg ml^−1^, InvivoGen), for imaging.

For Supplementary Fig. 13, for the photoactivation experiments, hTERT-RPE1 cells were cultured in DMEM supplemented with 10% FBS (Avantor Seradigm) and 10 μg ml^−1^ hygromycin. Cells were transfected approximately 24 h before imaging with a total of 1 μg DNA, consisting of 0.3 μg of plasmid of interest and 0.7 μg of carrier DNA, using Lipofectamine 3000 (Thermo Fisher Scientific). The plasmids used were pTriEx–mCherry–PA-Rac1 (Addgene plasmid #22027)^19^. Imaging of hTERT-RPE1 cells was performed at 37 °C at a confluency of 30–50%, using Leibovitz’s L-15 Medium without phenol red (Gibco, 21083027) supplemented with 10% FBS, 0.1% ampicillin, 0.1% kanamycin and 0.1% penicillin–streptomycin.

#### COS-7 cells.

African green monkey kidney fibroblast-like cell line (COS-7, ATCC, CRL-1651) was cultured in DMEM supplemented with GlutaMAX (Gibco, 10566016) and 10% FBS, in a 5% CO_2_ incubator at 37 °C. COS-7 cells transiently expressing EGFP–Tub1A and the actin marker mCherry–LifeAct were plated onto 25-mm round coverslips (Thorlabs, CG15XH). When cultures reached 30–80% confluency, cells were transferred to an imaging chamber containing Leibovitz’s L-15 Medium without phenol red (Gibco, 21-083-027) supplemented with 10% FBS (Life Technologies).

#### mES cells.

For Fig. 2a, mouse embryonic stem (mES) cells expressing HaloTag-Sox2 were a gift from J. Liu at the Janelia Research Campus^60^. The mES cells were cultured in KnockOut DMEM (Thermo Fisher Scientific, 10829018) supplemented with 10% FBS, ES Cell Qualified (ATCC, SCRR-30-2020), 1× GlutaMAX Supplement (Thermo Fisher Scientific, 35050061), 1× MEM Non-Essential Amino Acids Solution (Thermo Fisher Scientific, 11140050), 0.1 mM 2-mercaptoethanol (Sigma-Aldrich, 444203), 1× Antibiotic-Antimycotic (Thermo Fisher Scientific, 15240062) and 1,000 U ml^−1^ of Leukemia Inhibitory Factor (StemCell Technologies 78055), 1 μM PD0325901 (Sigma-Aldrich PZ0162) and 3 μM CHIR99021 (Sigma-Aldrich SML1046). Two days before imaging, 25-mm no. 2 coverslips were precoated with Biolaminin 511 (Biolamina, LN511) according to the manufacturer’s protocol. Recombinant laminins were thawed slowly at 4 °C and diluted in 1× DPBS containing Ca^2+^ and Mg^2+^ (Sigma-Aldrich 59300C) to a final coating concentration of 0.5 μg cm^−2^. The diluted solution was applied to coverslips and incubated overnight at 4 °C. One day before imaging, 0.5 × 10^6^ cells were plated onto coated coverslips. Immediately before imaging, cells were incubated in fresh growth medium supplemented with 10 nM HaloTag ligand PA-JF647 and a 1:500 dilution of SPY 505 (cytoskeleton CY-SC101) for 1 h. Cells were washed once with growth medium and incubated for an additional 20 min in growth medium containing no dye.

#### Mouse brain slice.

For Supplementary Fig. 23, 300-μm thick acute brain slices were prepared from 3-month-old Thy1 GFP-M mice using the *N*-methyl-D-glucamine (NMDG) protective recovery method^61^. Mice were anesthetized with ketamine/xylazine (200 mg kg^−1^ ketamine and 20 mg kg^−1^ xylazine) via intraperitoneal injection and perfused with oxygen-bubbled, ice-chilled NMDG–HEPES artificial cerebrospinal fluid (aCSF) (92 mM NMDG, 2.5 mM KCl, 1.25 mM NaH_2_PO_4_, 30 mM NaHCO_3_, 20 mM HEPES, 25 mM glucose, 2 mM thiourea, 5 mM Na-ascorbate, 3 mM Na-pyruvate, 0.5 mM CaCl_2_·2H_2_O and 10 mM MgSO_4_·7H_2_O, titrated to pH 7.3). The brain samples were dissected and cut into 300-μm-thick slices using a Leica VT1200S vibratome under oxygen-bubbled, ice-chilled NMDG–HEPES aCSF. The slices were then transferred into a 34 °C, oxygen-bubbled NMDG–HEPES aCSF recovery chamber. Following Na^+^ spike-in, the slices were transferred to a slice holding chamber with oxygen-bubbled HEPES aCSF (92 mM NaCl, 2.5 mM KCl, 1.25 mM NaH_2_PO_4_, 30 mM NaHCO_3_, 20 mM HEPES, 25 mM glucose, 2 mM thiourea, 5 mM Na-ascorbate, 3 mM Na-pyruvate, 2 mM CaCl_2_·2H_2_O, and 2 mM MgSO_4_·7H_2_O, titrated to pH 7.3). The slices were then placed onto a poly-D-lysine-coated no. 1.5 coverslip with a slice anchor (Warner Instruments) for imaging.

#### 4×-expanded hippocampal tissue from patients with Alzheimer’s disease.

For Fig. 3b-f, postmortem human hippocampal specimens were obtained from the University of Washington BioRepository and Integrated Neuropathology (BRaIN) laboratory and the University of Washington Alzheimer’s Disease Research Center Precision Neuropathology Core. In brief, a fresh piece of posterior hippocampus was dissected at rapid autopsy (postmortem interval <12 h) and immediately fixed in 4% (w/v) paraformaldehyde (PFA) in 1× PBS at room temperature for 48 h. The fixed tissue block was sectioned to ~100-μm-thick slices using a vibratome (VT1200S, Leica) and stored in 1× PBS with 0.03% (w/v) sodium azide at 4 °C.

The brain slices were permeabilized with 0.1% (w/v) Triton X-100 in 1× PBS for 15 min and incubated in blocking buffer (5% (v/v) normal goat serum and 0.1% (w/v) Triton X-100 in 1× PBS) at room temperature for at least 6 h before immunostaining. The ~100-μm-thick brain slices were immunostained with primary antibodies of rabbit anti-NF-200 (1:300 dilution, N4142-.2ML, Millipore Sigma) and chicken anti-MBP (1:300 dilution, PA1-10008, Thermo Fisher) and secondary antibodies goat Atto 647N-conjugated anti-rabbit (1:200 dilution, 40839-1ML-F, Millipore Sigma) and goat Alexa Fluor 568-conjugated anti-chicken (1:200 dilution, A11011, Thermo Fisher Scientific). After staining, samples were prepared following the protein-retention expansion microscopy protocol with minor modifications^62,63^.

Postmortem human specimens were collected with informed consent from the patients in consultation and compliance with the University of Washington School of Medicine Compliance Office and Health Insurance Portability and Accountability Act.

#### Zebrafish.

All zebrafish experiments were performed in accordance with standard protocols and approved by the Institutional Animal Care and Use Committees of Stony Brook University, the Janelia Research Campus and the University of California, Berkeley. Specific UC Berkeley Animal Use Protocols included AUP-2019-09-12560-1 (Upadhyayula Laboratory), AUP-2020-10-13737-1 (Swinburne Laboratory), and AUP-2021-05-14347-1 (Zebrafish Facility Core Protocol). All zebrafish used were embryos younger than 7 dpf, and sex was not a factor in these studies.

The zebrafish lines used in this study were (*Tg(eef1a1l1:mem-2x-mchilada)^hm801^*) (Fig. 5a-e and Supplementary Figs. 19-21), *Tg(kdrl:GFP)^s843Tg^* (ref. 64) (Fig. 4a,b), *Tg(ubb:lck-mNeonGreen)^sbu107Tg^* (Figs. 4c-e and 5f-i) and *Tg(hsp70l:DHB.mScarlet-p2a-H2B.miRFP670)^sbu109Tg^* (ref. 32) (Fig. 4c, d).

For xenograft experiments, embryos from the *Tg(kdrl:GFP)^s843Tg^* line were collected and kept at 28.5 °C until 48 hpf. To inhibit melanocyte pigment production for imaging purposes, embryos were treated with 200 μM *N*-phenylthiourea (PTU) (Alfa Aesar) in embryo medium beginning at 6 hpf and exchanged with fresh PTU solution every 24 h. At 48 hpf, embryos were manually dechorionated and anesthetized with 0.003% tricaine (Pentair, TRS1). MDA-MB-231 human breast cancer cells labeled with LifeAct–mRuby were loaded in a glass capillary and injected into the circulatory system by targeting the duct of Cuvier using a CellTram 4r Oil microinjector (50–100 cells per fish)^43^. After the injection, the embryos were maintained in embryo medium with 200 μM PTU at 33 °C until imaging. Embryos with MDA-MB-231 cells in the caudal vascular plexus were selected for imaging. Sample mounting was performed in 0.8% low-melting temperature agarose containing 0.006% tricaine.

For wound-healing studies and tail fin imaging, *Tg(ubb:lck-mNeonGreen)^sbu107Tg^* and *Tg(hsp70l:DHB.mScarlet-p2a-H2B.miRFP670)^sbu109Tg^* transgenic lines were crossed with each other and grown at 28.5 °C. Embryos were heat shocked at approximately the 8–12-somite stage (~12–14 hpf) by shifting embryos from 28.5 °C to 40 °C for 30 min. Following heat-shock, embryos containing both transgenes were sorted under a fluorescence dissecting microscope. For wound healing, at approximately 48 hpf, forceps (Dumont Dumoxel #5) were used to amputate the posterior fin tissue at the tip of the tail, just posterior to the end of the notochord. Sample mounting was conducted in 0.8% low-melting temperature agarose containing 0.006% tricaine and 22% OptiPrep (Sigma-Aldrich).

Plasmids encoding *4x-cox8-stayGold-ev-linker-stayGold* were assembled through synthesis of the gene (Twist) and then cloned using isothermal assembly strategies into a pMTB backbone containing a SP6 promoter upstream of the *4x-cox8-2x-stayGold* complementary DNA^65^. Messenger RNAs were synthesized from linearized plasmid using an mMessage mMachine SP6 Transcription kit (Thermo Fisher Scientific) and purified before injection into the zebrafish embryos using an RNeasy Mini kit (QIAGEN). Then, 30–60 pg of *4x-cox8-2x-stayGold* mRNA was injected into each embryo for imaging (*Tg(eef1a1l1:mem-2x-mchilada)^hm801^*).

These studies were performed using a transgenic ubiquitously expressing membrane targeted red fluorescent protein, mChilada, (*Tg(eef1a1l1:mem-2x-mchilada)^hm801^*). A plasmid with *eef1a1l1* driving expression of 2x-membrane-2x-mchilada was assembled through synthesis of the gene (Twist) and then cloning using isothermal assembly strategies into a tol2 backbone containing the *eef1a1l1* promoter^65^. The transgenic was made by co-injecting 40 pg of plasmid and 40 pg of tol2 mRNA. See the Supplementary Information for the DNA sequences of the zebrafish constructs used to label intracellular and membrane markers.

#### Mouse.

For Fig. 6 and Supplementary Fig. 24, all mouse experiments were conducted at Janelia Research Campus, HHMI in accordance with the US National Institutes of Health (NIH) Guide for the Care and Use of Laboratory Animals. Procedures and protocols were approved by the Institutional Animal Care and Use Committee of the Janelia Research Campus, HHMI. Male or female mice (Jackson Laboratory, C57BL/6J, stock no. 000664; Thy1-GFP line M, stock no. 007788) aged 2–4 months were used in this study. Mice were housed in specific-pathogen-free conditions on individually ventilated racks with 100% outside filtered air in the holding room. They were maintained on a 12-h light–dark cycle at 20–22 °C with 30–70% relative humidity.

All stereotaxic surgeries were carried out under anesthesia (1–2% isoflurane in O_2_) following established procedures. In brief, using a stereotaxic apparatus (Model 1900, David Kopf Instruments) and aseptic technique, mice were anesthetized with isoflurane (1–2% by volume in O_2_). A craniotomy of 3.5 mm in diameter was made over the left dorsal hemisphere of mice with dura left intact. For mice that required virus injection, a glass pipette (Drummond Scientific Company) beveled at 45° with a 15–20-μm opening was back-filled with mineral oil. A fitted plunger controlled by a hydraulic manipulator (Narishige, MO10) was inserted into the pipette and used to load and slowly inject 10–20 nl viral solution into the brain at ~200–400 μm below pia. The following injection coordinates were chosen to label brain regions in one or both hemispheres: site 1 (Bregma −4.6 mm, midline 2.1 mm), site 2 (Bregma −4.6 mm, midline 2.8 mm), site 3 (Bregma −4.1 mm, midline 2.1 mm) and site 4 (Bregma −4.1 mm, midline 2.8 mm). For sparse labeling of neurons in one hemisphere, AAV2/1.syn.FLEX.GCaMP7s (1 × 10^13^ GC ml^−1^) was mixed with AAV2/1.syn.Cre (1 × 10^13^ GC ml^−1^ diluted 10,000 times) at 1:1 for injection into wild-type mice. At the completion of viral injections or craniotomy without viral injections, a cranial window made of a single glass coverslip (Thermo Fisher Scientific, no. 1.5) with a diameter of 3.5 mm was embedded in the craniotomy and sealed in place with Vetbond tissue adhesive (3M). A titanium headpost was then attached to the skull with cyanoacrylate glue and then dental acrylic. Mice were given the analgesic buprenorphine (subcutaneously, 0.3 mg kg^−1^ of body weight). In vivo imaging was carried out after 4 weeks of expression for AAV injected animals and immediately for Thy1–GFP mice. For blood vessel imaging, 50 μl 5% dextran conjugated Texas Red (70 kDa) was injected retro-orbitally in the animal and anesthetized with isoflurane (maintained at 1–2% by volume in air).

#### Drosophila.

For Supplementary Fig. 18a, to generate the UAS–Halo7–EB1 construct, the EB1 coding sequence was PCR amplified from the genomic DNA of a UAS–EB1–GFP fly^66^ and subsequently cloned into UAS–Halo7::CAAX (Addgene #87645) through XhoI and XbaI. The UAS–Halo7–EB1 plasmid was then used to generate transgenic flies. Flies were raised on standard cornmeal medium with a 12-h light cycle at 25 °C. The pebbled–GAL4, FRT19A/tubP–GAL80, hsFLP, FRT19A; UAS–mCD8–GFP/UAS–Halo7–EB1 flies were heat shocked 2 days before puparium formation at 37 °C for 35 min to induce sparse clones using the mosaic analysis with a repressible cell marker (MARCM) strategy^67^.

#### C. elegans.

For Supplementary Fig. 18b, animals were reared under standard conditions at 25 °C (ref. 68). Animals were synchronized for experiments through alkaline hypochlorite treatment of gravid adults to isolate eggs^69^. In the text and figures, we designate linkage to a promoter through the use of the (p) symbol and fusion of proteins via a (::) annotation. The following transgene was used in this study: LG I bmdSi86[LoxN::rps-27:DHB::GFP::P2A::H2B::mKate2]. L3 stage larvae were anesthetized using 5 mM levamisole in M9 buffer^32^ and mounted in a drop of 1.0% low-melt agarose supplemented with 5 mM levamisole for live imaging.

#### Human brain organoids.

For Supplementary Fig. 18c, all human stem cell experiments were carried out in compliance with the Human Stem Cell Research Oversight (hSCRO) Committee of the University of California, Irvine. Cytoplasmic-mGFP-expressing WTC-11 induced pluripotent stem cells (iPSCs) (Coriell, AICS-0036-006) and WTC-11 iPSCs (Coriell, GM25256) were cultured on Vitronectin XF (Stem Cell Tech, 07180) in NutriStem hPSC XF Medium (Sartorius, 05-100-1A). Brain organoids were generated following the protocol by Lancaster and Knoblich^70^ with a few modifications. In brief, GFP-expressing iPSCs were mixed with unmodified WTC-11 iPSCs at a ratio of 1:5, and embryoid bodies (EBs) were generated from 9,000 cells per well in EB formation medium in 96-well, non-treated, V-bottom plates (Corning, 3896), pretreated with anti-adherence rinsing solution (STEMCELL Technologies, 07010) at 37 °C. The EBs that formed after 5 days were transferred to Neural Induction Medium (see below for compositions of all media used) and cultured for 4 more days. The 9-day-old EBs were embedded in Matrigel (Corning, CB-40234A) and grown in Neural Differentiation Medium for 4 more days. On day 13, the EBs were transferred to Neural Maturation Medium in 50-ml conical culture tubes. The conical tube caps were kept loose in a CO_2_, 37 °C incubator until the medium color indicated equilibration with CO_2_, and then the caps were tightened. The tubes were then placed in a rotator (Fisherbrand Mini Tube Rotator, 88-861-051) set at 20 rpm at 37 °C with weekly medium changes. The tubes remained horizontal while being rotated. Organoids were imaged on day 27 after iPSCs were switched to EB formation medium. EB formation medium consisted of NutriStem hPSC XF GF-free (Sartorius, 06-5100-01-1A), 4 ng ml^−1^ bFGF (Peprotech, 100-18b) and 10 μM Y-27632 (Biological Industries, SM-0013-0010). Neural Induction Medium consisted of DMEM/F12 (Gibco, 11330-032) with 1% (vol/vol) N2 supplement (Gibco, 17502048), 1% GlutaMAX supplement (Gibco, 35050038), 1% MEM–NEAA (Gibco, 11140076) and 1 μg ml^−1^ heparin (Sigma, H3149). Neural Differentiation Medium consisted of 50% DMEM/F12 and 50% Neurobasal Medium (Gibco, 21103049) supplemented with 0.5% N2 Supplement, 0.5% B27 without vitamin A (Gibco, 12587010), 2.5 μg ml^−1^ insulin (Sigma, I9278), 50 μM 2-mercaptoethanol (Sigma, M3148), 1% GlutaMAX supplement, 0.5% MEM–NEAA and 1× penicillin–streptomycin (Gibco, 15140122). Neural Maturation Medium is the same as Neural Differentiation Medium, except that B27 supplement (without vitamin A) is replaced with B27 supplement (with vitamin A) (Gibco, 17504044).

### Imaging conditions

Detailed imaging conditions for each experiment can be found in Supplementary Table 1. All abbreviations used in this paper are defined in Supplementary Table 2.

### Image processing

#### Image preprocessing.

Image preprocessing including flat-field correction, deconvolution, deskewing and rotation, and stitching was performed with PetaKit5D, which has been extensively documented and demonstrated^13^.

#### Nuclei segmentation.

To expedite segmentation, we initially downsampled the data threefold in *x* and *y*. We performed a first pass at nucleus detection using Cellpose^71^. For each detected nucleus, we cropped a local region, defined by its bounding box extended with a 20-voxel buffer in *x*, *y* and *z*. Otsu’s thresholding was then applied to this local region to refine the segmentation. We utilized the ER channel to exclude cytosolic regions. Segmentations were further refined by smoothing, hole filling and taking the union with an extended Cellpose mask; for this extension, the top and bottom *z*-slices were extrapolated by replicating the first and last available slices, respectively.

Subsequently, we merged duplicate masks that corresponded to the same nucleus. To recover mitotic nuclei missed by Cellpose, we applied a high-intensity threshold (95th percentile of a Gaussian-smoothed image, σ = 0.5) and incorporated the resulting masks into the final segmentation. All masks were then resampled to the original data resolution. Finally, to address incomplete segmentation near the coverslip, we identified slices containing open holes and filled these using information from the immediately preceding slice.

#### Denoising.

Content-aware image restoration (CARE)^21^ was used to denoise raw images acquired by AO-LLS-SIM and fast, extended-duration 3D-SIM. To train the denoising models, phase-stepped raw images were collected over the same region of interest both at the experimental illumination settings and at ‘ground-truth’ illumination settings (~10× higher SNR). Separate models were trained and applied to each channel. Denoising was performed on raw phase-stepped images before SIM reconstruction.

#### SIM reconstruction.

We used GPU-accelerated SIM reconstruction software (https://github.com/scopetools/cudasirecon)^72^ with an OTF calculated from an experimentally measured PSF to reconstruct data collected in the LLS-SIM and 3D-SIM mode. We adapted this code to use cosine apodization during reconstruction. To minimize reconstruction artifacts, we split the data into 128-pixel chunks (with a 32-pixel overlap at each border) in image *xy*^73^.

#### Multimodal registration.

To enable multimodal analysis, we calibrated the instrument to achieve near-pixel-level registration between datasets acquired via different objective paths (for example, inverted versus light-sheet objectives). Initial coarse alignment between the objective paths utilized cellular structures that could be correlated across different objective views, followed by fine refinement using diffraction-limited beads. When needed, final adjustments to correct minor residual misalignments between modalities were performed by manually measuring the offsets and using PetaKit5D.

#### Membrane enhancement.

Where noted, the plasma membrane structures in zebrafish data were enhanced using a ridge detection model trained with CARE. The training data were generated using both the raw image and its corresponding tensor voting (TV) filter image calculated as described in ACME^74^. Each image was first smoothed (3D Gaussian, σ = 0.8 for raw, σ = 0.5 for TV) and then masked using the morphologically closed (cubic kernel size 3) nonzero voxels from the TV image. Within these masked regions, ridge likelihood was estimated by computing the eigenvalues of the Hessian matrix for four iterations. If the largest absolute eigenvalue was negative, its absolute value was used to update the corresponding voxel in the eigenvalue image before the next iteration. The resulting ridge likelihood maps were binarized using a threshold (minimum of 0.01 and the fifth percentile of nonzero values) and small holes (<20 voxels) were filled. The binary ridge masks from the raw and TV paths were combined using a union operation. Finally, this combined mask was smoothed (3D Gaussian, σ = 1) and multiplied element-wise with the original raw image to enhance membrane features. The CARE model was trained to predict the enhanced membrane features with the original raw image as input.

#### ISM reconstruction.

The ISM dataset was reconstructed into either a 2D confocal image or a 2D ISM image. The confocal image was generated by summing within a virtual slit, followed by deconvolution. The full ISM image was produced by deconvolving each row individually with its corresponding PSF and then combining the results.

##### Preprocessing.

All raw camera images underwent background subtraction by removing a recently acquired dark image. Next, any bad pixels (identified via extended exposures that reveal abnormally noisy pixels) were excluded from downstream analysis. To locate and align the slit, a MIP was computed from the sample dataset and the brightest points in each image column (above 30 counts) were peak-detected. A linear fit then yielded the slit’s angle and position. Finally, the images were digitally rotated (via bilinear interpolation) so that the slit aligned with the pixel rows.

##### 3D confocal reconstruction.

To measure the 3D confocal PSF, isolated bead data were acquired across *x*, *y* and *z* while applying the same preprocessing steps. In each camera image, the center column (defined by the slit’s location) was masked with a 6.4-pixel-wide (1 AU) rectangular window, and its intensities were summed; if the mask subtended a fraction of a pixel, that fraction was added proportionally. Plotting these sums at their corresponding *x*, *y* and *z* positions generated a volumetric dataset whose center was determined by 3D Gaussian fitting. For confocal imaging of a sample, line-scan illumination was performed along *x* while stepping the *y* galvo and *z* stage. The same slit-centered rectangular mask was applied, each column was summed, and these sums formed voxels in the final 3D volume. Optionally, the volume was deconvolved in MATLAB using the measured PSF and ‘deconvlucy’, yielding a refined 3D confocal result.

##### 3D ISM reconstruction.

In 3D ISM, the excitation PSF (ePSF) was measured by acquiring bead images at multiple *x*, *y* and *z* positions, summing each camera frame, and plotting those totals as a 3D volume. The bead’s position was localized in subvoxel space by examining neighboring camera images and centering the bead via 2D Gaussian fits. The detection PSF (dPSF) was measured separately under widefield illumination, and its center was found by 3D Gaussian fitting of the top third of pixel values. A total PSF was then computed for each of the seven pixel rows closest to the slit center by convolving a row-specific, laterally shifted ePSF with the row-centered dPSF. Sample data were collected similarly to the confocal case, and each of the seven rows formed its own 3D volume. Each volume was deconvolved independently with the corresponding total PSF (again using ‘deconvlucy’) and the seven resulting volumes were summed to yield the final 3D ISM reconstruction. While intermediate iterative fusion was possible, in practice the final summation generally provided comparable results with substantially less computation time.

#### Single-molecule fitting for PAINT.

SR PAINT imaging and reconstruction was conducted as described previously^57^.

#### Single-particle tracking.

Individual nuclei were manually cropped using a custom Python (v.3.8.8) script. Single molecules were detected and tracked using TrackMate (v.7.12.2)^75^ and localizations were identified with a Laplacian-of-Gaussian detector.

#### Diffusion analysis.

Diffusion analysis was performed using custom Python (v.3.8.8) scripts. Single-molecule trajectories with fewer than four consecutive localizations were excluded from further analysis. For each linked particle within a trajectory, the mean squared displacement was calculated for time intervals ranging from 20 ms to 200 ms. Linear regression was applied to this relationship. A histogram of the resulting values was then fit using a two-component Gaussian mixture model via the scikit-learn function GaussianMixture^76^.

#### 3D spine intensity estimation.

We automatically detected and tracked dendritic spines in 2D functional imaging datasets using custom MATLAB scripts, modeling spines as 2D Gaussians^77^. Spine candidates were first identified using a Laplacian-of-Gaussian filter, and then were fitted with a 2D Gaussian with a fixed sigma of 3 pixels. The detected spines were then tracked with a frame-to-frame linking search radius of 3–6 pixels. These parameters were identical for both AO-corrected and uncorrected data. For comparison, calcium events were quantified based on the maximum fitted intensity per tracked spine activity, including only tracks persisting for at least 2 s.

#### Cell cycle quantification.

First, we corrected raw DHB (CDK sensor) and nuclear intensity stacks for camera background offset. We then generated 3D cell masks using previously described methods involving segmentation^74^ and manual curation. For each resulting cell mask, we extracted a corresponding cubic region from the intensity data. Both the mask and intensity subvolumes were resampled to isotropic voxels using nearest-neighbor (mask) and linear (intensity) interpolation, respectively.

From these resampled masks, we calculated cell volume (total mask voxels multiplied by the voxel volume) and estimated surface area (from 3D perimeter). Within each cell mask, we segmented nuclei using 3D Gaussian smoothing (σ = 1 pixel), followed by Otsu thresholding and connected-component analysis (26-connectivity). To ensure data quality, we excluded cells truncated at imaging boundaries or those falling below minimum volume/area thresholds (potentially indicating incomplete cells or low expression). We calculated cytoplasmic-to-nuclear DHB ratios for each cell by summing the DHB intensity within the segmented nucleus and the remaining cytoplasmic region (defined by the cell mask excluding the nucleus). Filtered segmentation masks, and DHB ratio maps were saved as single-precision 3D TIFF volumes and visualized using Amira (Thermo Fisher Scientific). All image processing and analysis steps were performed using custom scripts in MATLAB R2022a.

#### Mitochondria quantification.

After cells were segmented, MitoGraph was used to analyze the 3D morphology of mitochondria within each isolated cell, including both segmentation and skeletonization for total mitochondria length calculation^78^.

#### Removal of digitization artifacts in two-photon imaging.

Ratio frequency noise introduced during two-photon detection with the MPPC detectors manifested itself as discrete frequencies in the Fourier transform of the time-ordered one-dimensional data as originally measured. Unfortunately, the acquisition hardware did not return a time trace, but rather a 2D image, where the time of acquisition determined the values of the pixels in a rectilinear grid. The noise was then distributed across the image. Furthermore, because the TPM data were acquired with a resonant galvo, the physical location between adjacent measurements was further at the center of the image than at the edges. To correct these issues, the 2D pixels were converted back to a one-dimensional temporal waveform but interpolated to their known time of acquisition on an axis with 38-ns spacing. The Fourier transform of this waveform then revealed the noise as sharp peaks that could be removed with notch filters. Finally, the waveform was converted back to an interpolated 2D image where the datum for each time point was mapped back onto the physical location of where the excitation focus was located at that time.

#### Motion stabilization during two-photon imaging in mice.

Even for a head-fixed live mouse, residual motion (for example, respiration) could still appear in the data both within and between individual images; however, as the data were acquired in *y* with a fast resonant scanner, over any sufficiently small band of columns (typically 32–128) the motion would seem negligible. This was cross-correlated to the same band of columns in another image in the time series where the motion in the column of interest was negligible (for example, <6 pixels), yielding the mean displacement of the column in the moving case. The displacements of all such column groups across the image were fitted to a spline curve, and each individual column in the moving image was shifted according to the interpolated displacement from this curve. Finally, these corrected images were cross-correlated over time to determine their relative global displacements, and these were corrected at the subpixel level by bilinear interpolation.

#### Visualization and software.

Videos and figures were made with Imaris (Oxford Instruments), Fiji^79^, Agave (Allen Institute, https://github.com/allen-cell-animated/agave), Amira (Thermo Fisher Scientific), IndeX (NVIDIA) and MATLAB R2019a-R2024a (MathWorks) software. Only for visualization, gamma adjustment was applied in addition to the image-processing steps noted above.

## Supplementary Material

Supplementary information

Video 2

Video 3

Video 1

Video 4

Video 5

Video 6

Video 7

Video 8

Video 9

Video 10

Video 11

Video 12

Video 13

Video 14

Video 15

Video 16

Video 17

Video 18

Video 19

Video 20

**Supplementary information** The online version contains supplementary material available at https://doi.org/10.1038/s41592-026-03066-1.

## Data Availability

The datasets for this manuscript exceed the size limits of public data repositories, but they will be shared upon reasonable request.

## Code availability

Microscope control software is available through a research license agreement with HHMI. PetaKit5D^13^ data preprocessing software is available on GitHub at https://github.com/abcucberkeley/PetaKit5D.
