# Supplementary material for: A multimodal adaptive optical microscope for in vivo imaging from molecules to organisms
Source: Nat Methods. Author manuscript; Available in PMC 2026 Jun 28. (PMC13310421; doi:10.1038/s41592-026-03066-1)
Supplement: Supplementary information [file NIHMS2185038-supplement-Supplementary_information.pdf]

nature methods

Supplementary information https://doi.org/10.1038/s41592-026-03066-1

A multimodal adaptive optical microscope for in vivo imaging from molecules to organisms

In the format provided by the authors and unedited

Supplementary Figures

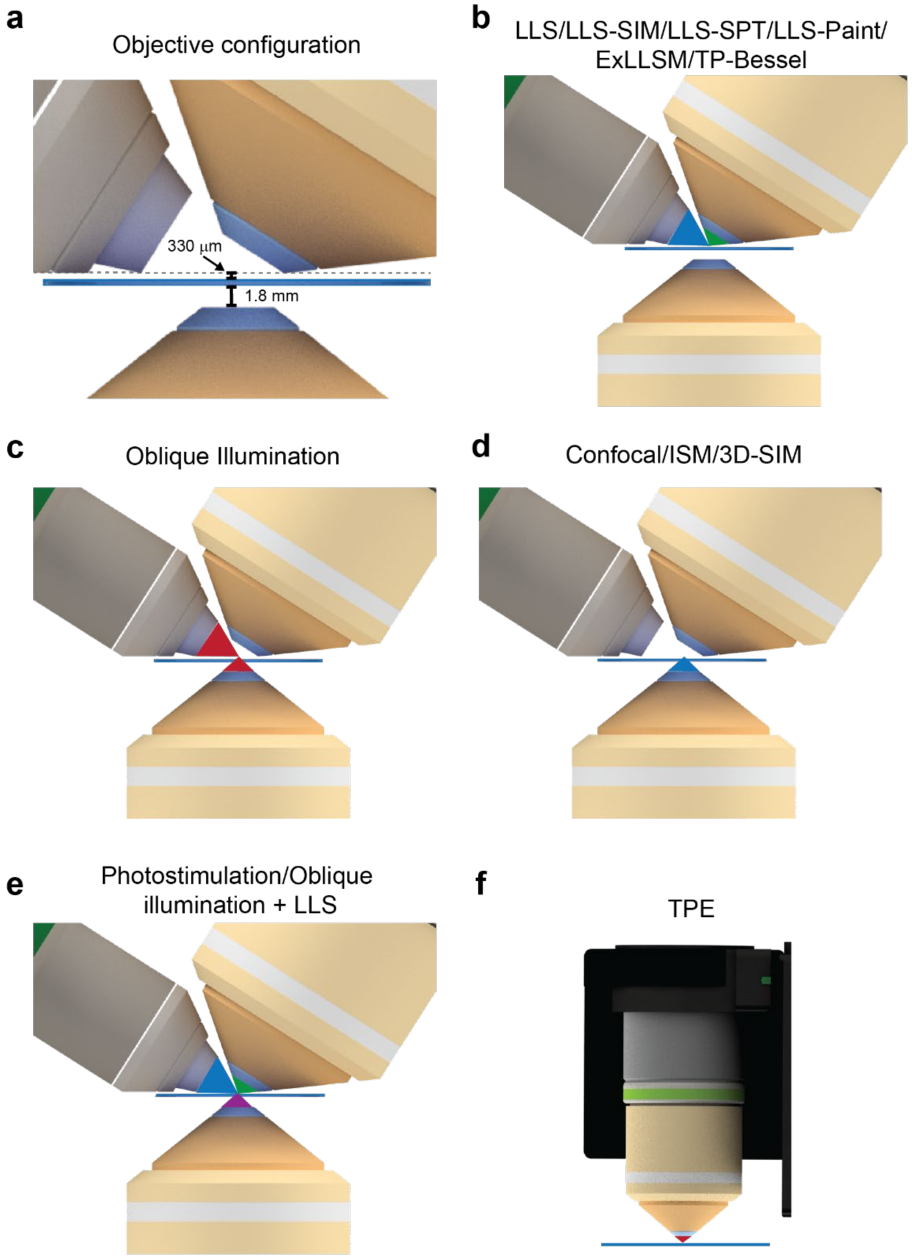

Supplementary Figure 1. Objective configurations for each imaging modality. (a) Spatial arrangement of the three co-focal objectives of MOSAIC, showing the 330 μm working distance between the sample coverslip and the corners of the light sheet excitation (left) and detection (right) objectives, as well as the 1.8 mm working distance for the inverted objective. **(b-f)** Light cones indicating the objectives used for each modality: (b) Lattice light sheet (LLS) microscopy and modalities derived from it, including structured illumination (LLS-SIM), single-particle tracking (LLS-SPT), PAINT (LLS-PAINT), expansion (ExLLSM), and two-photon Bessel light sheet microscopy (TP-Bessel); (c) Oblique illumination (OI); (d) Confocal, image scanning, and 3D structured illumination microscopy (3D-SIM); (e) Photostimulation combined with OI and/or LLSM; (f) Upright two-photon microscopy at a dedicated station for mouse imaging.

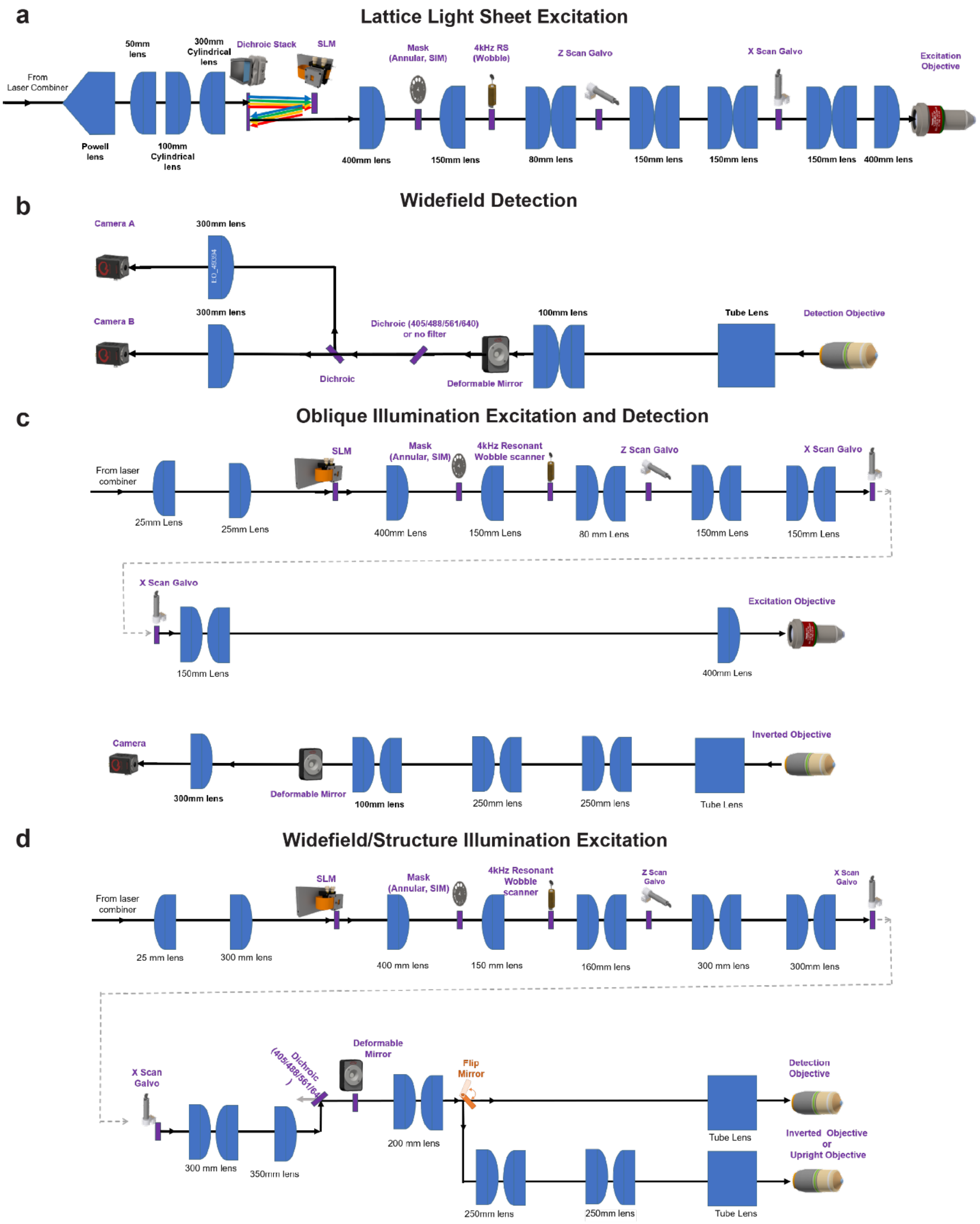

Supplementary Figure 2. Optical paths: Part 1. (a) Lattice light sheet excitation, **(b)** Widefield detection, **(c)** Oblique illumination excitation and detection, and **(d)** Widefield/structured illumination excitation. Fixed position optical elements are labeled in magenta and variable position optical elements are labeled in orange.

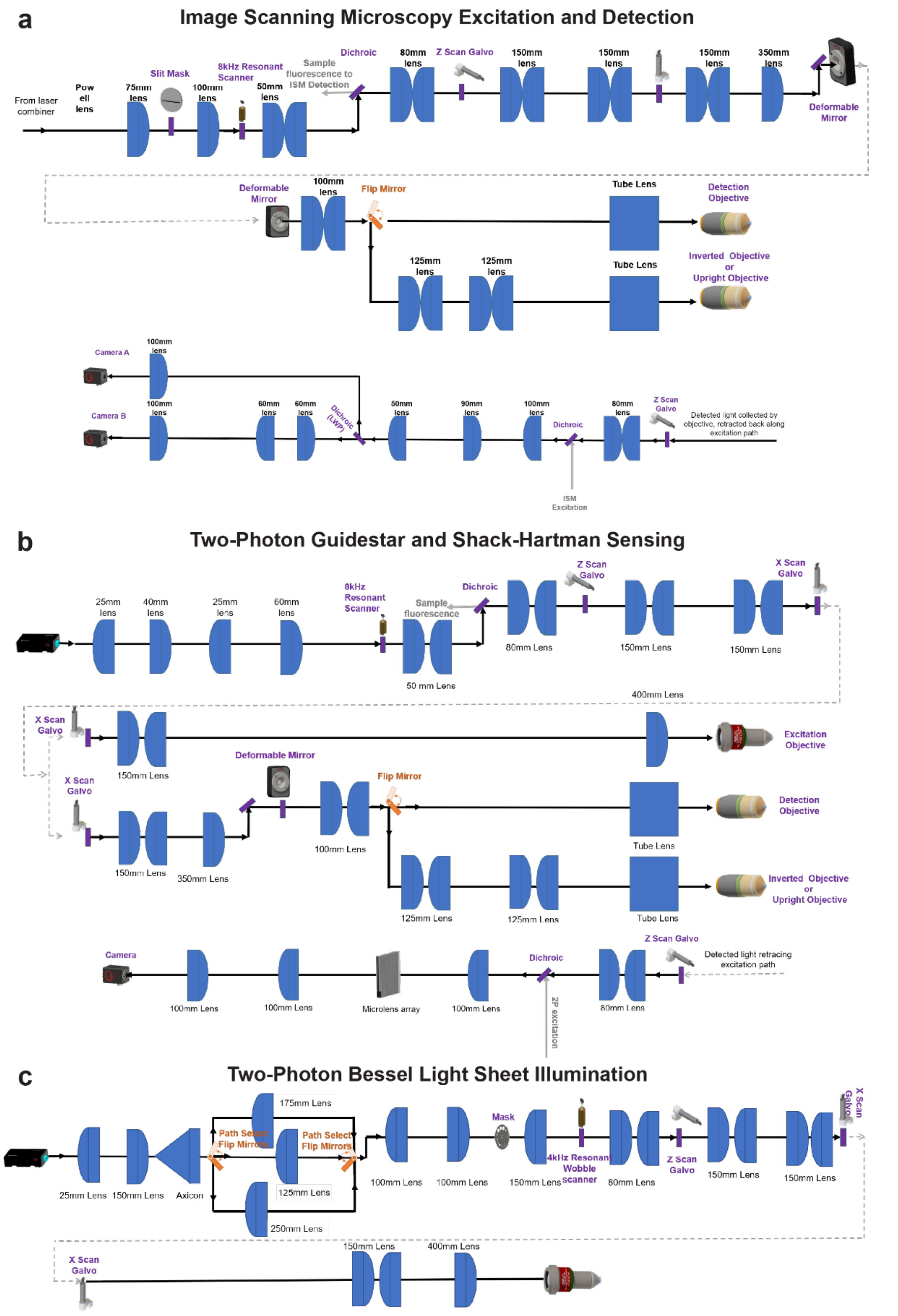

Supplementary Figure 3. Optical paths: Part 2. (a) Image scanning microscopy, **(b)** Two-photon guidestar and Shack-Hartman sensing, and **(c)** Two-photon Bessel light sheet microscopy.

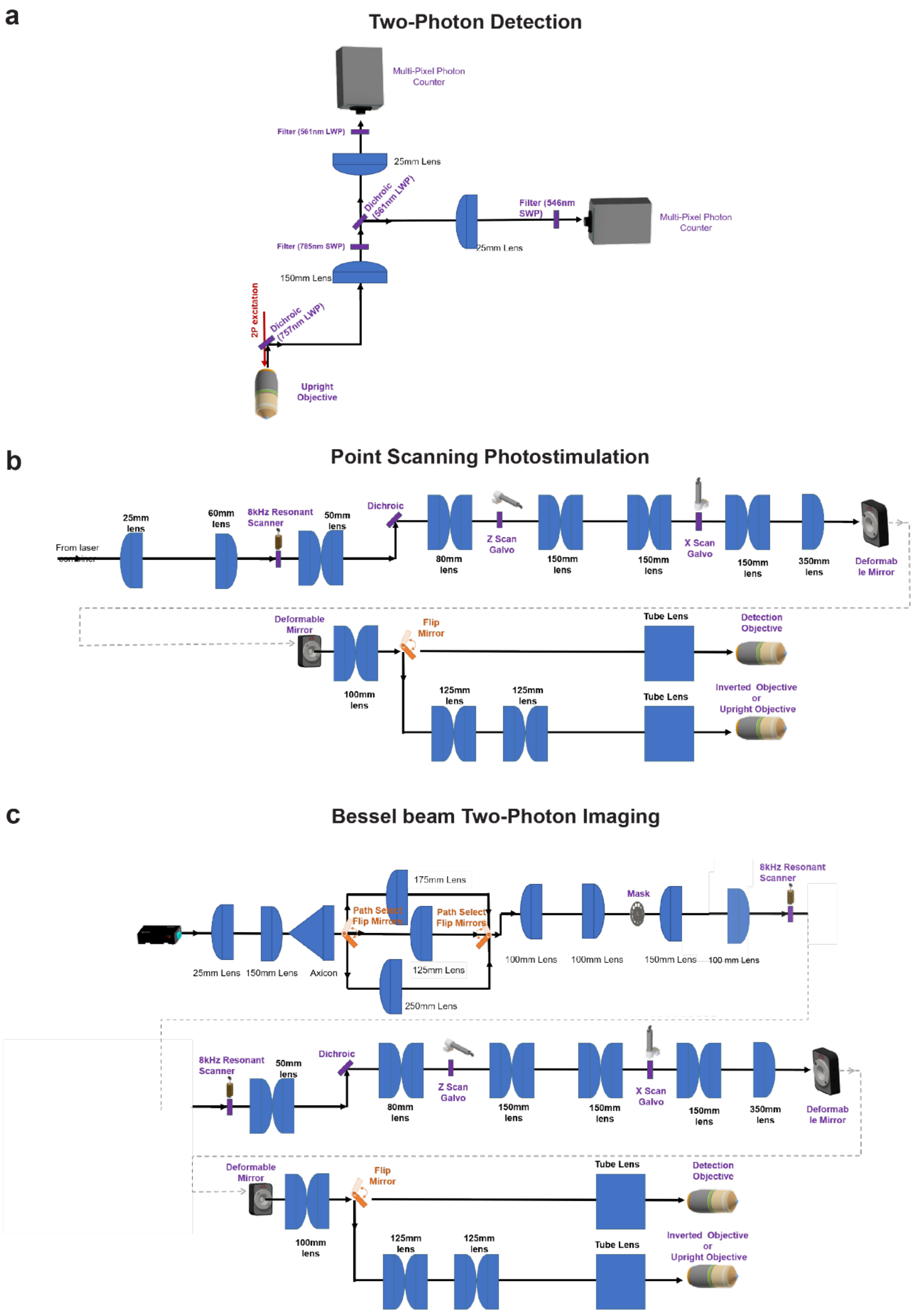

Supplementary Figure 4. Optical paths: Part 3. (a) Two-photon point scanning detection, **(b)** Point scanning photostimulation, and **(c)** Bessel beam two-photon scanning.

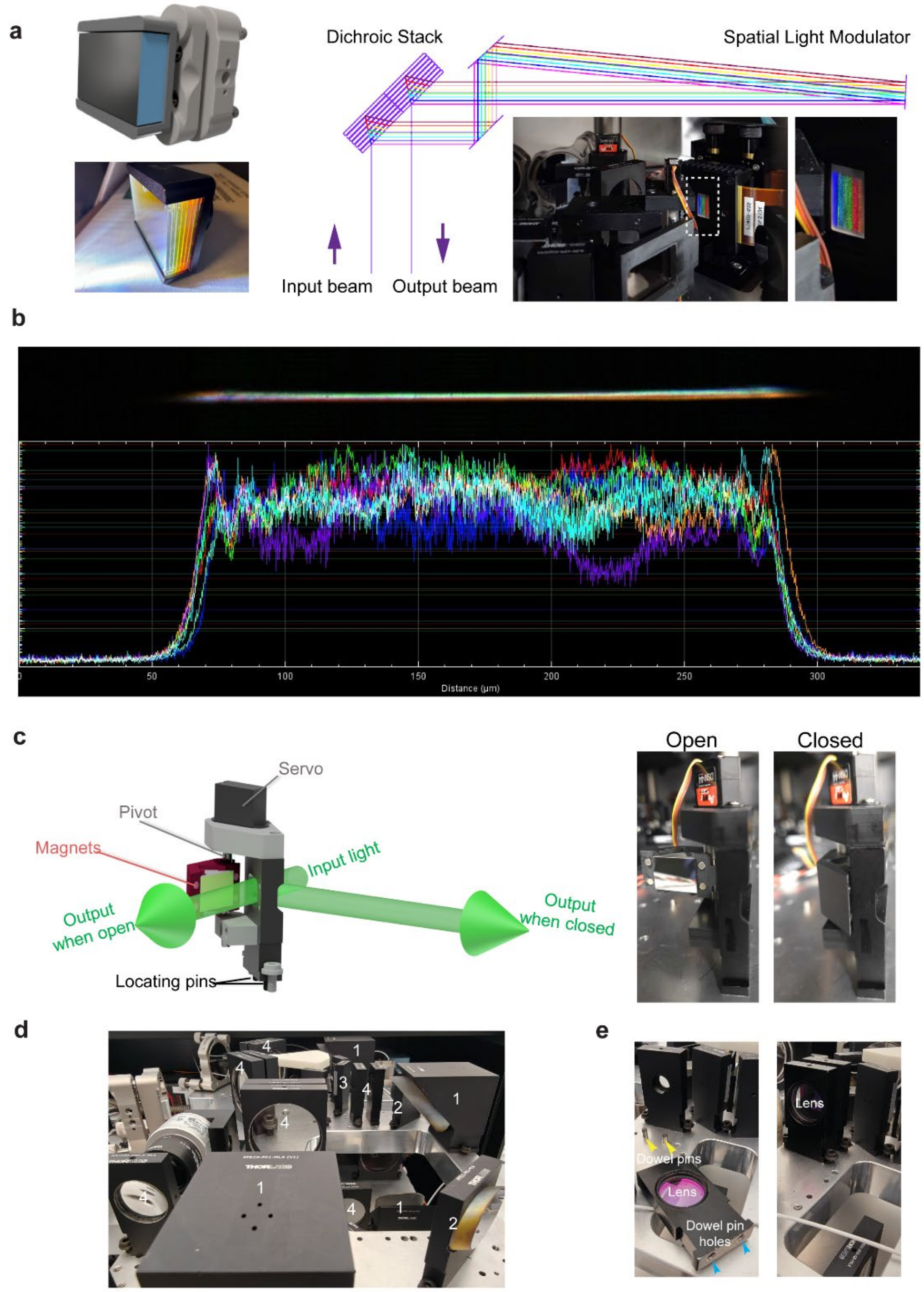

Supplementary Figure 5. Novel optical elements used in MOSAIC. (a) Custom dichroic mirror stack consisting of six parallel air gapped dichroic mirrors and one reflective mirror. The stack spectrally separates a co-aligned multicolor input beam, directing each wavelength to a distinct region on a spatial light modulator (SLM) for customized lattice light sheet pattern generation and modification. The reflected beams are recombined by the stack into a single multicolor light sheet of cross-sectional intensity shown in **(b)**. **(c)** Flip mirror mechanism to toggle between orthogonal optical paths. Right: flip mirror in its open (pass through mode) and closed (reflection mode) positions. **(d)** Examples of installed pre-centered optics. 1: vertical 90° elliptical mirror; 2: horizontal 90° elliptical mirror; 3: horizontal square mirror; 4: lens and lens doublet. **(e)** Dowel pins (yellow arrows), and holes (blue arrows) help with precision installation of pre-centered optics.

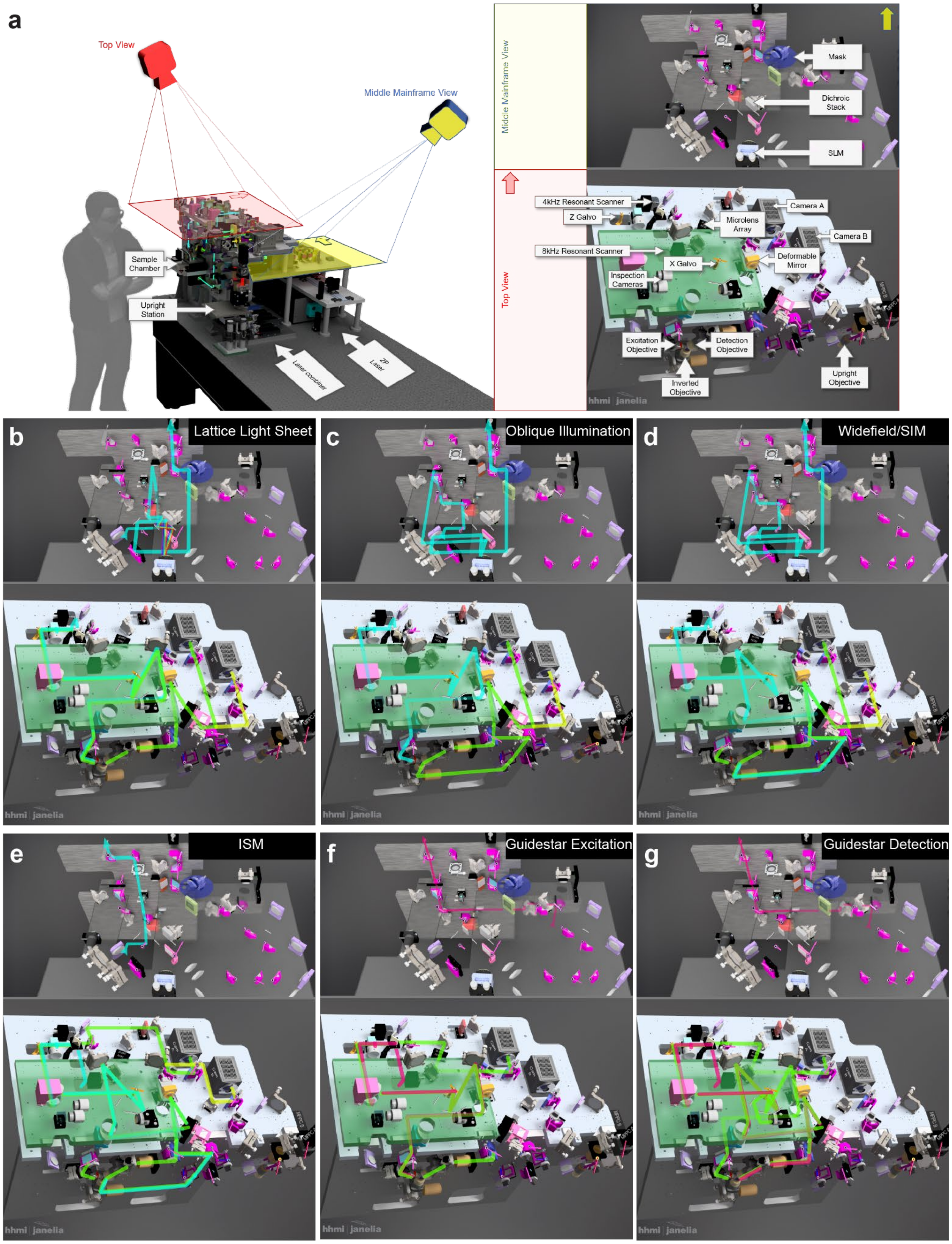

Supplementary Figure 6. 3D model with beam paths used by different modalities: Part 1. (a) Left: Overall MOSAIC architecture, with view directions defined for subsequent panels. Right: Major MOSAIC components as seen from these two views. **(b-g)** Optical pathways as seen from the two views for the MOSAIC modes given at upper right of each panel.

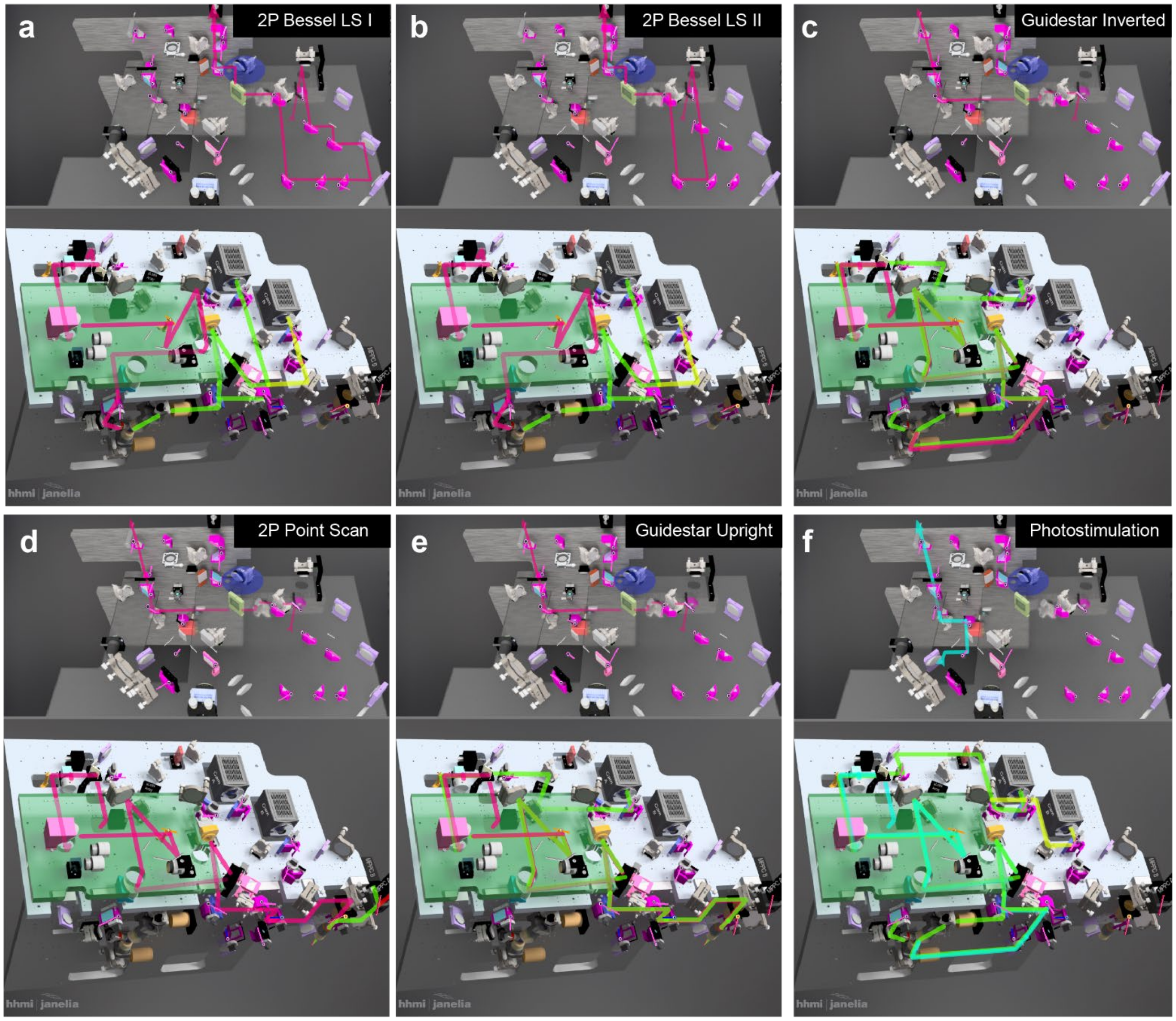

Supplementary Figure 7. 3D model with beam paths used by different modalities: Part 2. Optical pathways for six more MOSAIC modes as described at upper right of each panel, as seen from the two views in Supplementary Figure 6a.

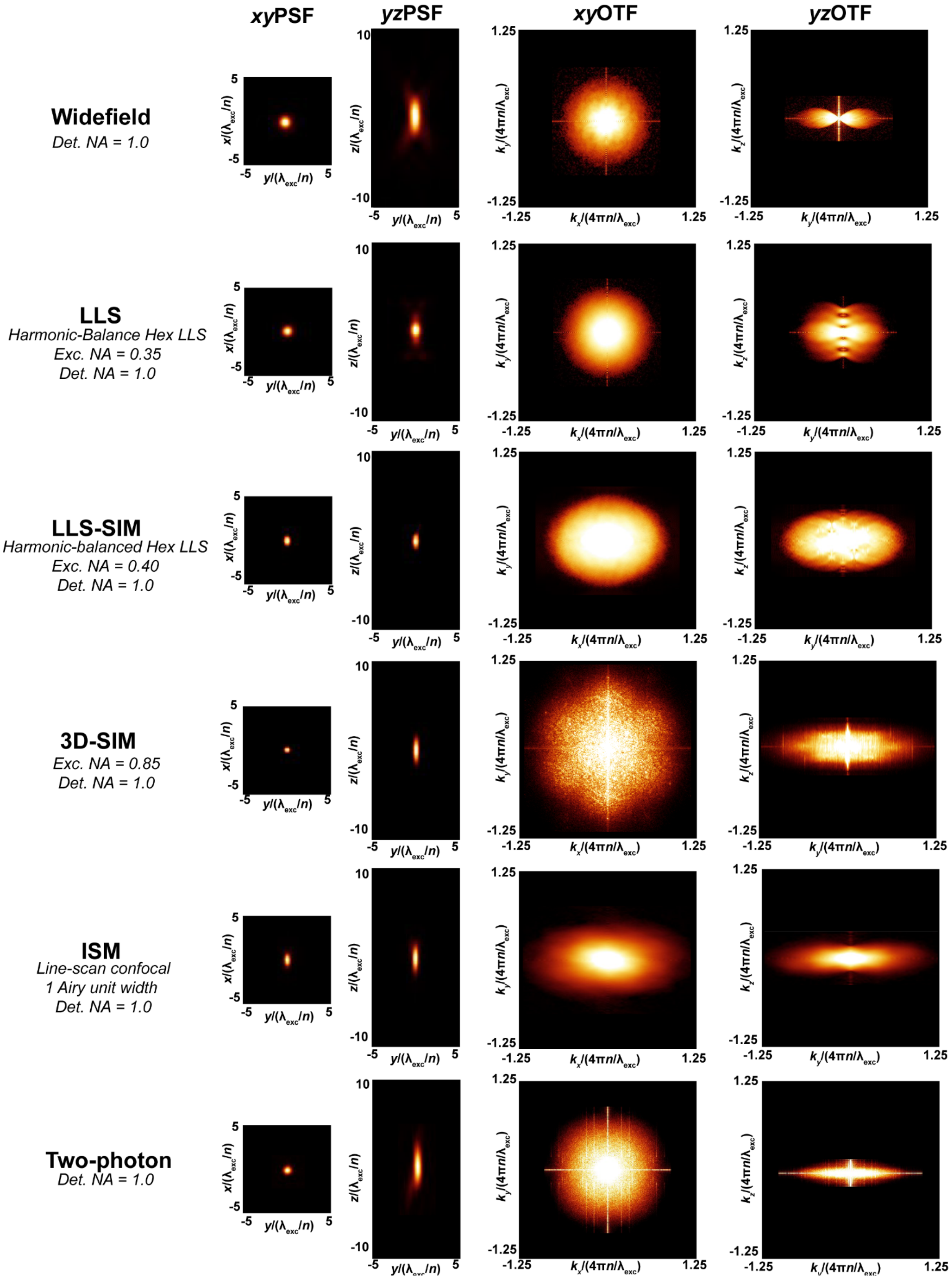

Supplementary Figure 8. Experimental PSFs and OTFs for different MOSAIC modes. Gamma = 0.5 was applied to the OTF plots.

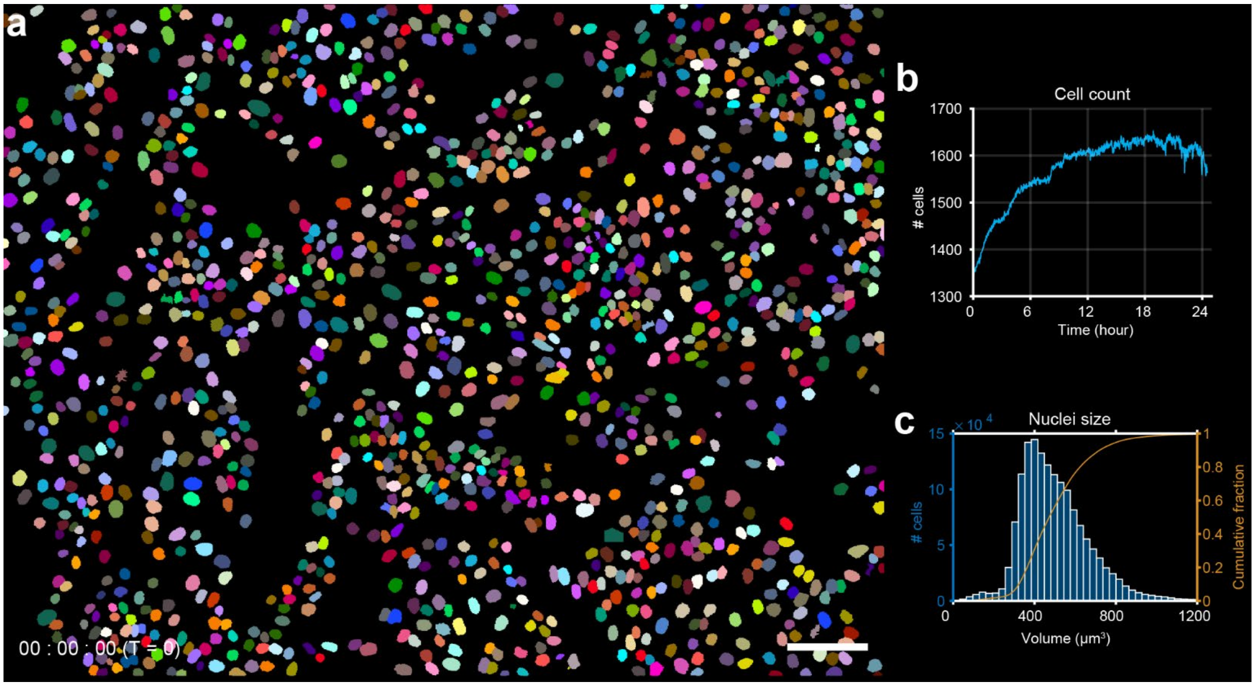

Supplementary Figure 9. Analysis of nuclear dynamics from long-term imaging of LLC-PK1 cells. (a) MIP of segmented nuclei, randomly colored, at the start of a 24-hour imaging experiment (t=0). Scale bar; 100 μm. **(b)** Cell population growth, as measured by number of segmented nuclei, over the 24-hours. **(c)** Histogram (left axis) and corresponding cumulative distribution function (right axis) for all nuclear volumes measured across all time points. From Supplementary Video 2.

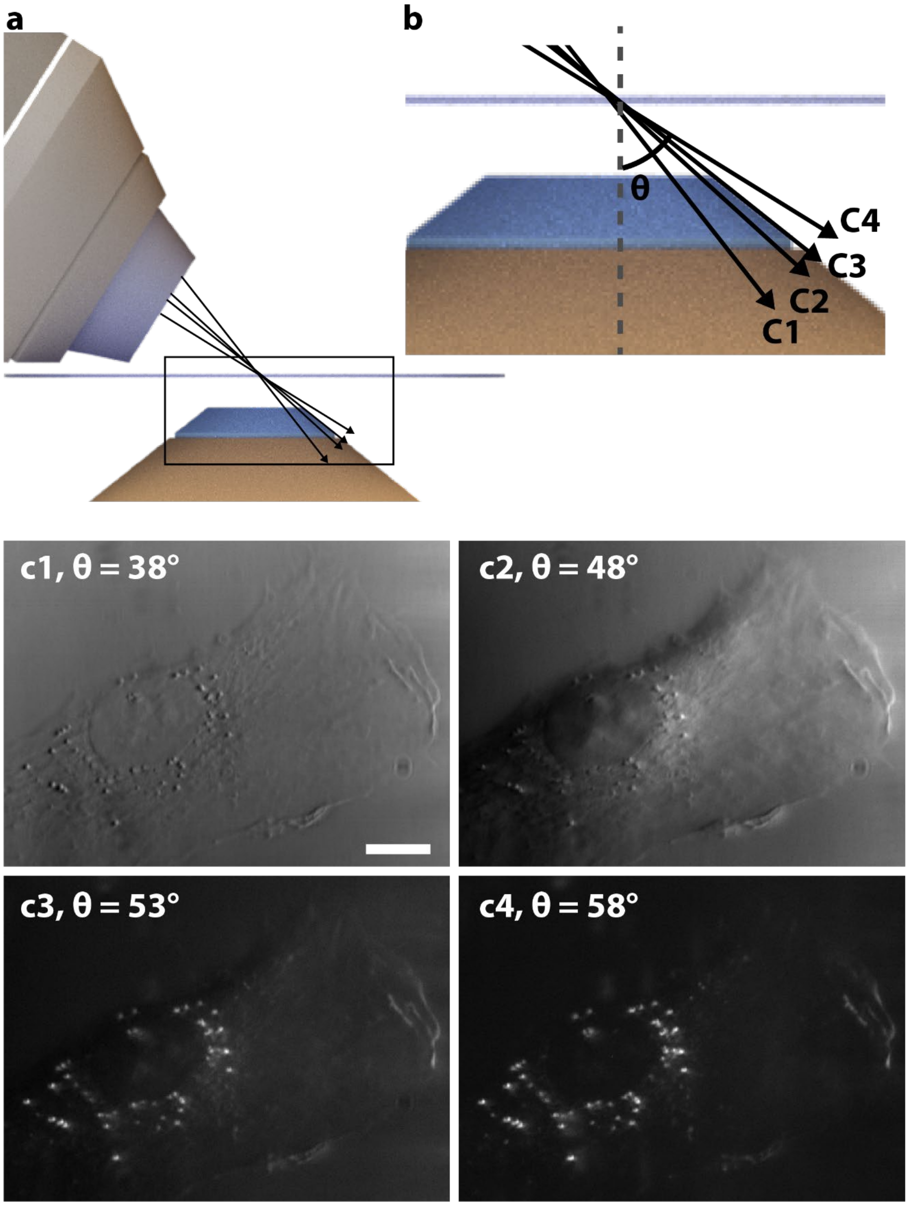

Supplementary Figure 10. Oblique illumination contrast vs. illumination angle. (a) Excitation objective provides illumination oblique at a single angle to the axis of the inverted objective used for imaging. **(b)** Enlarged view showing four angles (θ) ranging from all ballistic light collected (c1) to all ballistic light rejected (c4). Corresponding images of a cell show contrast ranging from brightfield (c1, θ = 38°), to DIC-mimicking (c2, θ = 48°), to progressively darker field (c3, θ = 53°, c4, θ = 58°). Scale bar, 5 μm.

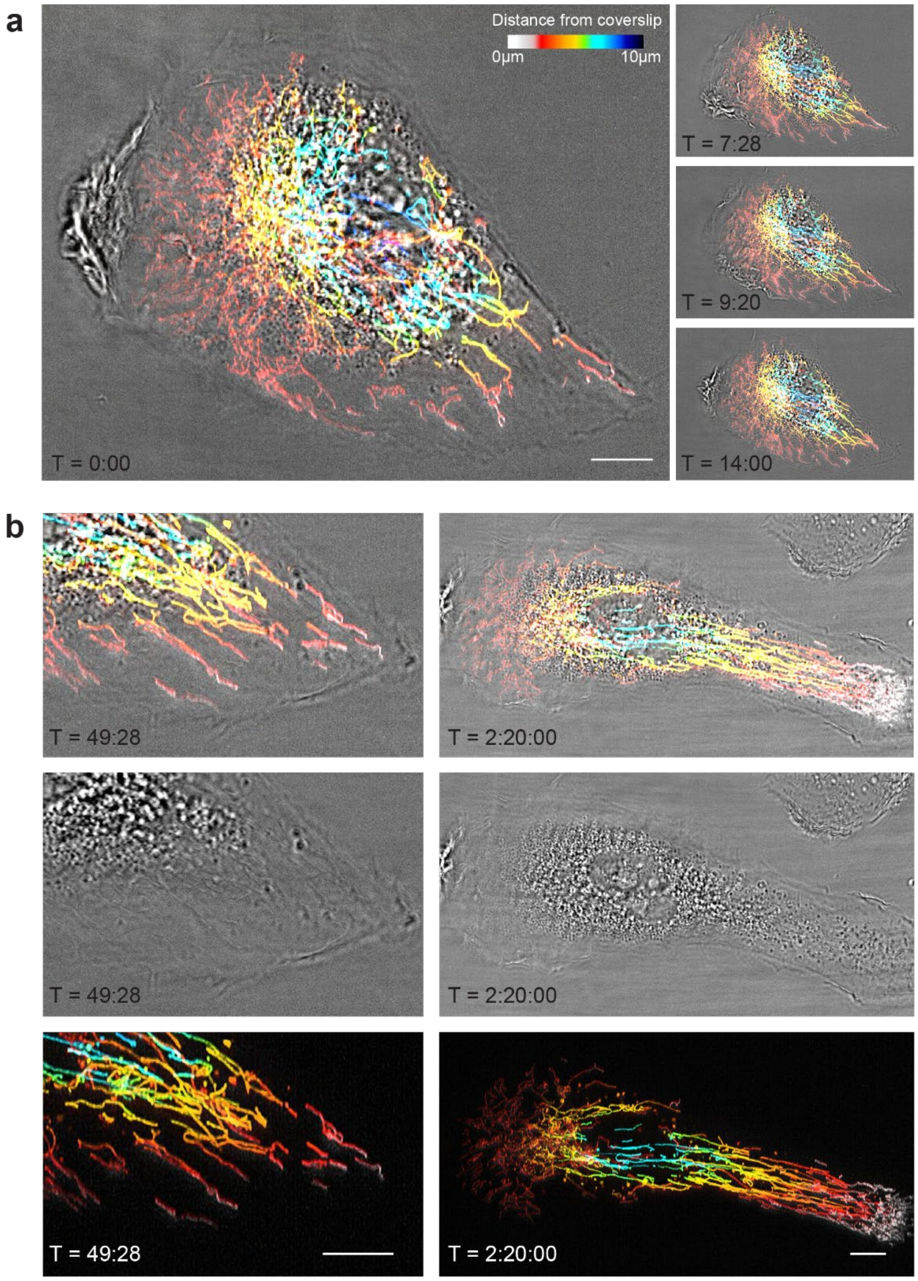

Supplementary Figure 11. Correlative LLS and OI imaging of a migrating hTERT-RPE1 cell. (a) Overlay of correlative imaging highlights both mitochondria (COX8a-StayGold imaged with LLS, color coded by height) and overall cell morphology (OI). Scale bar, 5 µm. **(b)** Overlay (top), OI (middle), and LLS fluorescence (bottom) at two time points highlight the leading edge during migration and the exclusion of mitochondria from the cellular periphery. Scale bar, 5 µm. See Supplementary Video 4.

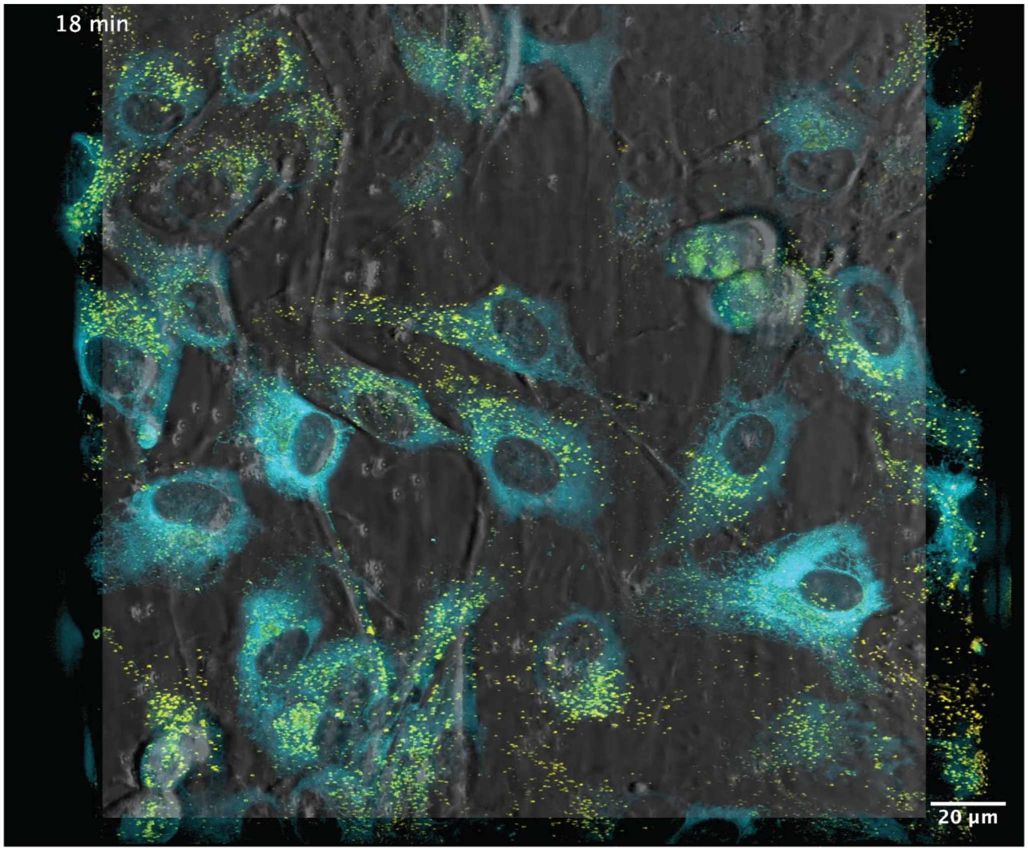

Supplementary Figure 12. Large FOV correlative LLS and OI imaging. Overlay of LLS and OI imaging of hTERT-RPE1 cells co-expressing ER (calreticulin-StayGold, cyan) and a membrane-bound Golgi glycoprotein (β4Gal-T1-HaloTag/JFX549, light yellow) over a 226 x 226 x 10 µm^3 volume. Scale bar, 20 µm. See Supplementary Video 4.

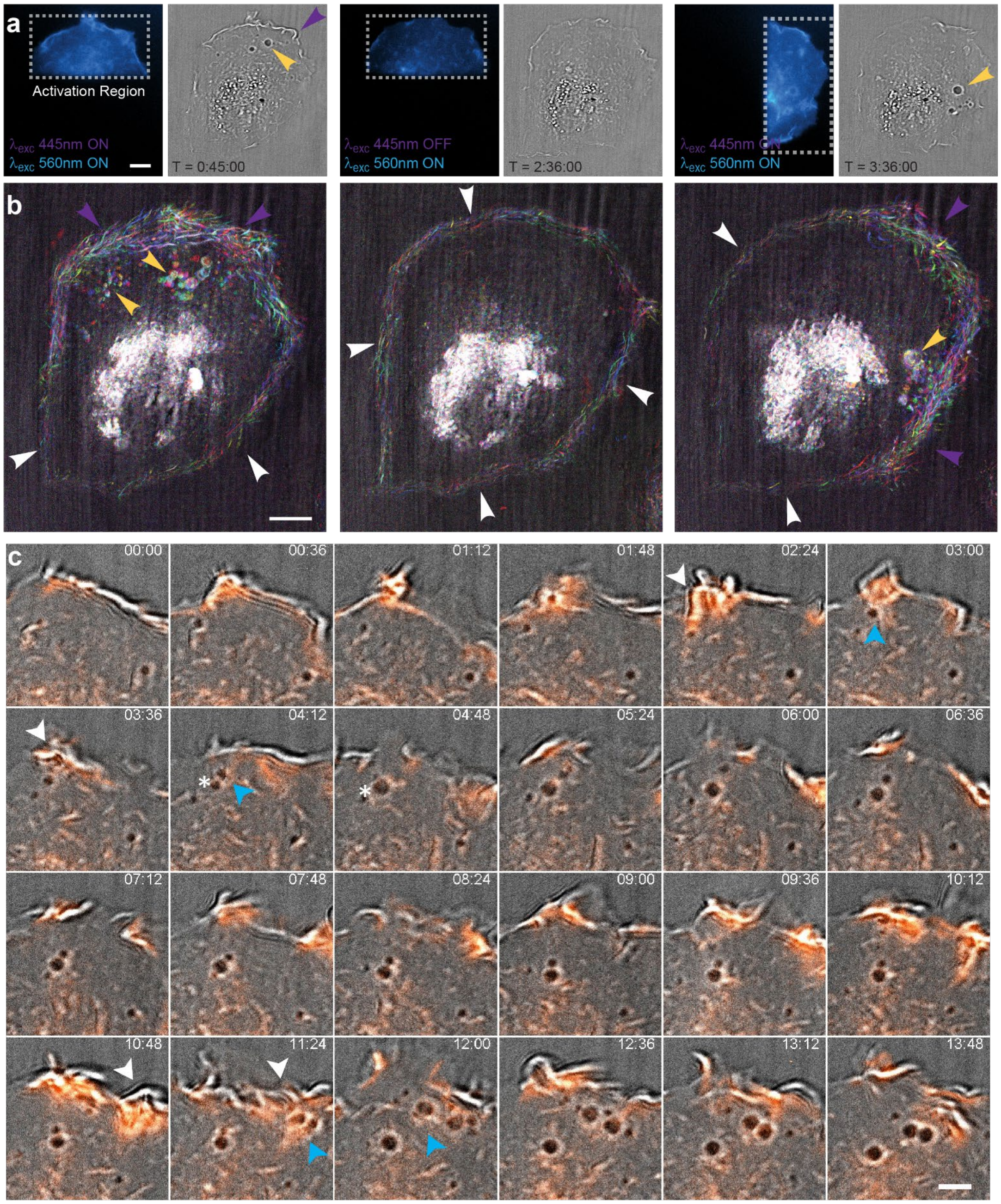

Supplementary Figure 13. Optogenetic induced macropinocytosis with correlative oblique illumination. (a) Localized optogenetic activation of PA-Rac1-mCherry (blue, left panel in each example) induces the formation of macropinosomes (yellow arrows) after membrane ruffling (purple arrow) as seen by OI. Scale bar, 10 µm. **(b)** Color-coded time projections highlight induced ruffling (purple arrows) and macropinosome formation (yellow arrows) within the photoactivated areas in (a). White arrows indicate background ruffling in non-activated regions. Scale bar, 10 µm. **(c)** Time series of macropinosome formation using interleaved photoactivation, fluorescence (orange), and OI (gray) modalities. White arrows indicate membrane ruffles which collapse to form macropinosomes indicated by blue arrows. Asterisk indicates a site of vesicle fusion. Scale bar, 5 µm. See Supplementary Video 5.

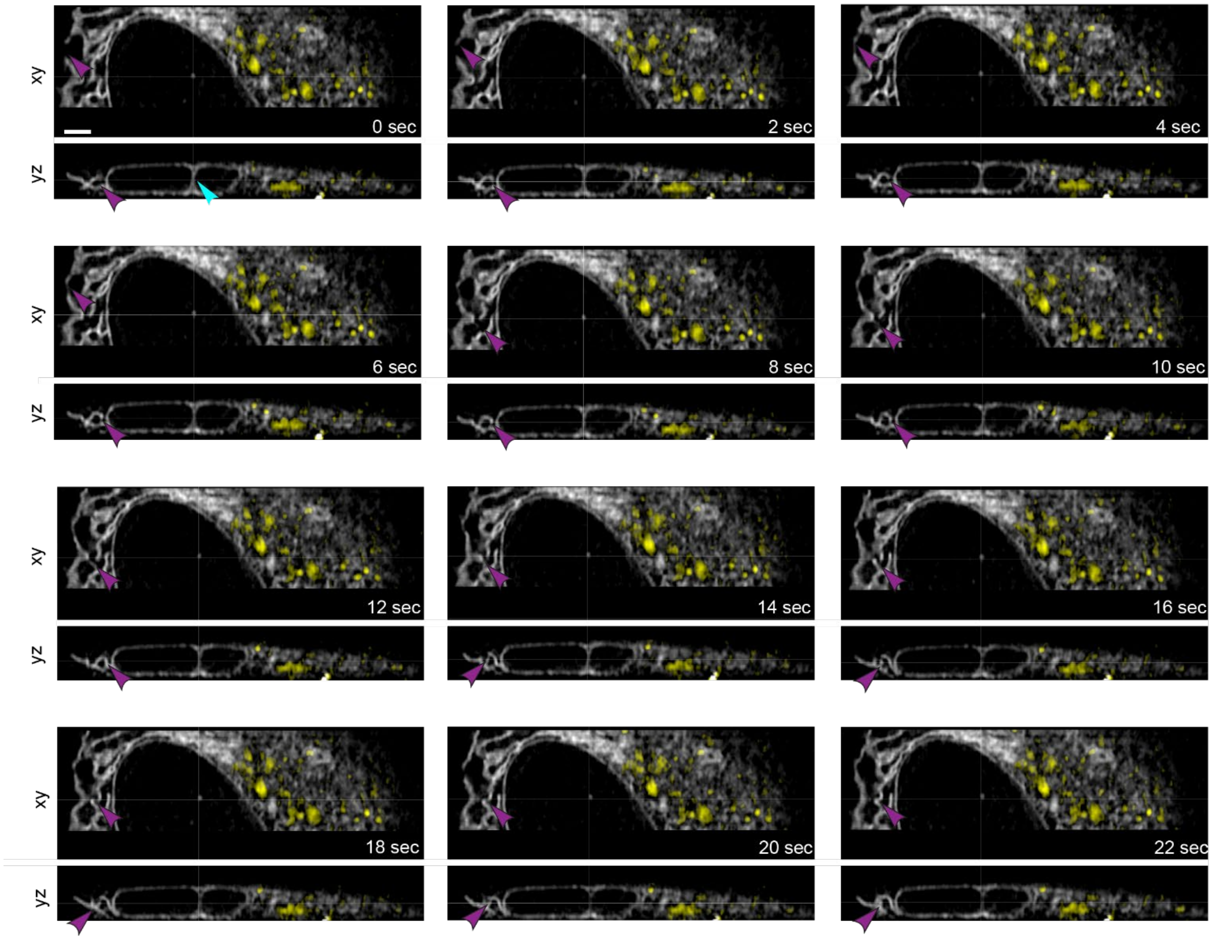

Supplementary Figure 14. Rapid 4D SR imaging of subcellular dynamics. Time-lapse movie of a live hTERT-RPE1 cell expressing ER (calreticulin-StayGold, gray) and Golgi (β4Gal-T1-HaloTag/JFX549, yellow) markers imaged every 2 seconds by LLS-SIM. Magenta arrows highlight dynamic ER remodeling events. Cyan arrows highlight an ER tunnel passing through the cell nucleus. Images show xy and yz orthoslices. Scale bar, 4 µm. See Supplementary Video 6.

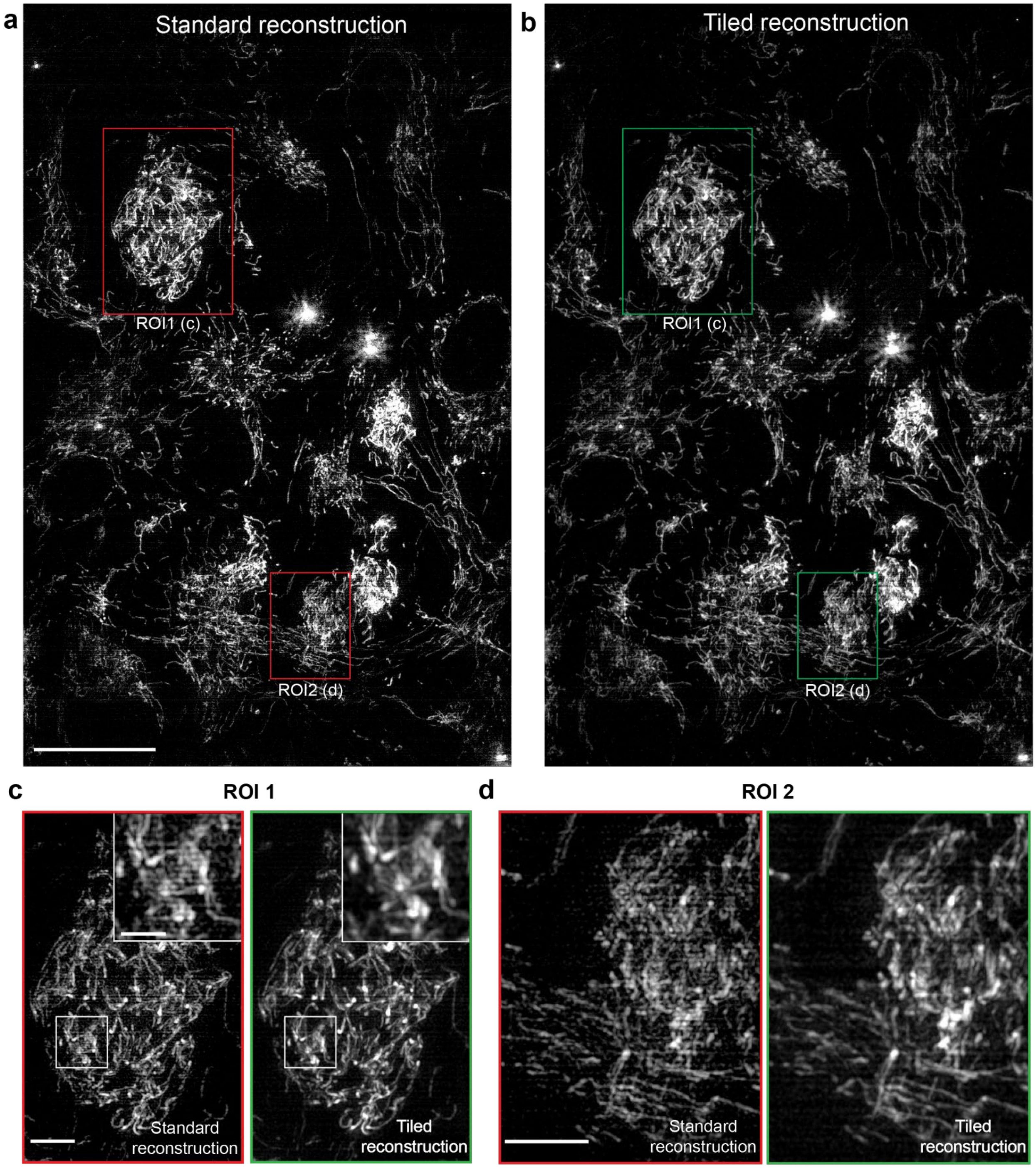

Supplementary Figure 15. Tiled SIM reconstruction reduces artifacts. (a) xy MIP from a single 3D-SIM reconstruction covering a large FOV of live hTERT-RPE1 cells expressing the mitochondria marker COX8a-StayGold. **(b)** xy MIP from a tiled 3D-SIM reconstruction of the same volume using a 128 x 128 pixel tiles across 40 z planes, each reconstructed independently, followed by stitching based on a 32 pixel overlap between adjacent tiles. Scale bar, 25 µm. **(c)** Comparison in ROI1 reveals residual grating artifacts for the standard reconstruction (left and left inset) not apparent with the tiled approach. Scale bars, 5 µm and 2 µm (inset). **(d)** Similar comparison for ROI2. See Supplementary Video 7.

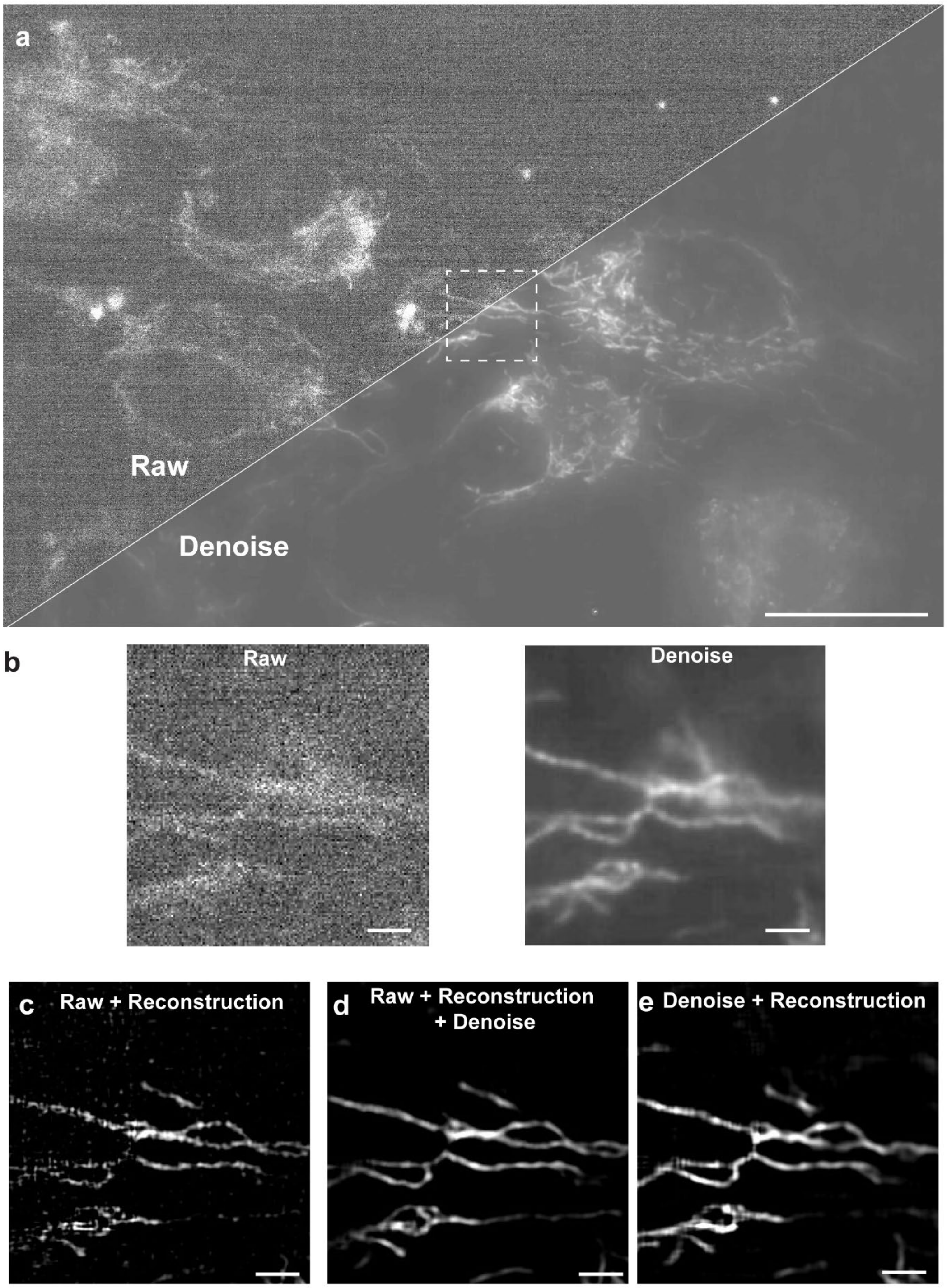

Supplementary Figure 16. Denoising strategies for reconstructing low SNR 3D-SIM data. (a) xy MIP of a 3D-SIM single raw image of live hTERT-RPE1 cells expressing mitochondria marker (COX8a-StayGold) without and with denoising[26] applied. Scale bar, 25 µm. **(b)** Zoomed view of the boxed region in (a), showing reduction of noise at right. **(c-e)** 3D SIM reconstruction of the region in (b) under three scenarios: (c) reconstruction with raw data; (d) denoising before reconstruction; and (e) denoising after reconstruction. Option (e) is preferred to prevent loss of high spatial frequencies when denoising before reconstruction. Scale bar for (b-e), 2 µm.

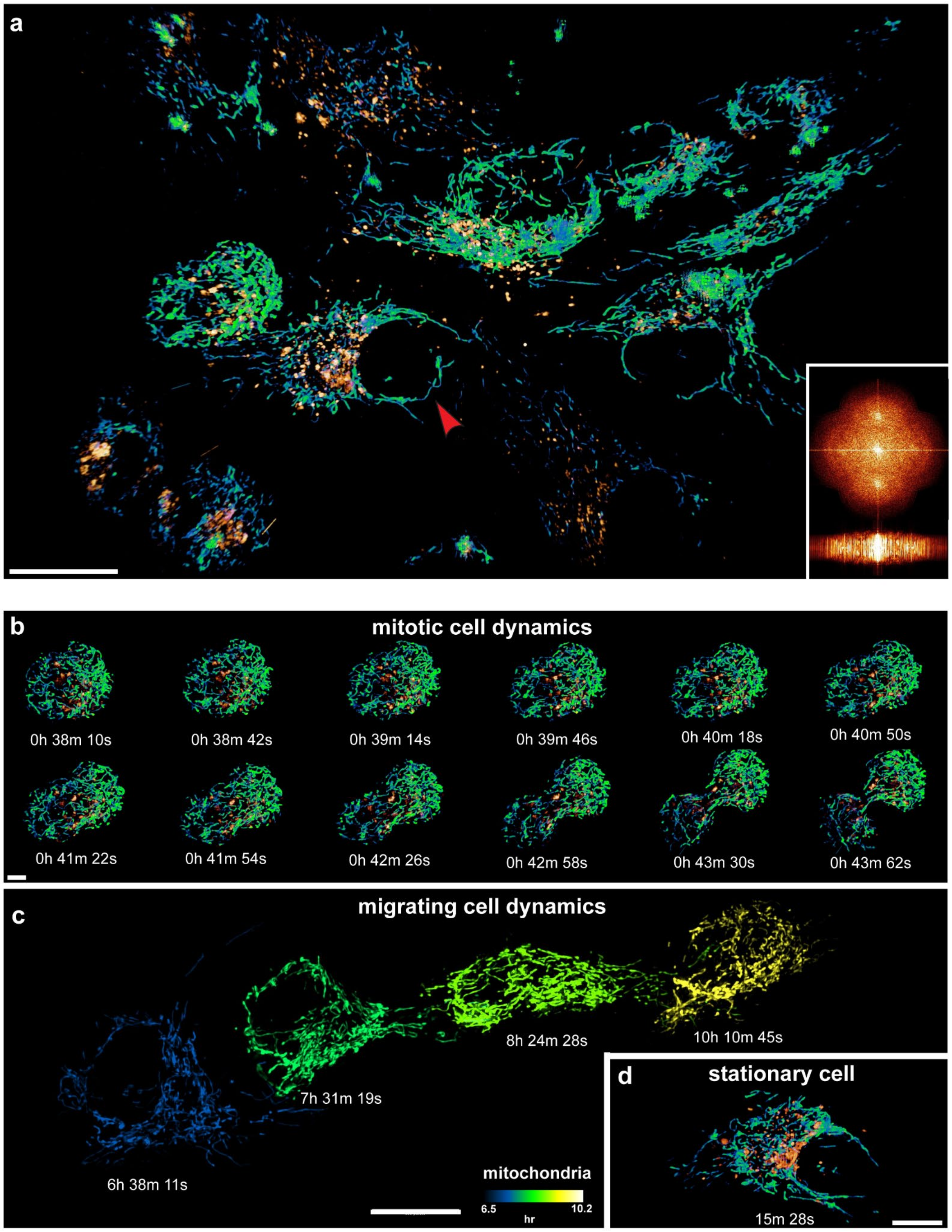

Supplementary Figure 17. Extended live-cell 3D-SIM time lapse imaging using optimized acquisition and denoising. Long-term (11 hours, 1200 time points) dual-color 3D-SIM imaging achieved by: i) using two rather three grating illumination orientations (0°and 90°) per z-plane; and ii) reduced laser power / exposure time, made possible by post-acquisition denoising. **(a)** xy MIP at one time point showing live hTERT-RPE1 cells co-expressing mitochondrial (COX8a-StayGold, green-blue) and Golgi (β4Gal-T1-HaloTag-JFX549, orange) markers. Inset: xy/yz OTFs from the mitochondria channel shows extension of the support. Scale bar, 20 µm. **(b)** A full cell division cycle recorded over 160 sec at 32 sec intervals. Scale bar, 5 µm. **(c)** Cell migration over ~3.5 hours. Scale bar, 15 µm. **(d)** A stationary cell, marked by a red arrowhead in (a). Scale bar, 10 µm. See Supplementary Video 7.

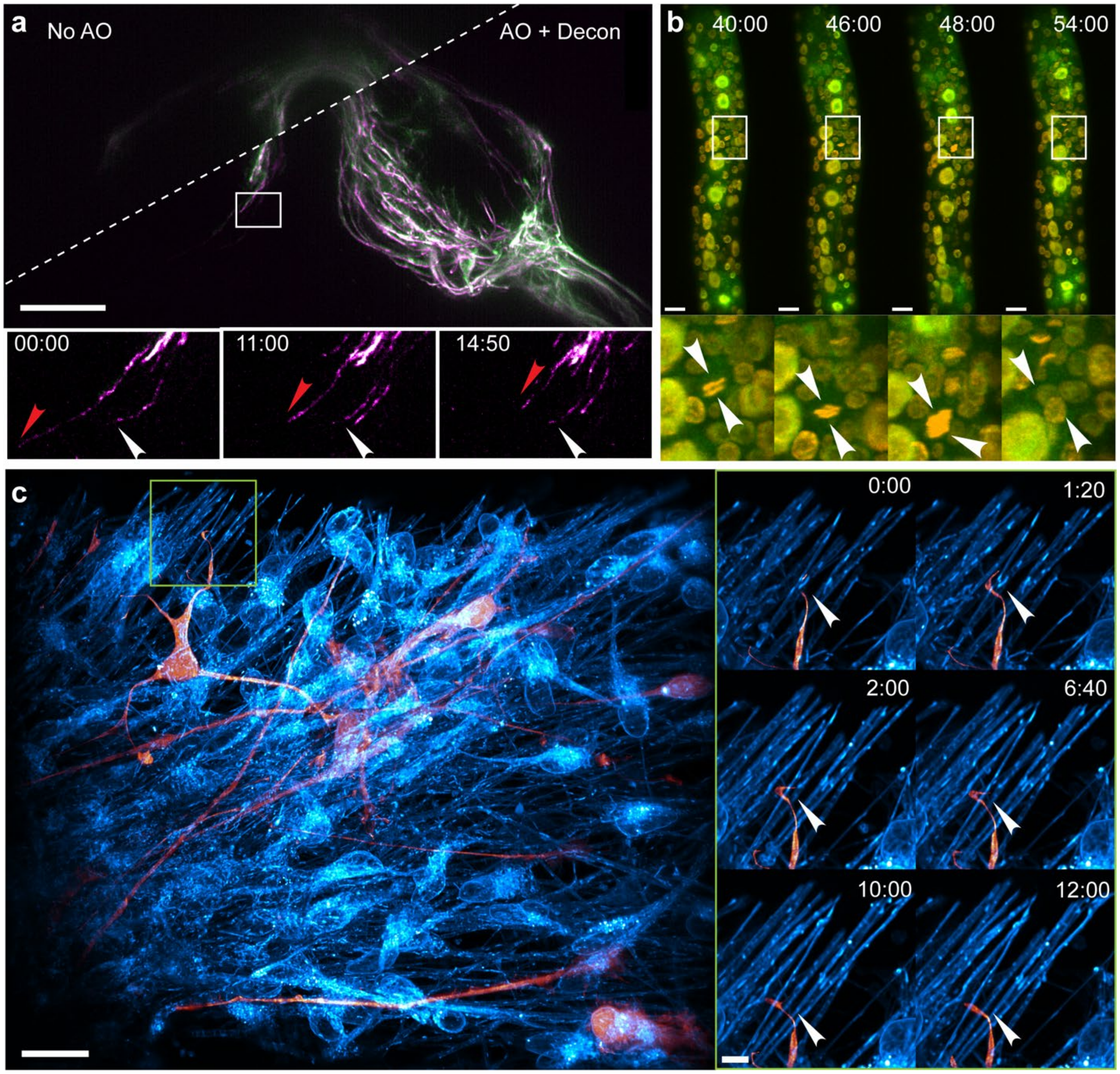

Supplementary Figure 18. Application of AO-LLSM to other model organisms. (a) *Drosophila* olfactory receptor neuron axons in the antenna lobe during the target selection process, showing axons expressing membrane marker (mCD8-GFP, magenta) and microtubule marker (Halo7-EB1, green) before (top left) and after AO (bottom right). Inset: Zoom-in view of the white box showing the microtubule-rich exploring branches dynamics after AO correction. White and red arrows highlighting dynamically extending and retracting branches, respectively. Scale bar, 20 µm. **(b)** Top: Time series showing an L3 stage *C. elegans* larva co-expressing a CDK1/2 activity sensor (green) and histone (orange). Bottom: Division (white arrows) of a single somatic gonad cell -- the CDK1/2 activity sensor is excluded from the nucleus during mitosis (40, 46, and 48 mins) but re-localizes in the daughter cells afterwards (54 mins). Scale bar, 10 µm. **(c)** Left: Neuronal and mitochondrial dynamics (Supplementary Video 13) in human iPSC-derived brain organoids with global mitochondria label mitotracker (cyan) and cytoplasmic-mGFP (orange) expressed in ~20% of all cells. Scale bar, 20 µm. Right: a Neuronal projection in the boxed region dynamically extending to probe its environment. Scale bar, 5 µm. Time stamps correspond to minutes:seconds.

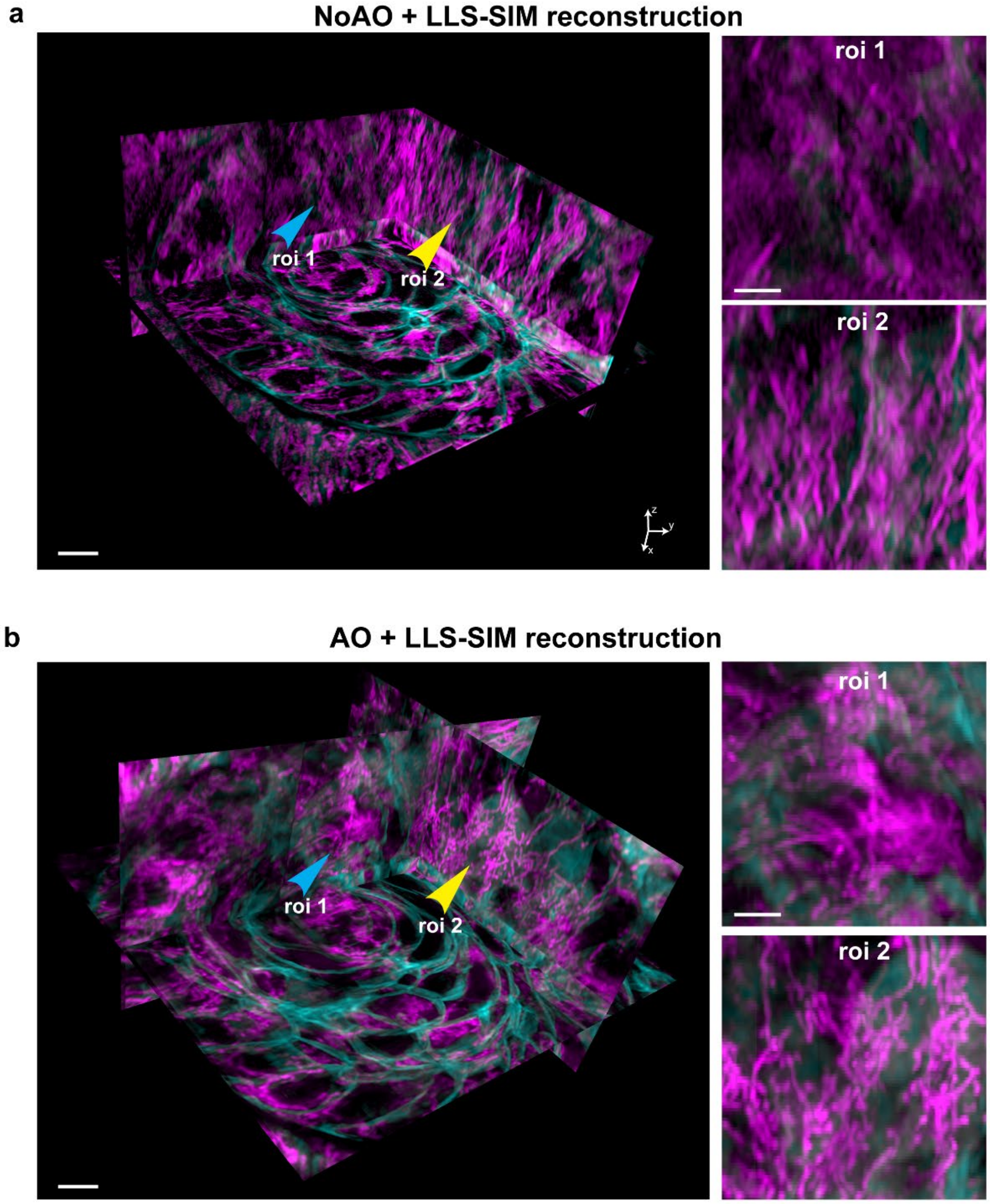

Supplementary Figure 19. AO correction is essential for accurate LLS-SIM reconstruction in multicellular environments. LLS-SIM reconstruction of mitochondria (magneta) and plasma membranes (cyan) without **(a)** and with **(b)** AO in the zebrafish eye, in the same region as Fig. 5b. Axial features (cyan and yellow arrows) are particularly distorted before correction. Scale bar, 5 µm (orthoslice view) and 2 µm (zoomed view).

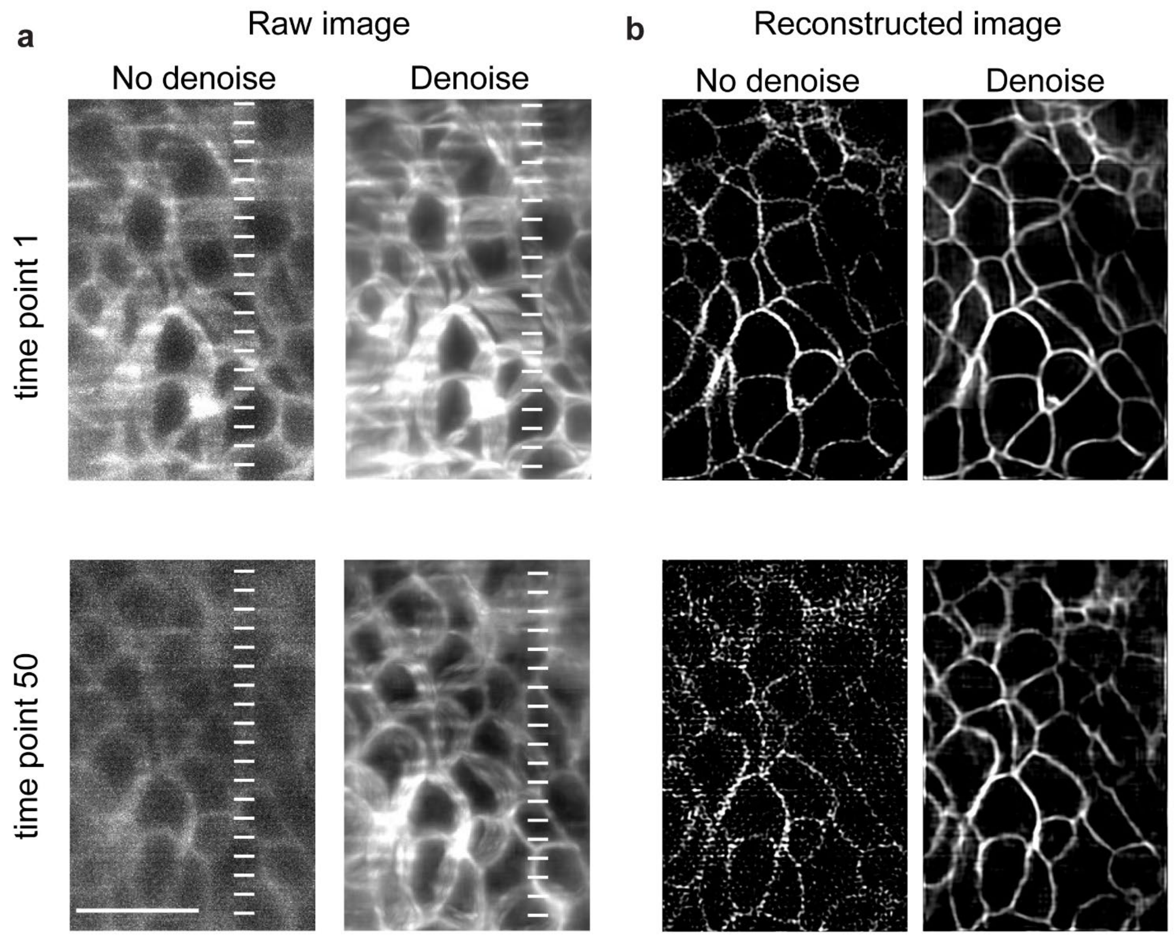

Supplementary Figure 20. Denoising improves reconstruction of low SNR time-lapse AO-LLS-SIM data. (a) 5 µm MIP slab of a single low SNR raw volume at a fixed phase of LLS-SIM excitation, before and after denoising. **(b)** Corresponding LLS-SIM reconstructions. Denoising extends the photon budget for the imaging and becomes critical for accurate reconstruction at later time points. Scale bar, 10 µm.

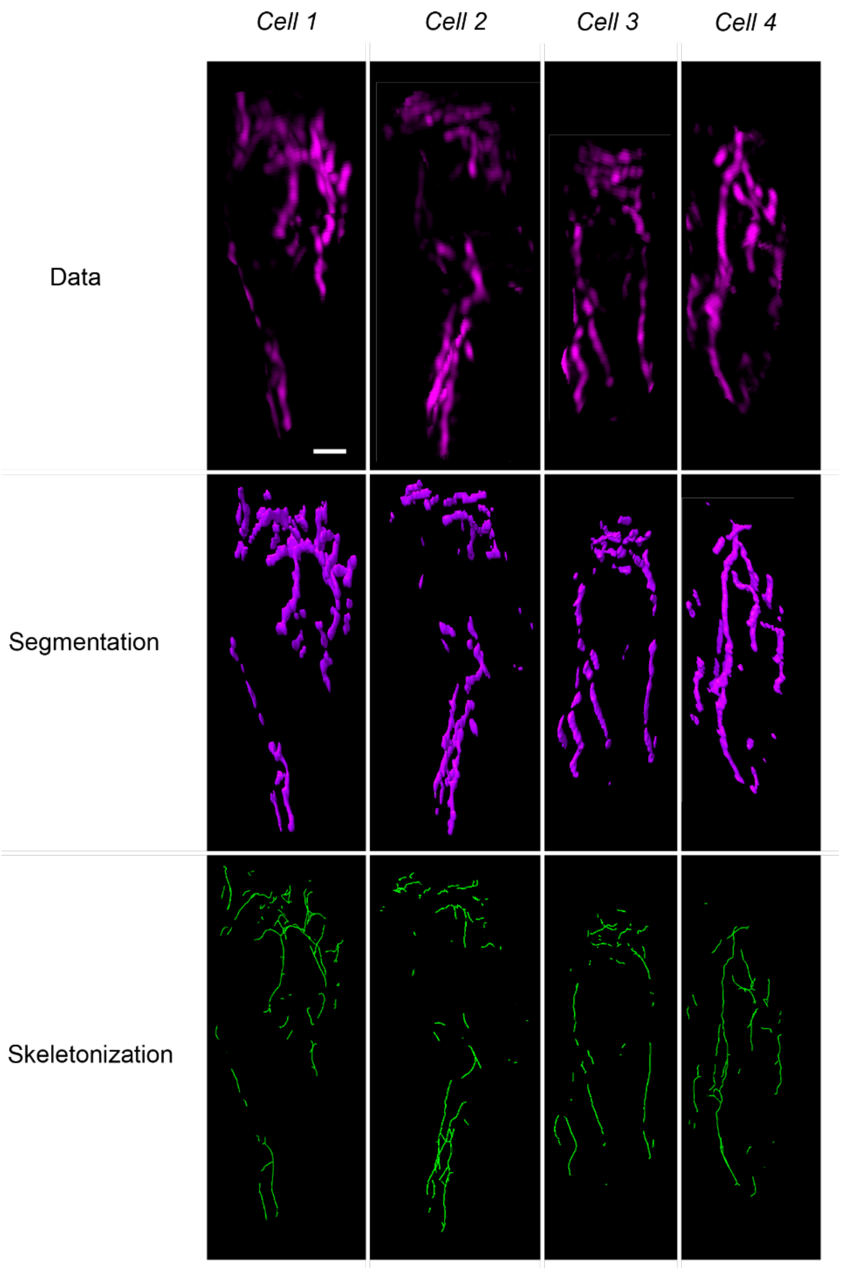

Supplementary Figure 21. Processing steps for analyzing mitochondrial network morphology. Four examples that illustrate the analysis pipeline applied to mitochondria within a single cell segmented from zebrafish tissue. Left: Raw fluorescence imaging data (magenta). Middle: 3D segmentation identifying mitochondrial voxels. Right: Skeletonization representing the mitochondrial medial axis (green). Scale bar, 2 µm.

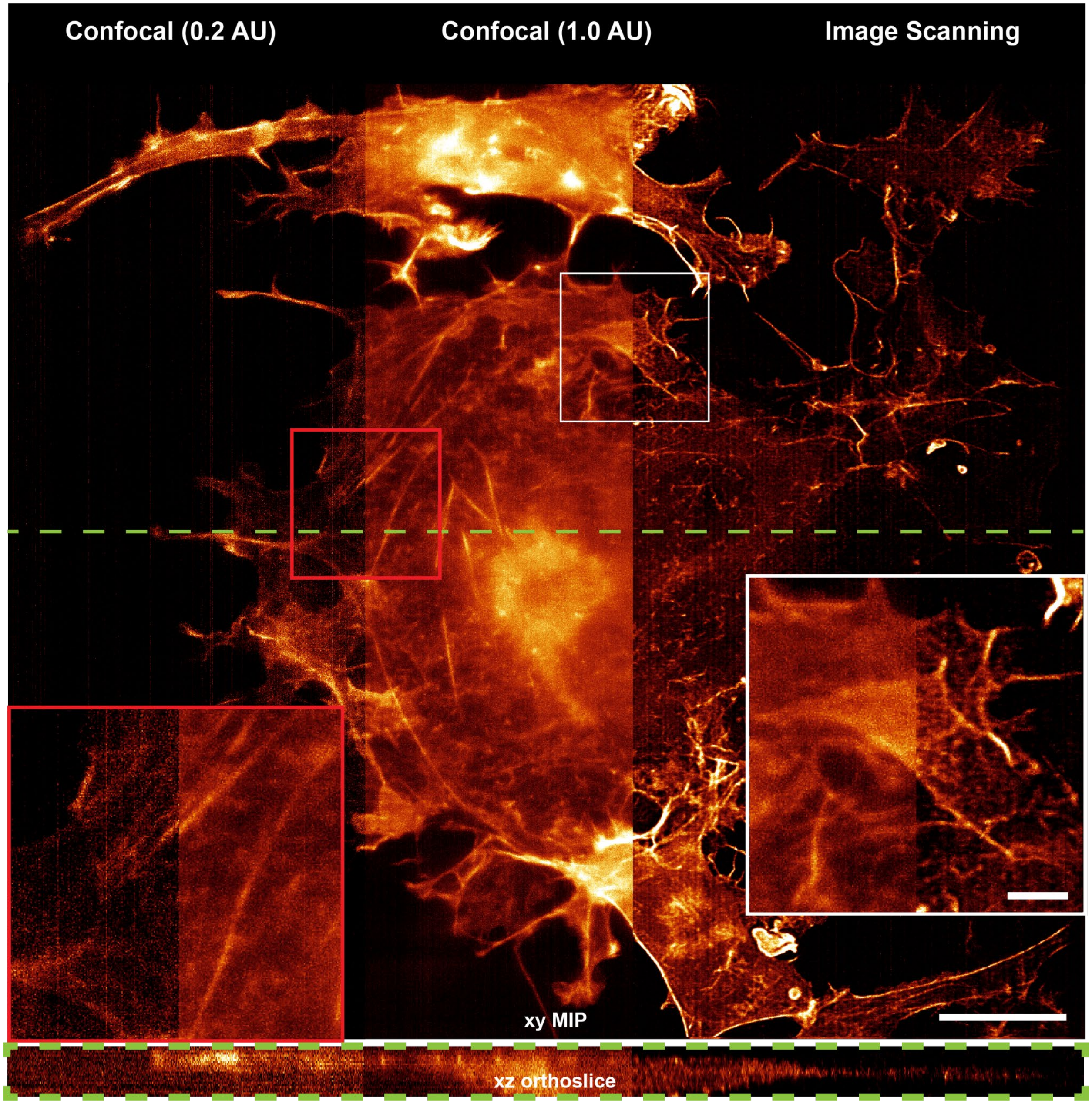

Supplementary Figure 22. Comparison of linescan confocal and image scanning microscopy (ISM). 3D imaging of COS-7 cells expressing the actin marker LifeAct-mCherry shows ISM resolution comparable to linescan confocal imaging with a tightly closed collection slit (0.2 Airy Unit, AU width) while maintaining the higher SNR typical of a larger slit (1.0 AU). Only the actin channel is shown here. See Supplementary Video 15 for two-color (actin and microtubule) imaging. Scale bar, 10 µm.

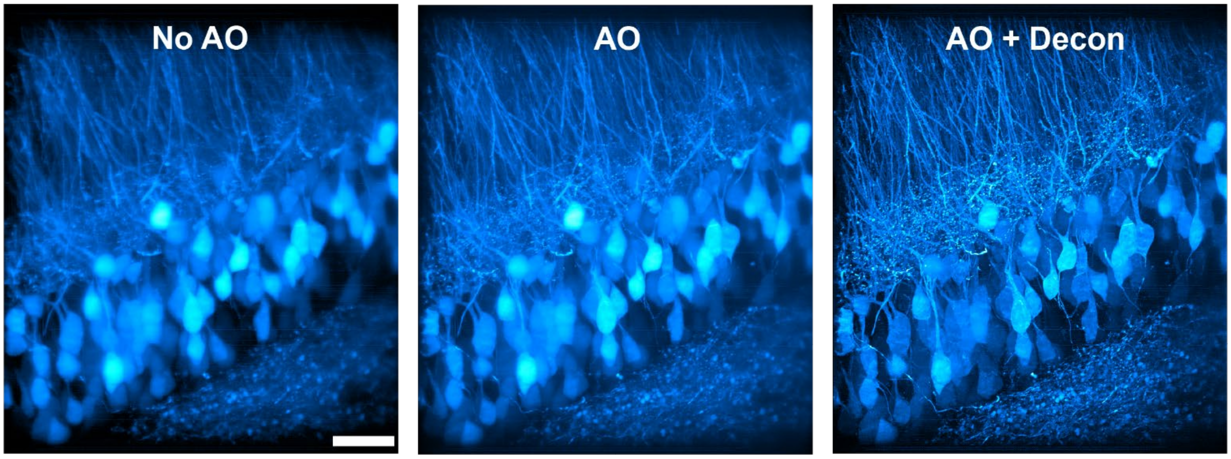

Supplementary Figure 23. **AO improvement for two-photon Bessel light sheet imaging in thick brain slices**. Comparison of images from a 300-µm thick acute brain slice (3-month-old Thy1-GFP-M mouse) acquired using a two-photon Bessel light sheet (NA = 0.55/0.58) without AO correction (left), with AO correction (middle), and with AO correction plus deconvolution (right). Scale bar, 20 µm.

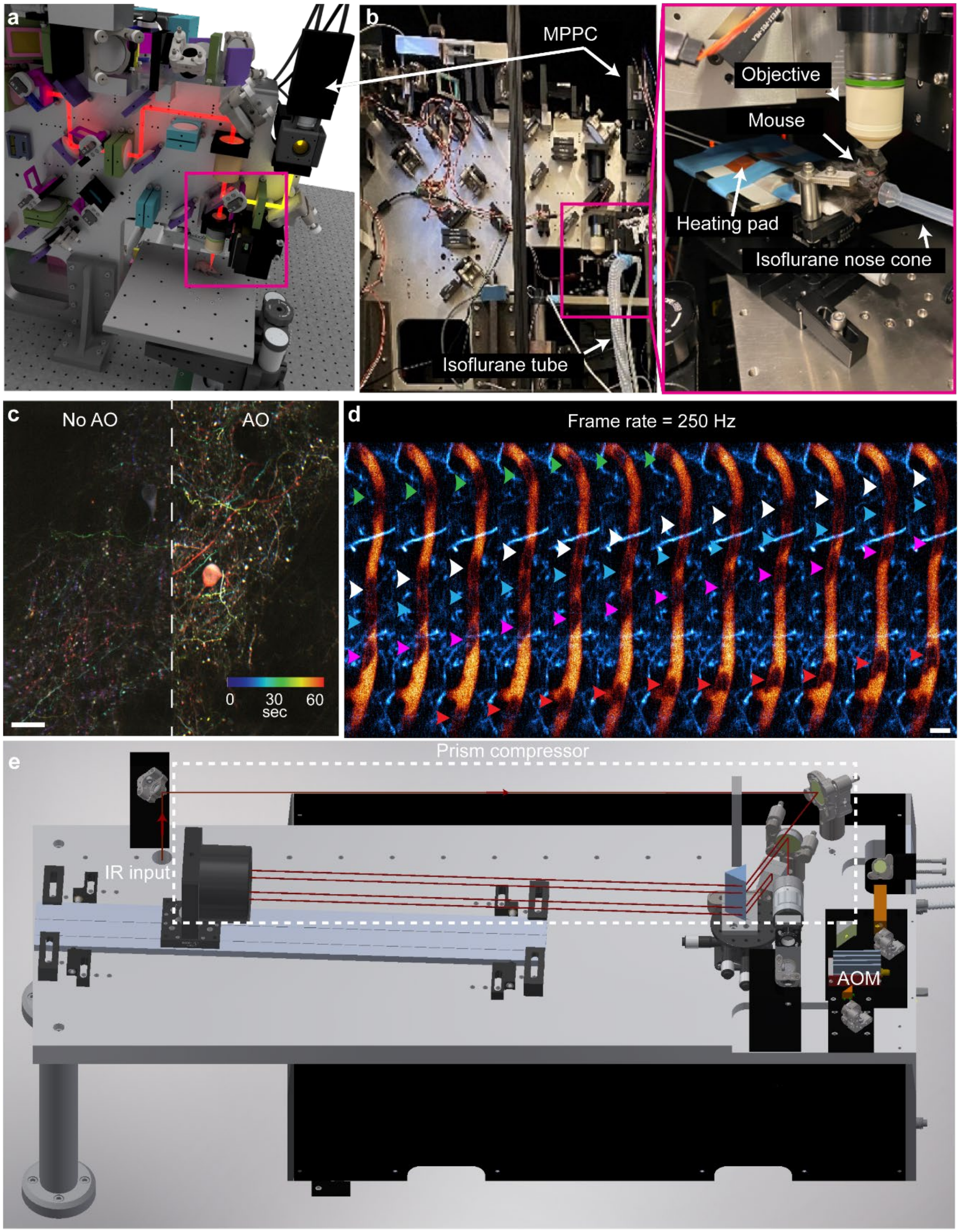

Supplementary Figure 24. AO-TPM enables deep functional imaging in live mice. (a) 3D model of the upright MOSAIC work area for *in vivo* AO-TPM in mice. **(b)** Corresponding photograph with magnified view of a mouse in place for imaging. **(c)** Improved SNR for detection of GCaMP7s transients with (right) and without (left) AO correction. Scale bar, 20 µm. **(d)** Erythrocyte transport in a capillary imaged at 250 Hz using resonant scanning AO-TPM. Scale bar, 5 µm. **(e)** 3D model shows the TP femtosecond laser beam path, including the prism compressor for group velocity dispersion compensation and the acousto-optic modulator for output power control.

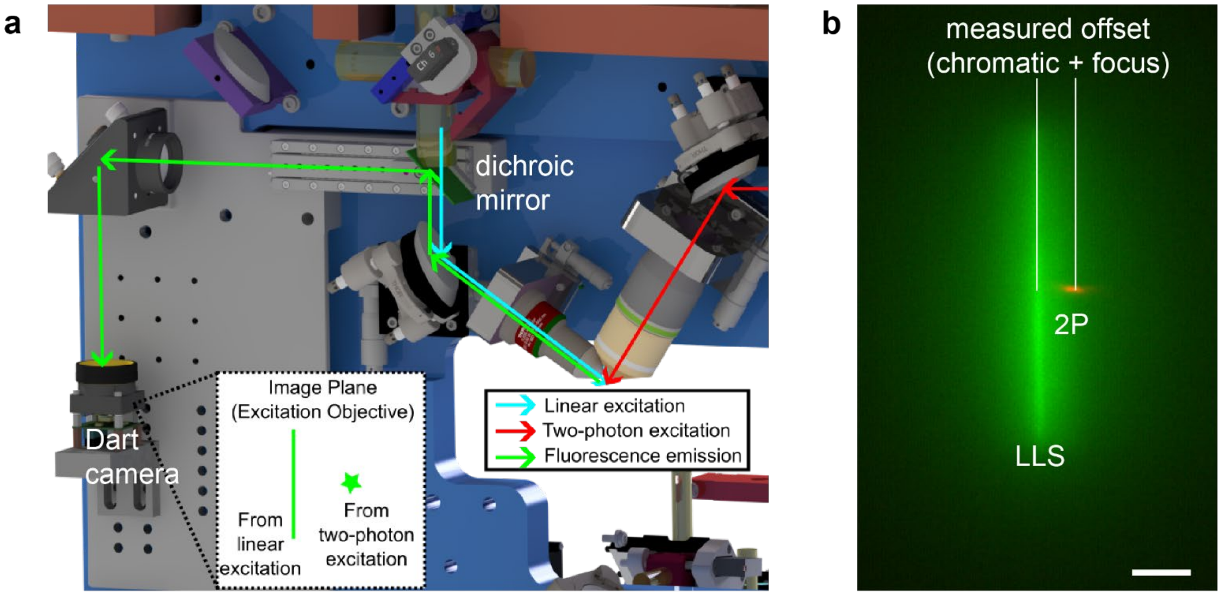

Supplementary Figure 25. Autofocus (AF) module for continuous axial alignment of light sheet to detection focal plane. (a) 3D model and optical path of the AF module design integrated into MOSAIC. **(b)** Image from the AF detection camera showing overlaid fluorescence signals generated simultaneously by the LLS (green) from the excitation objective and TPE focus (red) from the detection one. Scale bar, 10 µm.

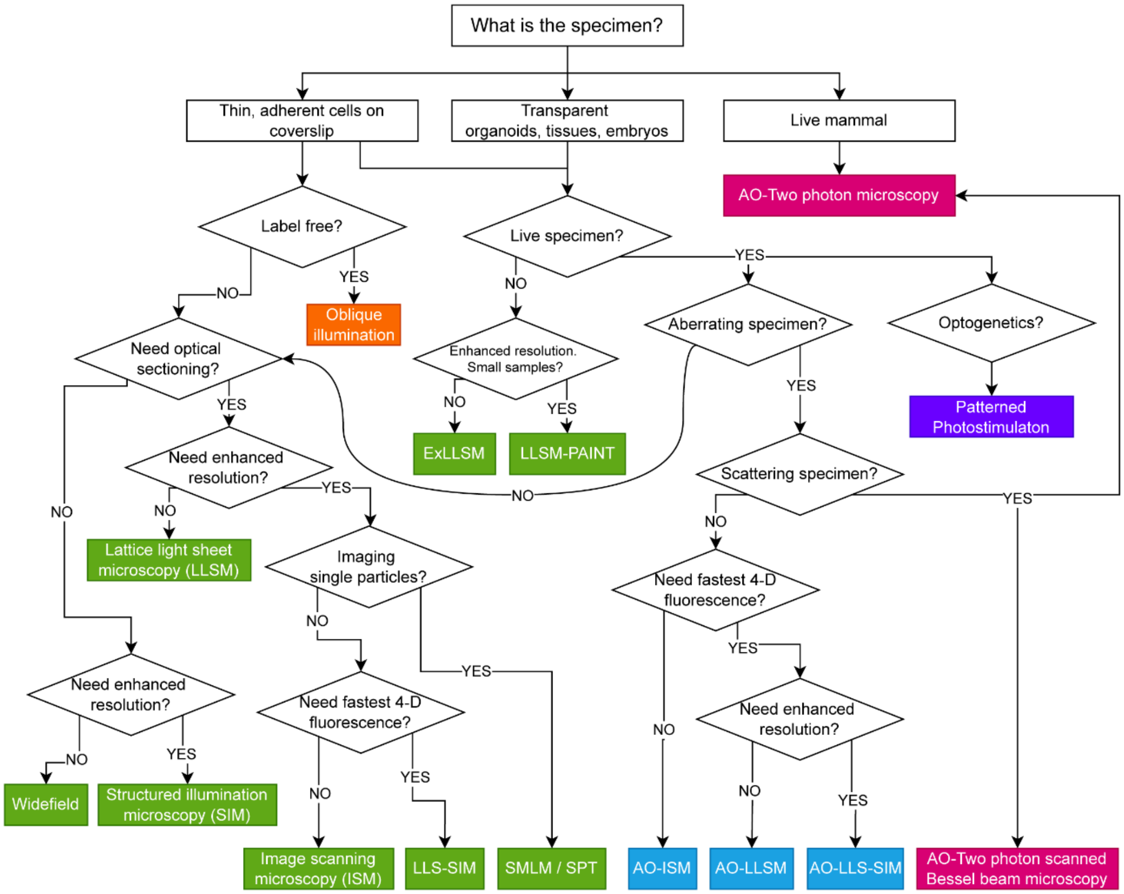

Supplementary Figure 26. Decision tree for MOSAIC modality selection. The flowchart summarizes native imaging context, options for capability-extending (resolution and speed), and interleaved correlative combinations.

Supplementary Table 1. Imaging conditions for all experiments.

			**Sample**		**Imaging Conditions**								**Image Processing**	
**Related Figure, Movie #**	**Imaging Modality**	**Adaptive Optics**	**Sample Name**	**Fluorescent Label**	**Tempera ture**	**Excitation Light Description**	**Voxel Size (dx, dy, dz/ds) (nm)**	**Imaged Volume (x,y,z) (µm)**	**Time Interval x Total Time Points**	**Imaging Channels (excitation (nm); exposure time; power and place of measurement)**	**Stage Movement**	**Dichroic and Filter**	**Image Preprocessing**	**Image Analysis & Visualization**
**1a, b; Supple mentar y Video 2**	**LLS**	No	LLC-PK1 cells	ER-mEmerald-Calnexin H2B-mCherry	37 °C	HB-Hex, NA_{exc} = 0.35, σ_{NA} = 0.09, ϵ = 0.010	108 x 108 x 161	1000 x 750 x 10	90 sec x 989	488: 3 ms, 5.9 µW<br>560: 3 ms, 8.4 µW	Sample scan (continuous sweep)	R561; CamA: FF01-509/22 CamB: FF01-600/37	Flat-field correction, stitching, deconvolution, deskewing and rotation, unmixing	Imaris, ImageJ, Agave
**1c; Supple mentar y Video 3**	**Oblique illumination**	No	Hela cells	NA	37 °C	642 nm oblique light sheet	108 x 108	200 x 200	1 sec x 1000	642: 10ms, 2.2 µW	NA	NA	Flat-field correction	ImageJ
**1d; Supple mentar y Video 3**	**Oblique illumination**	No	U2OS cells	NA	37 °C	642 nm oblique light sheet	108 x 108	1218 x 975	1 sec x 1,000	642: 10ms, 2.2 µW	NA	NA	Flat-field correction, stitching	Imaris, ImageJ
**2a**	**LLS-SPT**	No	mESC cells	SOX2-HT9-PA-JF646	37 °C	MB-Square, NA_{max}=0.4, NA_{min}=0.3	110 x 110	28.6 x 88	20 ms x 20,000	405: 20 ms, 26 µW<br>514: 100ms, 0.17 mW<br>642: 20ms, 14.5 mW	NA	R635; CamA: BLP01-514R, NF03-642E CamB: BLP01-647R	NA	ImageJ, Python, Trackmate
**2b; Supple mentar y Video 6**	**LLS-SIM**	No	hTERT-RPE1 cells	ER-StayGold β4G-HT9-JFX549	37 °C	HB-Hex, NA_{exc} = 0.4 (488 nm) / 0.46 (560 nm), σ_{NA} = 0.08, ϵ = 0.010	49 x 49 x 188	50 x 188 x 10	10 sec x 1,500	488: 3 ms, 44 µW<br>560: 3 ms, 35 µW	Sample scan (step and settle)	R561; CamA: FF01-509/22, CamB: FF01-600/37	Deskewing and rotation, SIM reconstruction	Imaris, Agave
**2c, S15; Supple mentar y Video 7**	**3D-SIM**	No	hTERT-RPE1 cells	COX8a-StayGold β4G-HT9-JFX549	37 °C	NA_{exc} = 0.85, 3-orientation (0°, 60°,120°)	49 x 49 x 250	100 x 157 x 9.3	57 sec x 156	488: 20 ms, 403 µW<br>560: 20 ms, 80 µW	Objective scan	R561; CamA: FF01-509/22 CamB: FF01-600/37	SIM reconstruction	Imaris, ImageJ, Agave
**2e, f; Supple mentar y Video 8**	**Correlated oblique illumination, widefield, LLS, LLS-SIM, and 3D-SIM**	No	hTERT-RPE1 cells	COX8a-StayGold β4G-HT9-JFX549	37 °C	**OI**: 607 nm oblique light sheet<br>**Widefield**: 488 and 560 nm<br>**LLS/LLS-SIM**: HB-Hex, NA_{exc} = 0.4 (488 nm) / 0.46 (560 nm), σ_{NA} = 0.08, ϵ = 0.010<br>**3D-SIM**: NA_{exc} = 0.85, 3-orientation (0°, 60°,120°)	**OI**: 98 x 98;<br>**LLS/LLS-SIM**: 98 x 98 x 188;<br>**Widefield/3D-SIM**: 49 x 49x 250	100 x 157 x 10	3 min x 20	**OI**: 607: 1 ms, 3.4 µW<br>**Widefield**: 488: 20 ms, 504 µW; 560: 20 ms. 86 µW<br>**LLS/LLS-SIM**: 488: 3 ms, 252 µW; 560: 3 ms, 50 µW<br>**3D-SIM**: 488: 20 ms, 504 µW; 560: 20 ms, 86 µW	**LLS/LLS-SIM**: Sample scan<br>**Widefield/3D-SIM**: Objective scan	R561; FF01-509/22, FF01-600/37	Multimodal image registration;<br>**OI**: flat-field correction;<br>**LLS**: deconvolution, deskewing and rotation;<br>**LLS-SIM**: SIM reconstruction, deskewing, and rotation;<br>**3D-SIM**: SIM reconstruction	Imaris, ImageJ
**3a; Supple mentar y Video 9**	**LLS-PAINT**	No	U2OS	Mitochondria-TOMM-20 Nuclear envelope-Lamin A/C	Room	MB-Square, NA_{max}=0.4, NA_{min}=0.3	108 x 108 x 358	180 x 200 x 17	NA	560: 60 ms, 30 mW<br>642: 50 ms, 38 mW	Sample scan (step and settle)	T600dcrb; FF01-600/37, FF01-685/40	Single-molecule fitting, deskewing and rotation	Imaris

**3b-f; Supplementary Video 10**	**ExLLSM**	No	Human hippocampal tissue	NF200-AT 647N MBP-AF 568	Room	HB-HexRect, NA_{exc} = 0.25, σ_{NA} = 0.08, ϵ = 0.010	108 x 108 x 268 (post-expansion) 27 x 27 x 67 (pre-expansion)	8,000 x 9,500 x 390 post-expansion (2,000 x 2,375 x 98 pre-expansion)	NA	560: 3.8 ms, 1680 μW 642: 3.8 ms, 302 μW	Sample scan (continuous sweep)	T600dcrb; FF01-600/37, FF01-685/40	Flat-field correction, stitching, deconvolution, deskewing and rotation, puncta removal	Imaris
**4a,b**	**LLS**	Yes	Zebrafish (48 hpf)	Fish: Tg(kdrl:GFP) Cancer cell: Lifeact-mRuby	33 °C	MB-Square, NA_{max}=0.4, NA_{min}=0.38, Crop Factor=6	108 x 108 x 200	55 x 183 x 50	64 sec x 200	488: 20ms 560: 20ms	Objective scan	T600dcrb; FF01-600/37, FF01-538/685	Deconvolution	Imaris, Agave
**4c-d; Supplementary Video 11**	**LLS**	Yes	Zebrafish (48 hpf)	Membrane-mNeonGreen Nuclei-H2B-miRFP670	Room	MB-Square, NA_{max}=0.4, NA_{min}=0.38, Crop Factor=6	108 x 108 x 200	216 x 272 x 37 (comprising 1 x 2 x 2 tiles)	3 min x 250	488: 50ms 560: 50ms	Sample scan	T600dcrb; FF01-600/37, FF01-538/685	Stitching, deconvolution, deskewing and rotation	Imaris
**4e; Supplementary Video 12**	**LLS**	Yes	Zebrafish (16 hpf)	Membrane-mNeonGreen DHB-mScarlet Histone-miRFP670	Room	MB-Square, NA_{max}=0.4, NA_{min}=0.38, Crop Factor=6	108 x 108 x 200	216 x 173 x 37 (comprising 1 x 1 x 4 tiles)	NA	488: 50ms 560: 50ms 642: 50ms	Sample scan	T600dcrb; FF01-600/37, FF01-538/685	Stitching, deconvolution, deskewing and rotation	Amira
**5a, b, S19; Supplementary Video 14**	**LLS-SIM**	Yes	Zebrafish (14 hpf)	Mitochondria-StayGold Membrane-mChilada	Room	HB-Hex NA_{exc} = 0.4 (488 nm) / 0.46 (560 nm), σ_{NA} = 0.08, ϵ = 0.010	108 x 108 x 180	61 x 57 x 40	NA	488: 100 ms, 252 μW 560: 100 ms, 34 μW	Objective scan	R561; FF01-509/22, FF01-600/37	Denoising, SIM reconstruction, stitching	Imaris, Agave
**5c-e, S20, S21; Supplementary Video 15**	**LLS-SIM**	Yes	Zebrafish (14 hpf)	Mitochondria-StayGold Membrane-mChilada	Room	HB-Hex NA_{exc} = 0.4 (488 nm) / 0.46 (560 nm), σ_{NA} = 0.08, ϵ = 0.010	108 x 108 x 180	56 x 56 x 40	93 sec x 60	488: 5.3 ms, 168 μW 560: 5.3 ms, 84 μW	Objective scan	R561; FF01-509/22, FF01-600/37	Denoising, SIM reconstruction, stitching	Imaris, Agave
**5f; Supplementary Video 16**	**ISM**	Yes	Zebrafish (7 dpf)	Membrane-mNeonGreen	28.5 °C	Line focus NA = 1.0	100 x 100 x 350	336 x 319 x 84	NA	488: 5ms/line (8 sec/plane)	Objective scan	FF01-509/22	Stitching, flat-field correction, ISM reconstruction	Imaris
**5g-I; Supplementary Video 16**	**ISM**	Yes	Zebrafish (7 dpf)	Membrane-mNeonGreen	28.5 °C	Line focus NA = 1.0	100 x 100 x 350	354 x 332 x 16.3	32 min x 13	488: 2ms/line (2 sec/plane)	Objective scan	FF01-509/22	Stitching, flat-field correction, ISM reconstruction	Imaris
**6a-c; Supplementary Video 17**	**TPE**	Yes	Mouse	cytosolic-YFP	Mouse was kept on a heating pad	Confocal NA=1.0	100 x 100 x 500	100 x 100 x 400	NA	920: 0.064 ms / voxel, 120 mW	Objective scan	FF01-509/22	Deconvolution	Imaris, ImageJ
**6d, S24c; Supplementary Video 18**	**TPE**	Yes	Mouse	GCaMP7s	Mouse was kept on a heating pad	Confocal NA=1.0	200 x 200	200 x 200	62ms x 2002	920: 0.064 ms / voxel, 120 mW	NA	FF01-509/22	NA	MATLAB, ImageJ

**6f; Supplementary Video 19**	**TPE**	Yes	Mouse	cytosolic-YFP Blood vessel-TexasRed	Mouse was kept on a heating pad	Confocal NA=1.0	100 x 100 x 500	483 x 492 x 58 (comprising 5 x 5 x 1 tiles)	NA	920: 0.064 ms / voxel, 120 mW	Objective scan (axial) + Stage scan (lateral)	FF01-509/22	Deconvolution	ImageJ
**S24d; Supplementary Video 20**	**TPE**	Yes	Mouse	cytosolic-YFP Blood vessel-TexasRed	Mouse was kept on a heating pad	Confocal NA=1.0	200 x 200	83 x 13	NA	920 nm: 0.064 ms / voxel, 120 mW	Objective scan (axial) + Stage scan (lateral)	FF01-509/22	Deconvolution	ImageJ
**S11; Supplementary Video 4**	**Oblique illumination, LLS**	No	hTERT-RPE1 cells	COX8a-StayGold	37 °C	**OI**: 514 nm oblique light sheet<br>**LLS**: HB-Hex, NA_{exc} = 0.4 (488 nm) / 0.46 (560 nm), σ_{NA} = 0.08, ϵ = 0.010	**OI**: 98 x 98<br>**LLS**: 98 x 98 x 268	**OI**: 226 x 226<br>**LLS**: 107 x 156	57 sec x 152	488: 20ms<br>514 nm: 50ms	Sample scan (continuous sweep)	R561; FF01-509/22	**OI:** flat-field correction;<br>**LLS**: deconvolution, deskewing and rotation;<br>Multimodal image registration	ImageJ
**S12; Supplementary Video 4**	**Oblique illumination, LLS**	No	hTERT-RPE1 cells	ER-StayGold β4G -HT9-JFX549	37 °C	**OI**: 607 nm oblique light sheet<br>**LLS**: HB-Hex, NA_{exc} = 0.4 (488 nm) / 0.46 (560 nm), σ_{NA} = 0.08, ϵ = 0.010	**OI**: 98 x 98<br>**LLS**: 98 x 98 x 188	226 x 226 x 10	16.7 sec x 2,000	**OI**: 607: 0.6 ms, 2 μW<br>**LLS**: 488: 3 ms, 100 μW; 560: 3 ms, 34 μW	Sample scan (continuous sweep)	R561; FF01-509/22, FF01-600/37	Multimodal image registration;<br>**OI:** flat-field correction;<br>**LLS**: deconvolution, deskewing and rotation;	ImageJ
**S13; Supplementary Video 5**	**Photostimulation, oblique illumination**	No	hTERT-RPE1 cells	PA-Rac1-mCherry	37 °C	**OI**: 514 nm oblique light sheet<br>**Photostimulation**: 445nm point scanning	**OI**: 98 x 98<br>**Photostimulation**: 98 x 98	**OI**: 226 x 226<br>**Photostimulation**: 226 x 226	30 sec x 1188	445: 4000ms (for ON segments; disabled for OFF segments)<br>514: 50ms<br>560: 4000ms	NA	R561; FF01-509/22	**OI:** flat-field correction	ImageJ
**S14; Supplementary Video 6**	**LLS-SIM**	No	hTERT-RPE1 cells	ER-StayGold β4G-HT9-JFX549	37 °C	HB-Hex, NA_{exc} = 0.4 (488 nm) / 0.46 (560 nm), σ_{NA} = 0.08, ϵ = 0.010	49 x 49 x 188	11 x 188 x 10	2 sec x 1,500	488: 3 ms, 67 μW<br>560: 3 ms, 50 μW	Sample scan (step and settle)	R561; FF01-509/22, FF01-600/37	Deskewing and rotation, SIM reconstruction	Imaris
**S16, S17; Supplementary Video 7**	**3D-SIM**	No	hTERT-RPE1 cells	COX8a-StayGold β4G-HT9-JFX549	37 °C	NA_{exc} = 0.85, 2-orientation (0º, 90º)	49 x 49 x 250	100 x 157 x 9	32 sec x 1,500	488: 20 ms, 129 μW<br>560: 20 ms, 70 μW	Objective scan	R561; FF01-509/22, FF01-600/37	Denoising, SIM reconstruction	Imaris
**S18a**	LLS	Yes	Drosophila	mCD8-GFP Halo-JF646-EB1	Room	MB-Square, NA_{max}=0.4, NA_{min}=0.38, Crop Factor=6	100 x 108 x 250	76 x 151 x 60	18.2 sec x 30	488: 50 ms<br>642: 50 ms	Objective scan	R561; FF01-509/22, FF01-600/37	Deconvolution, deskewing and rotation	ImageJ
**S18b**	LLS	Yes	C. Elegans	CDK1/2-GFP H2B-mKate2	Room	MB-Square, NA_{max}=0.4, NA_{min}=0.38, Crop Factor=6	108 x 108 x 250	93 x 275 x 87	8.4 sec x 91	488: 20 ms<br>642: 20 ms	Objective scan	R561; FF01-509/22, FF01-600/37	Deconvolution	ImageJ
**S18c; Supplementary Video 14**	LLS	Yes	Brain organoid	Mitochondria-MitoTracker Cytosolic-eGFP	37 °C	MB-Square, NA_{max}=0.4, NA_{min}=0.3	108 x 108 x 215	221 x 200 x 30	40.5 sec x 21	488: 30 ms, 34 μW<br>642: 30 ms, 13.4 μW	Sample scan (continuous sweep)	R561; FF01-509/22, FF01-685/40	Deconvolution, deskewing and rotation	ImageJ, Imaris

**S22; Supplementary Video 15**	**ISM**	No	COS-7 cells	Tub1A-eGFP LifeAct-mCherry	37 °C	Line focus NA = 1.0	100 x 108 x 350	160 x 194 x 8.4	3.5 min x 12	488: 5ms/line (8 sec/plane), 560: 5ms/line (8 sec/plane)	Objective scan	R561; FF01-509/22, FF01-600/37	ISM reconstruction	ImageJ, Imaris
**S23**	TPE-Bessel light sheet	Yes	Mouse brain slice	cytosolic-YFP	37 °C	TP Bessel NA_{max}=0.58, NA_{min}=0.55	108 x 108 x 215	55 x 221 x 86	NA	920 nm: 1000 ms, 130 mW	Sample scan	T600dcrb; FF01-600/37, FF01-538/685	Deskewing, Deconvolution	ImageJ

Supplementary Table 2. List of abbreviations used in this manuscript.

Abbreviation	Definition
MOSAIC	Multimodal Optical Scope with Adaptive Imaging Correction
AO	Adaptive optics
LLSM	Lattice light-sheet microscopy
LLS-SIM	Lattice light-sheet structured-illumination microscopy
LLS-SPT	Lattice light-sheet single-particle tracking
SIM	Structured-illumination microscopy
3D-SIM	Three-dimensional structured-illumination microscopy
ISM	Image-scanning microscopy
OI	Oblique illumination (label-free)
TPM	Two-photon microscopy
AO-LLSM	Adaptive-optics lattice light-sheet microscopy
AO-TPM	Adaptive-optics two-photon microscopy
TP-Bessel	Two-photon Bessel-beam light-sheet microscopy
ExLLSM	Expansion lattice light-sheet microscopy
FOV	Field of view
NA	Numerical aperture
PSF	Point-spread function
OTF	Optical-transfer function
SNR	Signal-to-noise ratio
MIP	Maximum-intensity projection
MSD	Mean-squared displacement
ROI	Region of interest
SLM	Spatial-light modulator
DM	Deformable mirror
AF	Autofocus
AU	Airy unit
hpf / dpf	Hours / days post-fertilization (zebrafish)
mESC	Mouse embryonic stem cell
iPSC	Induced pluripotent stem cell
FUCCI	Fluorescent Ubiquitination-based Cell Cycle Indicator
DHB	a portion of human DNA Helicase B

Supplementary Text

DNA sequences for the zebrafish constructs used to label mitochondria and membrane

a. 4x-cox8-stayGold-ev-linker-stayGold

cox8

linker

stayGold

evl-linker

ATGAGTGGCCTGCTCCGGGGCCTGGCTCGCGTTAGAGCTGCACCTGTGCTTCGCGGA
TCGACAATCACACAGCGGGCCAACCTGGTGACCAGACCCGCCAAGGGAGATCCGGG
TCTATTACGTGGATTGGCACGTGTCAGAGCAGCCCCTGTACTTCGAGGATCCACTAT
TACGCAGAGGGCTAATTTGGTCACCCGCCCTGCTAAAGGTGACCCTGGTCTGTTGAG
AGGTCTTGCCAGGGTGCGAGCTGCCCCTGTGCTCAGGGGTAGCACCATCACGCAAC
GAGCAAACCTCGTGACGAGGCCAGCTAAGGGAGATCCCGGACTCTTGCGCGGACTG
GCGAGAGTGCGCGCGGCTCCCGTCCTTCGTGGCTCCACCATAACTCAGCGTGCCAAT
CTGGTCACAAGACCGGCCAAAGGGGATCCCATGGCATCTACAGGAGAGGAGCTCTT
CACTGGGGTTGTTCCGTTCAAATTTCAGCTCAAGGGCACCATCAACGGCAAATCCTT
CACCGTTGAAGGAGAGGGGGAAGGGAACAGCCATGAAGGAAGTCACAAAGGAAAG
TATGTCTGCACAAGCGGGAAACTGCCAATGTCTTGGGCCGCTCTGGGCACGAGTTTT
GGATATGGCATGAAATACTACACCAAATACCCATCTGGATTGAAAAACTGGTTTCAT
GAAGTGATGCCAGAGGGTTTCACTTATGACAGGCATATCCAGTATAAAGGAGATGG
CTCCATCCATGCCAAACATCAACACTTCATGAAGAATGGAACCTACCACAACATTGT
CGAGTTTACTGGTCAAGATTTTAAGGAGAATTCCCCAGTACTTACAGGTGATATGAA
TGTGTCTCTTCCAAACGAAGTTCAGCATATTCCAAGAGATGATGGAGTTGAGTGTCC
TGTTACTCTACTGTACCCTCTGCTATCCGACAAATCAAAGTGTGTGGAAGCTCACCA
GAACACAATATGCAAACCCTTACATAATCAGCCCGCGCCAGATGTTCCTTACCACTG
GATTAGGAAGCAATACACTCAAAGCAAAGATGACACAGAGGAAAGAGACCACATA
TGTCAGAGTGAGACCCTCGAGGCCCATCTGGAGACGCTGGAAGCCCATTTGTCTGCT
GGCGGTTCAGCCGGTGGCAGCGCCGGCGGTAGTGCAGGTGGTTCTGCTGGTGGTTCT
GCTGGGGGCAGCGCTGGCGGGAGTGCCGGCGGCAGCGCAGGTGGTTCGGCAGGTGG
GTCGGCAGGAGGCTCAGCTGGTGGATCAGCGGGAGGAAGCGCGGGTGGAAGCGCC
GGGGGCAGTGCAGGAGGCTCCGCCGGCGGATCAGCAGGGGGATCAGCTGGCGGCA
GTGCGGGAGGATCGGCAGGGGGCTCGGCTGGTGGGTCAGCTGGGGGATCCGCCGGA
GGTTCTGCGGGCGGCAGCGCTGGAGGGTCAGCTGGAGGAGCCAGCACAGGGGAGG
AGCTGTTCatgGCCTCTACTGGAGAAGAGCTTTTTACTGGAGTGGTGCCATTCAAGTTC
CAACTGAAAGGTACTATCAATGGCAAGAGCTTTACAGTAGAGGGCGAGGGCGAGGG
AAACAGTCATGAGGGGTCACACAAGGGGAAATATGTGTGCACTTCTGGAAAGCTGC
CTATGTCTTGGGCAGCATTGGGAACCTCCTTTGGATATGGAATGAAATATTATACCA
AGTATCCTTCTGGCCTGAAGAACTGGTTCCACGAGGTAATGCCTGAAGGCTTCACAT
ACGACAGACACATTCAGTACAAGGGTGATGGCAGTATTCATGCAAAACACCAGCAC
TTTATGAAGAACGGAACATATCACAATATAGTCGAGTTCACAGGACAGGACTTTAA
AGAAAACTCCCCGGTGCTCACAGGAGACATGAACGTCAGTCTCCCAAATGAGGTCC
AGCACATCCCACGAGATGACGGGGTGGAGTGCCCAGTAACTCTGCTTTACCCCCTGC

TGTCTGACAAGAGCAAATGTGTTGAAGCACATCAGAATACAATTTGTAAGCCCCTGC
ATAACCAGCCTGCTCCCGACGTGCCGTACCACTGGATCAGAAAGCAGTACACACAA
TCAAAAGACGACACTGAAGAACGGGATCACATCTGCCAGTCTGAAACCCTAGAGGC
ACACTTATGA

b. *eef1a1l1:mem-2x-mchilada*

eef1a1l1 promoter

lyn kinase myristoylation domain

linker

mchilada

SV40 polyA

GGCCAAAGGTTTGACAACATTAAGATTTGCTGATCTATTACAATCAATTGTTATGCT
TGTGAATAAATAATGGTTTTATTATGATTTTTATATTCAATTCATATTTCTGTAGCG
CTTTACAATGTAGATTGTGTCAAAGCAGCTTCACATAGAAGTTCAAGTAGATTGAAA
CTGTGTCAGTCCAGTTTTCAGAGTTGAAGTTCAGTTTAGTTTAGTGTGGTTTAATTTT
CACTGCGGAAAGTCCAAACACTGAAGAGCAAATCCGTCCATGCACAGCTCCACAAG
TCCCAAACCAAGCAAGCCAGAATTGCCTGTGATATACAGTTGAAGTCAGAATTATTA
GCCCCCCTGTTTATTTATTTTTTCTCAATTTCTGTTTAACGGGGAGACGATTTTTTTCA
ACACATTTCTAATAGTTTTAATAACTCATCTTTAATAACTGATTTATTTTATCTATGA
TGACAGTAAATAATATTAGACTAGATATTTTTCAAGACACTTCTATACAGCTTAAAG
TGACATTTAAAGGCTTAACTAGGTTAATTAGGGTAACTAGGCAGGTTAAGGGAATTA
GGCAAGTTATTGTATAACGATGGTTTGTTCTGTAGACTATCGAGAAAACAATATATA
ACTTAAAGGGGCTAATAATTTTGATTTTAAAATGGTGTTTAAAAAATGTAAAACTGC
TTTTATTCTAGCCGAAATAAAACAAAAAATATTATCAGACGTACTGTGACAATTTCC
TTGCTCTGTTAATCATTTGGGAATTATTTTAAAAAGAAAATAGCTTGAAAGGAAATT
TAATTTTATTTCAATTTTATGAAAGGAAACGTACTGTTTTGATGTGTTTCATTTTTCTG
CATTAAAATAATAATATATGTAACATTTGTTTTTATCAATCTTTGGACAGCCTAACAC
CATTGTTTTTTTTAAATCAACATTTGATCAAATAAATCTGATCACGACAAAATTAAG
CATTTGTTATTGTGATGAATATTAATTTTTCTAGAATTTAAGATAAAAAAGAAATGC
AAAAGTTAATATTTATATTTAGAAATTATGAAGAAATGTTGTCAGACTTTCATAGGA
TGAAACAAAAATTAAATAAAATATGAATAAGTAACATACTTTATTCAAACCTACGA
GTAAATCGAGAAATTTTCTCAAATAATATAGCCTACACTGACATTCATGTTAAGATG
GTCATCTTCTCCCTACTGCAGATTTAGTAGATATCCATCAGTTCTGTTCATCTCTTATT
ACATCTGCAATATTAGTAAATCTACTGTATGACACACTCAGATGATGGGCGAAACAA
AGTGGTGACGTCAGCCTGACGAGGCGGGGAGATTTTCAGTCAATTGTGTGTGATTTG
CGGGTCGATCAGGAAGCGGGGGGTTGCAGCCGGCCGCTCGGTCCTCCTCCCGCATA
TAAATTCTCCAACCAAAGCGTTTTTCTTCCTCTTTCTGTTACCTGGCAAAGGGGAGCA
GCAGCTGAGGAGTGATCTCTCAATCTTGGTGAGTACTATAGGCTAGGCCTAAGCTTT
TTAATTCTTGTATCGTTAACTTTAATGCGTATATTGAGGCTTTTCTTCACGGTGGCGG
ATGTTATTATGACATTTTCACGCGGCGGTTTGTTATCCGGCGAGCTTTTGTCAGTTAT
TTTATGTTCGGTTTTCTATGTGGCTTGTATGTCTTTCTTAAATGTTTTATTTTAATAAG
TAGTGCGTTTTAAAGTTGACTTTGTTGTCTCTTTATATTTCTGCTGTTTCCGTTATTTT

GTGTGTGAAATGAGCACCGGGAAAAGAACAAAGGCACAAGCGGTTTGAAGTCACG
GGTCCGTTAAAAGCCTATCACACATTCCCGCATCCAGCAACTGTTACAAGTGGCCGG
AGAGCCAGATGTCTGCCTCAGTTTGGATTCCGTTCAGACATTTCTGTTTTTTTTTAAT
CATCCTGTAACTTAACACCGTCTTACTCTAAAGAAACACGTGTCGCAGCTGCCTTAT
GGTGAACTAGGCCGGTTCAAACTCTGGGCCATCTTGTCACTTGTGTTACGAAATCAC
CGCTTAAAAGTTTAATAATGAAAAATATGTATTTTTGTGGTCCATCATTAACTTTCA
CATTTTATCTTGGTCCGCAGAAACTTATCAATCaccatgggctgcatcaagagcaagcgcaaggacaacct
gaacgacgacgaggccgcAatgggctgCatAaagagcaagcgAaaggacaacctgaacgaTgacgagggAGCGGCaGc
AcccGCcGcTGCAagcgtTagcaagggcgaggagGACAACATGGCTATCATCAAGGAATACATGC
GTTTCAAaGTTCATATGGAAGGaTCTGTTAACGGTCATGAATTCGAAATCGAAGGcGA
AGGcGAAGGTCGTCCTTTCGAgGGaACCCAGACCGCTAAGCTGAAaGTTACCAAGGGT
GGTCCTCTGCCTTTCGCTTGGCATATCCTGCCTCCTCAaTTCCAaTACGGTTCTAAGGC
TTACGTTAAGCATCCTGCTGACATCCCTGACTACTTCAAaCTgTCaTTCCCtGAgGGTTT
CACCTGGGAACGTGAAATGAACTTCGAAGACGGTGGTGTTGTTACCGTgACaCAaGA
CTCaTCaCTcCAaGACGGaGAATTCATCTACAAaGTTAAGCTGCGTGGaACCAACTTCCC
TTCTGACGGTCCTGTTATGCAaAAGAAGACCATGGGcAACACCGCTTCTACCGAACGt
ATGTAtCCTGAAGACGGTGCTCTGAAGGGTGAAACCAAGTGGCGTCTGAAGCTGAAG
GACGGTGGTCATTACGAAGCTGAAGTTAAGACCACCTACAAGGCTAAGAAGCCTGT
TCAGCTGCCTGGTGCTTACAACGTTGACCGcAAGCTGAAGATCACCTACCATAACGA
AGACTACACCATCGTTGAACAGTACGAACGTGCTGAAGCTCGTCATTCTACCGGTggc
atggacgagctgtacaagggcGGatccggtggatccggtggaAGCgtTagcaagggcgaggagGACAACATGGCTA
TCATCAAGGAATACATGCGTTTCAAaGTTCATATGGAAGGaTCTGTTAACGGTCATGA
ATTCGAAATCGAAGGcGAAGGcGAAGGTCGTCCTTTCGAgGGaACCCAGACCGCTAA
GCTGAAaGTTACCAAGGGTGGTCCTCTGCCTTTCGCTTGGCATATCCTGCCTCCTCAa
TTCCAaTACGGTTCTAAGGCTTACGTTAAGCATCCTGCTGACATCCCTGACTACTTCA
AaCTgTCaTTCCCtGAgGGTTTCACCTGGGAACGTGAAATGAACTTCGAAGACGGTGGT
GTTGTTACCGTgACaCAaGACTCaTCaCTcCAaGACGGaGAATTCATCTACAAaGTTAAG
CTGCGTGGaACCAACTTCCCTTCTGACGGTCCTGTTATGCAaAAGAAGACCATGGGcA
ACACCGCTTCTACCGAACGtATGTAtCCTGAAGACGGTGCTCTGAAGGGTGAAACCA
AGTGGCGTCTGAAGCTGAAGGACGGTGGTCATTACGAAGCTGAAGTTAAGACCACC
TACAAGGCTAAGAAGCCTGTTCAGCTGCCTGGTGCTTACAACGTTGACCGcAAGCTG
AAGATCACCTACCATAACGAAGACTACACCATCGTTGAACAGTACGAACGTGCTGA
AGCTCGTCATTCTACCGGTggcatggacgagctgtacaagtaAgaattcatacgtatccggaaccggtgatccagacat
gataagatacattgatgagtttggacaaaccacaactagaatgcagtgaaaaaaatgctttatttgtgaaatttgtgatgctattgctttatttgtaa
ccattataagctgcaataaacaagttaacaacaacaattgcattcattttatgtttcaggttcagggggaggtgtgggaggtttttt
